# Characterization of the degree of food processing in the European Prospective Investigation into Cancer and Nutrition: application of the Nova classification and validation using selected biomarkers of food processing

**DOI:** 10.3389/fnut.2022.1035580

**Published:** 2022-12-16

**Authors:** Inge Huybrechts, Fernanda Rauber, Geneviève Nicolas, Corinne Casagrande, Nathalie Kliemann, Roland Wedekind, Carine Biessy, Augustin Scalbert, Mathilde Touvier, Krasimira Aleksandrova, Paula Jakszyn, Guri Skeie, Rashmita Bajracharya, Jolanda M. A. Boer, Yan Borné, Veronique Chajes, Christina C. Dahm, Lucia Dansero, Marcela Guevara, Alicia K. Heath, Daniel B. Ibsen, Keren Papier, Verena Katzke, Cecilie Kyrø, Giovanna Masala, Esther Molina-Montes, Oliver J. K. Robinson, Carmen Santiuste de Pablos, Matthias B. Schulze, Vittorio Simeon, Emily Sonestedt, Anne Tjønneland, Rosario Tumino, Yvonne T. van der Schouw, W. M. Monique Verschuren, Beatrice Vozar, Anna Winkvist, Marc J. Gunter, Carlos A. Monteiro, Christopher Millett, Renata Bertazzi Levy

**Affiliations:** ^1^Nutrition and Metabolism Branch, International Agency for Research on Cancer, Lyon, France; ^2^Department of Preventive Medicine, School of Medicine, University of São Paulo, São Paulo, Brazil; ^3^Center for Epidemiological Research in Nutrition and Health, University of São Paulo, São Paulo, Brazil; ^4^Sorbonne Paris Nord University, INSERM U1153, INRAE U1125, CNAM, Nutritional Epidemiology Research Team (EREN), Epidemiology and Statistics Research Center, University Paris Cité (CRESS), Paris, France; ^5^Department of Epidemiological Methods and Etiological Research, Leibniz Institute for Prevention Research and Epidemiology (BIPS), Bremen, Germany; ^6^Human and Health Sciences, University of Bremen, Bremen, Germany; ^7^Unit of Nutrition and Cancer, Cancer Epidemiology Research Programme, Catalan Institute of Oncology (ICO-IDIBELL), Barcelona, Spain; ^8^Blanquerna School of Health Sciences, Ramon Llull University, Barcelona, Spain; ^9^Department of Community Medicine, UiT the Arctic University of Norway, Tromsø, Norway; ^10^German Cancer Research Center (DKFZ), Heidelberg, Germany; ^11^Centre for Nutrition, Prevention and Health Services, National Institute for Public Health and the Environment (RIVM), Bilthoven, Netherlands; ^12^Department of Clinical Sciences Malmö, Faculty of Medicine, Nutritional Epidemiology, Lund University, Lund, Sweden; ^13^Department of Public Health, Aarhus University, Aarhus, Denmark; ^14^Department of Clinical and Biological Sciences, Centre for Biostatistics, Epidemiology, and Public Health (C-BEPH), University of Turin, Turin, Italy; ^15^Instituto de Salud Pública de Navarra, Pamplona, Spain; ^16^Centro de Investigación Biomédica en Red de Epidemiología y Salud Pública (CIBERESP), Madrid, Spain; ^17^Navarra Institute for Health Research (IdiSNA), Pamplona, Spain; ^18^Department of Epidemiology and Biostatistics, School of Public Health, Imperial College London, London, United Kingdom; ^19^Steno Diabetes Center Aarhus, Aarhus, Denmark; ^20^MRC Epidemiology Unit, University of Cambridge School of Clinical Medicine, Cambridge, United Kingdom; ^21^Department of Nutrition, Exercise and Sports, University of Copenhagen, Frederiksberg, Denmark; ^22^Cancer Epidemiology Unit, Nuffield Department of Population Health, University of Oxford, Oxford, United Kingdom; ^23^Danish Cancer Society Research Center, Danish Cancer Society, Copenhagen, Denmark; ^24^Clinical Epidemiology Unit, Institute for Cancer Research, Prevention and Clinical Network (ISPRO), Florence, Italy; ^25^Department of Nutrition and Food Science, Campus of Cartuja, University of Granada, Granada, Spain; ^26^CIBER of Epidemiology and Public Health (CIBERESP), Madrid, Spain; ^27^Instituto de Investigación Biosanitaria ibs.GRANADA, Granada, Spain; ^28^Biomedical Research Centre, Institute of Nutrition and Food Technology (INYTA) “José Mataix”, University of Granada, Granada, Spain; ^29^MRC Centre for Environment and Health, School of Public Health, Imperial College London, London, United Kingdom; ^30^Department of Epidemiology, Murcia Regional Health Council, IMIB-Arrixaca, Murcia, Spain; ^31^Department of Molecular Epidemiology, German Institute of Human Nutrition Potsdam-Rehbruecke, Nuthetal, Germany; ^32^Institute of Nutritional Science, University of Potsdam, Potsdam, Germany; ^33^Dipartimento di Salute Mentale e Fisica e Medicina Preventiva, Vanvitelli University, Naples, Italy; ^34^Hyblean Association for Epidemiological Research, AIRE ONLUS, Ragusa, Italy; ^35^Julius Center for Health Sciences and Primary Care, University Medical Center Utrecht, Utrecht University, Utrecht, Netherlands; ^36^Sustainable Health, Department Public Health and Clinical Medicine, Umeå University, Umeå, Sweden; ^37^Department of Internal Medicine and Clinical Nutrition, Sahlgrenska Academy, University of Gothenburg, Gothenburg, Sweden; ^38^Department of Nutrition, School of Public Health, University of São Paulo, São Paulo, Brazil; ^39^Public Health Policy Evaluation Unit, School of Public Health, Imperial College London, London, United Kingdom

**Keywords:** food processing, Nova, EPIC, biomarkers, elaidic acid, syringol

## Abstract

**Background:**

Epidemiological studies have demonstrated an association between the degree of food processing in our diet and the risk of various chronic diseases. Much of this evidence is based on the international Nova classification system, which classifies food into four groups based on the type of processing: (1) Unprocessed and minimally processed foods, (2) Processed culinary ingredients, (3) Processed foods, and (4) “Ultra-processed” foods (UPF). The ability of the Nova classification to accurately characterise the degree of food processing across consumption patterns in various European populations has not been investigated so far. Therefore, we applied the Nova coding to data from the European Prospective Investigation into Cancer and Nutrition (EPIC) in order to characterize the degree of food processing in our diet across European populations with diverse cultural and socio-economic backgrounds and to validate this Nova classification through comparison with objective biomarker measurements.

**Methods:**

After grouping foods in the EPIC dataset according to the Nova classification, a total of 476,768 participants in the EPIC cohort (71.5% women; mean age 51 [standard deviation (SD) 9.93]; median age 52 [percentile (p)25–p75: 58–66] years) were included in the cross-sectional analysis that characterised consumption patterns based on the Nova classification. The consumption of food products classified as different Nova categories were compared to relevant circulating biomarkers denoting food processing, measured in various subsamples (N between 417 and 9,460) within the EPIC cohort via (partial) correlation analyses (unadjusted and adjusted by sex, age, BMI and country). These biomarkers included an industrial transfatty acid (ITFA) isomer (elaidic acid; exogenous fatty acid generated during oil hydrogenation and heating) and urinary 4*-*methyl syringol sulfate (an indicator for the consumption of smoked food and a component of liquid smoke used in UPF).

**Results:**

Contributions of UPF intake to the overall diet in % grams/day varied across countries from 7% (France) to 23% (Norway) and their contributions to overall % energy intake from 16% (Spain and Italy) to >45% (in the UK and Norway). Differences were also found between sociodemographic groups; participants in the highest fourth of UPF consumption tended to be younger, taller, less educated, current smokers, more physically active, have a higher reported intake of energy and lower reported intake of alcohol. The UPF pattern as defined based on the Nova classification (group 4;% kcal/day) was positively associated with blood levels of industrial elaidic acid (*r* = 0.54) and 4*-*methyl syringol sulfate (*r* = 0.43). Associations for the other 3 Nova groups with these food processing biomarkers were either inverse or non-significant (e.g., for unprocessed and minimally processed foods these correlations were –0.07 and –0.37 for elaidic acid and 4*-*methyl syringol sulfate, respectively).

**Conclusion:**

These results, based on a large pan-European cohort, demonstrate sociodemographic and geographical differences in the consumption of UPF. Furthermore, these results suggest that the Nova classification can accurately capture consumption of UPF, reflected by stronger correlations with circulating levels of industrial elaidic acid and a syringol metabolite compared to diets high in minimally processed foods.

## Introduction

Worldwide there has been a dramatic increase in the production of industrially processed foods which has coincided with a growing prevalence of obesity, metabolic disorders and multiple chronic diseases (1–16). Global industrialisation, during which diets have been shifting from fresh unprocessed and minimally processed foods toward an increase in the consumption of “ultra-processed” foods (UPF), has been implicated as an important driver of these worrying trends in metabolic disease. UPF that undergo multiple physical, biological, and/or chemical processes, generally contain various processing contaminants, food additives or other industrial substances (17, 18), while they are on average poorer in protective micronutrients (e.g., anti-oxidants) compared to fresh foods (19–33).

The Nova classification system was developed in response to the increased recognition of the importance of classifying foods according to their degree and purpose of processing (i.e., un/minimally processed, processed and ultra-processed foods, as well as culinary ingredients) rather than in terms of nutrients (17, 34, 35). However, recent publications criticized the concepts and definitions used for the Nova classification (36–38), requesting further validation of this food processing classification. While consistent epidemiological evidence linking the consumption of UPF (Nova group 4) to adverse health outcomes such as obesity, type 2 diabetes, cardiovascular diseases, and some cancers is accumulating (3–16), an in depth validation of the Nova classification through comparison with food processing biomarkers is indeed still lacking in population studies.

The European Prospective Investigation into Cancer and Nutrition (EPIC) study offers an appropriate framework to investigate the validity of the Nova classification through comparison with food processing biomarkers already available, namely industrial trans-fatty acids (ITFA) measured in blood (39) and a methylsyringol metabolite measured in urine (40). UPF are the main source of industrially transformed fats, such as partially hydrogenated fats containing industrial trans-fatty acids (ITFA) (11, 41, 42). As such, ITFA profiles in blood may represent a reliable biomarker for UPF consumption. Also, the urinary biomarker 4-methylsyringol sulfate, can be used as an indicator for the consumption of smoked food as it is the human metabolite of 4-methyl syringol, which is formed by the combustion of wood during smoking and deposited on smoked foods (40) and often added as part of liquid smoke to UPF (such as hot dogs) to generate a smoky flavor. A previous intervention study (40) confirmed that syringol markers in urine were detected after intake of ultra-processed hot dogs and to a lower extent after intake of bacon (40). These differences observed after consumption of hot dogs and bacon might be explained by the use of liquid smoke in ultra-processed hot dogs.

The consumption of products from the different Nova categories in relation to relevant biomarkers that are related to food processing such as circulating ITFA has not been evaluated so far (11, 41). To address this gap, we aimed to evaluate UPF consumption patterns in relation to food processing biomarkers available in EPIC as objective indicators of dietary intakes. We hypothesize a positive association of the consumption of UPF with ITFA profiles in blood and syringol markers in urine.

## Materials and methods

### Cohort description

EPIC is a multi-center prospective cohort study, designed to investigate the relationship between nutrition and cancer, among other diseases. A detailed description of the EPIC cohort, including study populations and data collection, has been previously described elsewhere (43). Briefly, it consists of 23 study centers in 10 European countries, including, Denmark, France, Germany, Greece, Italy, Netherlands, Norway, Spain, Sweden, and the United Kingdom. Participants were mostly from the general population and recruited between 1991 and 2000. All participants provided written informed consent and the ethical review boards from the International Agency for Research on Cancer (IARC) and all local centers approved the study. Data from Greece were not available for these analyses.

At baseline, information on lifestyle, dietary intake and medical information as well as sociodemographic and anthropometric data were collected. Lifestyle and medical history questionnaires were used to obtain information on education, smoking status and intensity, alcohol consumption, diabetes and women’s health (including menopausal status, oral contraceptive use, hormone replacement therapy use, age at menarche and age at first full-term pregnancy). Physical activity levels were estimated using a questionnaire focused on past-year physical activity in occupational, leisure and household domains and classified according to the validated Cambridge physical activity index (44).

Body weight and height were measured in all centers, except for Oxford (UK), France and Norway where these were self-reported. Anthropometric characteristics were measured by trained observers using standardized methods (43, 45). Weight and height were used to calculate body mass index (BMI) defined as weight in kilograms divided by height in meters squared (kg/m^2^).

Diet was assessed at study baseline using validated country/center-specific methods, including dietary questionnaires (DQs) spanning the previous 12 months (43). In most centers, DQs were self-administered, with the exception of Ragusa (Italy), Naples (Italy) and Spain, where face-to-face interviews were performed. Extensive quantitative DQs were used in northern Italy, and Germany that were structured by meals in Spain, France and Ragusa. Semi-quantitative food-frequency questionnaires (FFQs) were used in Denmark, Netherlands, Norway, Italy, Umeå (Sweden) and the United Kingdom, while a FFQ was combined with a 7-day record on hot meals in Malmö (Sweden). Relying on a common food classification and standard handling of recipes, post-harmonization of all the questionnaire data was done by following standardized procedures (e.g., decomposing local recipes and complex foods into ingredients) to obtain a standardized food list for which the level of detail is more comparable between countries (except for Malmö and Spain where open dietary intake assessment methods were used with a higher level of detail; see Supplementary Table 1 for overview of dietary assessment methods used in the different countries/centers). This standardized food list includes more than 11,000 food items. No brand name information was available in the EPIC dietary questionnaires, although some centers asked for the most frequent brand names or product names, e.g., for breakfast cereals in the UK and for margarines in the Netherlands.

From the initial pool of 521,323 EPIC participants, we excluded subjects with missing dietary and/or lifestyle information (*n* = 6,837), Greek participants (*N* = 28,034) due to data access issues, and 9,684 participants in the top or bottom 1% of the ratio of energy intake to energy requirement, leaving a final sample of 476,768 adults.

### Nova classification

We classified all recorded food items from the EPIC questionnaires according to the Nova food classification system based on the nature, extent, and purpose of industrial food processing (17, 35). This coding was done in close collaboration with the team of Dr. Carlos Monteiro, University of São Paulo (USP), the founder of the Nova classification system. In summary, the Nova classification includes four processing groups and subgroups were adapted to the EPIC items (see Supplementary Table 2).

(1) Group 1: unprocessed or minimally processed foods, which are natural foods (edible parts of plants or of animals after separation from nature) and natural foods altered by methods such as freezing, pasteurization, fermentation, grinding, and other methods that do not include the addition of substances such as salt, sugar and/or oils or fats (e.g., fresh, dry or frozen fruits or vegetables; grains, flours and pasta; pasteurized/sterilized or powdered plain milk; plain yogurt; fresh or frozen meat);
(2) Group 2: processed culinary ingredients are extractions of fresh foods or elements of nature, including substances obtained directly from group 1 foods or from nature by processes that include pressing, refining, grinding, milling, and drying, and consumed in combination with group 1 foods in freshly prepared dishes (e.g., table sugar; oils; butter; cream and salt);
(3) Group 3: processed foods, which are products manufactured industrially with the addition of culinary ingredients (e.g., salt, sugar, oil or fats) to unprocessed or minimally processed foods. Examples of Nova group 3 include canned vegetables; traditional cheese; traditional bread; smoked fish; plain sweetened yogurt;
(4) Group 4: ultra-processed foods, which are commercial food and drink formulations containing besides salt, sugar or fats other substances derived from foods but not domestically used as culinary ingredients (such as protein isolates, hydrogenated oils, modified starches), flavors or additives designed to make the final product palatable or more appealing, such as colors, sweeteners, and emulsifiers. Examples of Nova group 4 include industrially produced bread, poultry and fish nuggets and sticks and other reconstituted meat products transformed with addition of preservatives other than salt; instant noodles and dehydrated soups; carbonated diet and regular sodas; chocolate with emulsifiers, chewing gums and candies with dyes (confectionery); margarine; instant desserts; most breakfast “cereals,” “energy” bars; “energy” drinks; flavored milk drinks/yogurts; sweet desserts made from fruit with added sugars, artificial flavors and texturizing agents; cooked seasoned vegetables with ready-made sauces; vegetable patties (meat substitutes) containing food additives; “health” and “slimming” products such as powdered or “fortified” meal and dish substitutes (see Supplementary Table 2).

We identified homemade and artisanal food preparations, based on FFQ food names and/or local habits. Those identified as homemade recipes were decomposed using local recipes, and the Nova classification was applied to their ingredients. This disaggregation in ingredients was essential to correctly assess the consumption of culinary ingredients (Nova group 2). For breads, data from the Low Energy Ovens Project (46) were used at the country level and a visual check was performed at the DQ item level (e.g., usual Italian and French breads were considered artisanal, while UK bread was classified as ultra-processed). The very detailed EPIC 24-h recalls calibration data (47) and the website Open Food Facts^1^ were also considered as sources of information on the degree of processing in the different EPIC countries (48).

### The transition of food processing over the past decades: Creation of scenarios

Changes in the practice of food processing over the past decades require the use of different scenarios when classifying foods according to the Nova classification in a long-term follow-up cohort like EPIC. Dietary intakes were collected at baseline, while the food environment has changed in the intervening years, exposing the EPIC participants to potentially different degrees of food processing over the course of their follow-up (e.g., certain products that were still prepared at home during the 1990s have been replaced by industrial products). As such, a particular food item can potentially be classified in different Nova groups depending on the time period. Therefore, we created three possible scenarios. The “most likely scenario,” in food safety terminology often called the middle bound scenario (MB), which is the scenario considering the most common food processing environment around the baseline period, was used as the main scenario (as agreed upon between the USP team and the IARC team). However, as we were unsure about the level of processing for some of the food items (e.g., when insufficient level of detail was available) for the period 1990–2020, we decided to introduce two alternative scenarios, namely a lower bound (LB) scenario reflecting the lowest degree of processing, and a more processed or upper bound (UB) scenario. For the lower bound scenario, some foods were classified in a less processed Nova group compared to the middle bound scenario when the food item may also have been prepared at home or in an artisanal setting instead of being industrially produced. For the upper bound scenario, some food items were classified in a more processed Nova group compared to the middle bound scenario when it was possible that the food item could be more processed than the most likely option assigned in the middle bound scenario. An example of these three scenarios used for the Nova classification is given in Supplementary Table 3.

### Quality controls to evaluate the performance of the Nova classification in the European Prospective Investigation into Cancer and Nutrition

The coding of the Nova classification has been evaluated and checked via different quality controls (e.g., comparing the Nova coding proposed by independent food coders, systematic and logic quality controls, checking if the sum of Nova subgroups is equal to the attached Nova group, etc.). One of the quality controls was the comparison with an independent coding performed by the Spanish team in Barcelona on their food list from the Spanish EPIC cohort. The Nova coding performed by the international team was compared with the coding applied in Spain for the Spanish food list. Differences between these two classifications have been discussed between the two teams and few corrections to the three scenarios were made based upon this quality control.

### Evaluation of the Nova classification through comparison with processing biomarkers

To evaluate the validity of the Nova classification in EPIC, we investigated correlations between the different Nova categories and food processing biomarkers available in subsets of EPIC participants (calibration study and nested case-control studies) analysed in biospecimens collected around the time that the baseline questionnaires were collected. ITFA (elaidic acid levels) measured in plasma phospholipids (49) have been used as biomarkers of dietary intake of industrial trans-fat which is mainly found in UPFs (according to Nova, the presence in the list of ingredients of partially hydrogenated oils, which provide industrial trans fats, makes the product be classified as ultra-processed). Fatty acid profiling was performed using a method previously described (49). ITFA was quantified using an Agilent 7890 gas chromatograph instrument (Agilent Technologies, Santa Clara, CA, USA), and concentrations were expressed as the percentage of total fatty acids (*n* = 9,460). Elaidic acid was the only ITFA measured in EPIC and as such used as a biomarker for industrially produced foods in these validation analyses.

*4*-Methyl syringol sulfate which has recently been proposed as a biomarker of smoked meat intake (40) was measured in 24 h urine samples (*n* = 417) from the EPIC calibration study that included samples from Italy, France and Germany. Sample preparation, laboratory measurement and data processing is described elsewhere (40).

### Statistical analysis

All analyses were performed using the three scenarios for the Nova classification (the lower, middle and upper bound scenarios, representing changes in the food environment over time). Baseline characteristics were examined for the total population and by sex-specific quartiles of each Nova food group. The potential differences between participants were assessed using analysis of variance or χ2 tests when appropriate. Descriptive analyses were performed for each Nova food group considering their daily actual and relative intake in grams and kcal.

Pearson correlations were used to evaluate the association between the Energy % from UPF obtained via the Nova coding performed by the Spanish team (considered as the middle bound scenario) versus those obtained via the three codings performed by the international team for the Spanish food list. In addition, weighted kappa statistics were used to investigate agreement between these two independent codings of the Spanish food list.

Pearson and Spearman correlations were used to investigate associations of levels of biomarkers with % grams and % energy derived from the four Nova groups. Sensitivity analyses were run using partial correlations adjusted for sex, age, BMI and country.

In addition, we also ran sensitivity analyses for the Nova 3 and Nova 4 groups while excluding the alcoholic beverages from these two Nova groups in order to investigate associations between the Nova group intakes and the food processing biomarkers while eliminating the effect of alcohol.

### Data availability

EPIC data and biospecimens are available for investigators who seek to answer important questions on health and disease in the context of research projects that are consistent with the legal and ethical standard practices of IARC/WHO and the EPIC centres. The primary responsibility for accessing the data, including the Nova categories obtained in the frame of the present publication, belongs to the EPIC centres that provided them. The use of a random sample of anonymised data from the EPIC study can be requested by contacting epic@iarc.fr. The request will then be passed to members of the EPIC Steering Committee for deliberation.

## Results

**FIGURE 1:**
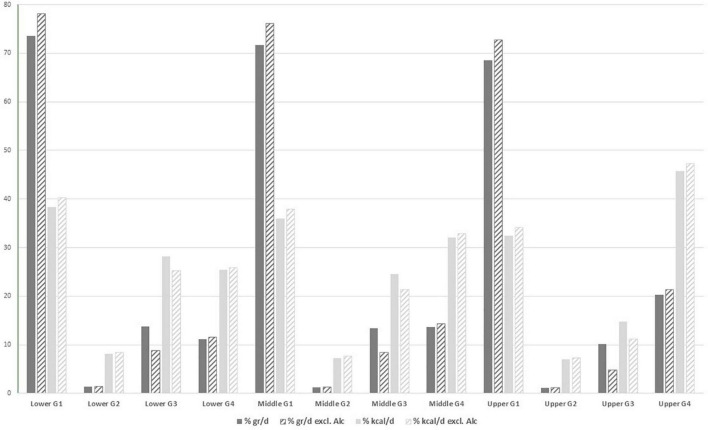
Distributions of the different Nova categories, group G1 to G4, using the three different scenarios (lower, middle and upper bound) for the total EPIC cohort (*N* = 476,768; distributions per country are provided as Supplementary Tables and Figures). Results derived from the Nova classification in which alcoholic drinks were excluded have been shaded in lighter color font.

A total of 476,768 participants were included in the analysis (71.5% women) investigating characteristics of the degree of food processing in EPIC. The mean and median age of participants at recruitment were 51 (SD 9.93) years and 52 (p_25–75_: 58–66) years, respectively (Table 1). Supplementary Table 4 presents the distributions of the different Nova groups for the total EPIC cohort using the three different scenarios and expressed in both grams and kcal (absolute and relative values) per day. A visual presentation is given in Figure 1. When looking at intakes expressed as grams per day, most of the intakes are from Nova group 1 (Nova group 1 intake is more than 6 times the amount of the processed and UPF groups), while the contributions from the processed and ultra-processed foods (Nova groups 3 and 4) are rather comparable, and Nova group 2 contributing less. The intakes expressed as kcal are rather comparable between the Nova groups 1, 3 and 4, while far lower for Nova group 2 (culinary ingredients). UPF intake contributed to 14% of the total diet in grams/day and to 32% of total daily energy intake. Differences in the consumption of ultra-processed foods were found between socio-demographic groups (Table 1). Although there was a higher proportion of women in this cohort, the contribution of UPF to the overall diet was very similar between men and women. Compared with the lowest fourth of UPF consumption, participants in the highest fourth of UPF consumption tended to be younger, taller, more often current smokers, more physically active, have a lower level of attained education, higher intakes of energy, fat and carbohydrates and lower intake of alcohol (see Table 1). In addition, the FSAm-NPS Dietary Index (DI) score (50), for which a higher score reflects an overall lower nutritional quality of consumed foods, increased with increasing fourth of UPF intake. Supplementary Tables 6A–C present the characteristics of the study population by sex-specific quartiles of relative intake for Nova groups 1 to 3. Subjects in the higher quartiles for diets rich in fresh and minimally processed foods (Nova group 1;% kcal/day) had higher Mediterranean diet scores (51) (Supplementary Table 6A).

**TABLE 1:** Baseline characteristics by sex-specific quartiles of relative intakes of Nova group 4 – ultra-processed foods (% g/day and % kcal/day including alcohol).

Characteristics	Nova group 4 quartiles in %g/d								Nova group 4 quartiles in %kcal/d							
		
	1st		2nd		3rd		4th		1st		2nd		3rd		4th	
								
	Mean or *N*	(SD) (%)	Mean or *N*	(SD) (%)	Mean or *N*	(SD) (%)	Mean or *N*	(SD) (%)	Mean or *N*	(SD) (%)	Mean or *N*	(SD) (%)	Mean or *N*	(SD) (%)	Mean or *N*	(SD) (%)
**Sex [*n* (%)]**																
Male	33,982	(28.5)	33,982	(28.5)	33,983	(28.5)	33,982	(28.5)	33,982	(28.5)	33,982	(28.5)	33,983	(28.5)	33,982	(28.5)
Female	85,209	(71.5)	85,210	(71.5)	85,210	(71.5)	85,210	(71.5)	85,209	(71.5)	85,210	(71.5)	85,210	(71.5)	85,210	(71.5)
Age, years [mean (SD)]	53.0	(7.8)	52.6	(8.9)	51.3	(10.2)	48.3	(11.2)	51.7	(7.8)	51.3	(9.3)	51.6	(10.4)	50.7	(11.2)
Height, cm [mean (SD)]	164.2	(8.6)	166.0	(8.9)	166.8	(8.8)	167.6	(8.5)	163.2	(8.4)	166.2	(9.2)	167.5	(8.6)	167.7	(8.3)
BMI, kg/m^2^ [mean (SD)]	25.4	(4.3)	25.2	(4.1)	25.2	(4.1)	25.2	(4.3)	25.5	(4.4)	25.1	(4.1)	25.2	(4.1)	25.2	(4.2)
**Education [*n* (%)]**																
None	8,806	(7.4)	3,187	(2.7)	2,275	(1.9)	1,676	(1.4)	11,083	(9.3)	3,268	(2.7)	1,055	(0.9)	538	(0.5)
Primary school completed	32,359	(27.1)	30,343	(25.5)	28,176	(23.6)	26,527	(22.3)	35,957	(30.2)	26,927	(22.6)	26,547	(22.3)	27,974	(23.5)
Technical/professional school	14,833	(12.4)	26,155	(21.9)	32,006	(26.9)	36,636	(30.7)	10,794	(9.1)	23,641	(19.8)	35,533	(29.8)	39,662	(33.3)
Secondary school	30,779	(25.8)	25,207	(21.1)	21,854	(18.3)	21,937	(18.4)	31,626	(26.5)	28,481	(23.9)	21,509	(18.0)	18,161	(15.2)
Longer education	29,776	(25.0)	30,712	(25.8)	29,079	(24.4)	25,884	(21.7)	27,504	(23.1)	33,880	(28.4)	29,623	(24.9)	24,444	(20.5)
Not specified	2,638	(2.2)	3,588	(3.0)	5,803	(4.9)	6,532	(5.5)	2,227	(1.9)	2,995	(2.5)	4,926	(4.1)	8,413	(7.1)
**Smoking status [*n* (%)]**																
Never	60,559	(50.8)	57,817	(48.5)	56,714	(47.6)	56,548	(47.4)	63,901	(53.6)	60,537	(50.8)	54,407	(45.6)	52,793	(44.3)
Former	30,197	(25.3)	33,231	(27.9)	34,381	(28.8)	32,815	(27.5)	27,842	(23.4)	32,369	(27.2)	35,801	(30.0)	34,612	(29.0)
Current	25,728	(21.6)	26,222	(22.0)	26,153	(21.9)	27,255	(22.9)	24,973	(21.0)	24,102	(20.2)	27,097	(22.7)	29,186	(24.5)
Unknown	2,707	(2.3)	1,922	(1.6)	1,945	(1.6)	2,574	(2.2)	2,475	(2.1)	2,184	(1.8)	1,888	(1.6)	2,601	(2.2)
**Smoking intensity [*n* (%)]**																
Never	46,551	(39.1)	48,903	(41.0)	51,975	(43.6)	54,501	(45.7)	48,482	(40.7)	49,733	(41.7)	51,504	(43.2)	52,211	(43.8)
Current, 1–15 cig/day	11,806	(9.9)	13,894	(11.7)	14,388	(12.1)	15,555	(13.1)	11,440	(9.6)	12,985	(10.9)	15,202	(12.8)	16,016	(13.4)
Current, 16–25 cig/day	7,155	(6.0)	7,197	(6.0)	7,246	(6.1)	7,669	(6.4)	7,212	(6.1)	6,560	(5.5)	7,333	(6.2)	8,162	(6.8)
Current, 26 + cig/day	2,275	(1.9)	1,692	(1.4)	1,441	(1.2)	1,464	(1.2)	2,474	(2.1)	1,717	(1.4)	1,328	(1.1)	1,353	(1.1)
Former, quit = 10 years	11,031	(9.3)	11,198	(9.4)	11,691	(9.8)	12,150	(10.2)	10,986	(9.2)	11,026	(9.3)	11,945	(10.0)	12,113	(10.2)
Former, quit 11–20 years	9,728	(8.2)	10,291	(8.6)	10,420	(8.7)	9,516	(8.0)	9,523	(8.0)	10,275	(8.6)	10,595	(8.9)	9,562	(8.0)
Former, quit 20 + years	8,332	(7.0)	10,391	(8.7)	10,896	(9.1)	9,812	(8.2)	6,569	(5.5)	9,827	(8.2)	11,799	(9.9)	11,236	(9.4)
Current, pipe/cigar/occas	18,971	(15.9)	12,180	(10.2)	7,155	(6.0)	3,840	(3.2)	19,768	(16.6)	13,430	(11.3)	5,439	(4.6)	3,509	(2.9)
Current/Former, missing	3,342	(2.8)	3,446	(2.9)	3,981	(3.3)	4,685	(3.9)	2,737	(2.3)	3,639	(3.1)	4,048	(3.4)	5,030	(4.2)
**Physical Activity [*n* (%)]**																
Inactive	29,230	(24.5)	23,508	(19.7)	21,859	(18.3)	19,626	(16.5)	32,060	(26.9)	22,469	(18.9)	19,312	(16.2)	20,382	(17.1)
Moderately inactive	42,406	(35.6)	41,380	(34.7)	39,148	(32.8)	36,326	(30.5)	43,300	(36.3)	41,104	(34.5)	38,288	(32.1)	36,568	(30.7)
Moderately active	29,003	(24.3)	29,926	(25.1)	31,454	(26.4)	36,512	(30.6)	27,652	(23.2)	31,064	(26.1)	32,541	(27.3)	35,638	(29.9)
Active	18,001	(15.1)	22,721	(19.1)	23,502	(19.7)	22,756	(19.1)	15,781	(13.2)	22,017	(18.5)	25,618	(21.5)	23,564	(19.8)
Missing	551	(0.5)	1,657	(1.4)	3,230	(2.7)	3,972	(3.3)	398	(0.3)	2,538	(2.1)	3,434	(2.9)	3,040	(2.6)
**Hypertension [*n* (%)]**																
No	86,118	(72.3)	76,280	(64.0)	72,812	(61.1)	77,860	(65.3)	93,510	(78.5)	78,960	(66.2)	68,386	(57.4)	72,214	(60.6)
Yes	22,795	(19.1)	21,828	(18.3)	21,352	(17.9)	19,694	(16.5)	23,261	(19.5)	23,716	(19.9)	21,552	(18.1)	17,140	(14.4)
Do not know	10,278	(8.6)	21,084	(17.7)	25,029	(21.0)	21,638	(18.2)	2,420	(2.0)	16,516	(13.9)	29,255	(24.5)	29,838	(25.0)
**Hyperlipidaemia [*n* (%)]**																
No	81,417	(68.3)	69,363	(58.2)	63,533	(53.3)	55,946	(46.9)	89,347	(75.0)	71,914	(60.3)	58,837	(49.4)	50,161	(42.1)
Yes	20,534	(17.2)	14,916	(12.5)	12,770	(10.7)	10,304	(8.6)	23,474	(19.7)	16,347	(13.7)	11,033	(9.3)	7,670	(6.4)
Do not know	17,240	(14.5)	34,913	(29.3)	42,890	(36.0)	52,942	(44.4)	6,370	(5.3)	30,931	(26.0)	49,323	(41.4)	61,361	(51.5)
**Relative Mediterranean diet (51) [*n* (%)]**																
Low	18,300	(15)	33,811	(28)	39,834	(33)	43,173	(36)	10,633	(8.9)	36,325	(30.5)	45,808	(38.4)	42,352	(35.5)
Medium	52,658	(44)	56,708	(48)	55,184	(46)	54,614	(46)	51,694	(43.4)	58,211	(48.8)	53,030	(44.5)	56,229	(47.2)
High	48,233	(40)	28,673	(24)	24,175	(20)	21,405	(18)	56,864	(47.7)	24,656	(20.7)	20,355	(17.1)	20,611	(17.3)
FSA-NPS DI Score* (50) [mean (SD)]	5.2	(2.1)	5.9	(1.9)	6.2	(2.0)	6.5	(2.2)	5.1	(2.0)	5.9	(1.9)	6.1	(2.0)	6.7	(2.2)
Energy intake, kcal/d [mean (SD)]	2,030	(611)	2,056	(599)	2,083	(604)	2,116	(650)	2,142	(642)	2,047	(615)	2,028	(595)	2,068	(611)
Alcohol intake, g/d [mean (SD)]	15	(21)	13	(17)	11	(15)	8	(13)	15	(21)	13	(18)	12	(15)	9	(12)
Fiber intake, g/d [mean (SD)]	23	(8)	23	(8)	23	(8)	23	(8)	23	(8)	22	(7)	22	(8)	23	(8)
Total fat intake, g/d [mean (SD)]	77	(27)	80	(29)	81	(30)	82	(31)	82	(28)	79	(29)	79	(29)	81	(30)
Carbohydrate intake, g/d [mean (SD)]	216	(74)	224	(70)	232	(71)	246	(80)	231	(80)	226	(73)	226	(70)	236	(74)
Total proteins, g/d [mean (SD)]	90	(29)	87	(27)	86	(26)	84	(26)	95	(30)	86	(28)	84	(25)	83	(25)
Calcium intake, g/d [mean (SD)]	994	(432)	1,023	(406)	1,014	(397)	950	(405)	998	(443)	1,014	(415)	1,007	(396)	962	(387)
Sodium intake, g/d [mean (SD)]	2,649	(1,017)	2,737	(1,063)	2,725	(1,048)	2,719	(1,032)	2,654	(1,035)	2,584	(998)	2,711	(1,031)	2,881	(1,074)

*A higher FSAm-NPS Dietary Index score reflects an overall lower nutritional quality of consumed foods.

The contribution of UPF intake to overall diet varied substantially within the different countries (Supplementary Table 5 and Supplementary Figures 1A,B). The contribution of UPF intake to overall diet in grams/day varied from 7% (France) to 23% (Norway) and their contribution to overall daily energy intake varied from 16% (Spain and Italy) to 46% (for the Norway).

Supplementary Tables 7A,B present the contributions of the different EPIC food groups to the four Nova categories expressed in g/day and kcal/day using the middle bound scenario. “Tea and coffee” were the highest contributors for Nova group 1 while “Beer and cider” were the main contributors to Nova group 3 and “carbonated/soft/isotonic drinks and diluted syrups” were the highest contributors to Nova group 4 when using the absolute values in g/day. When considering the contributions in kcal/day, “fruits” were the main contributors to Nova group 1, while “Bread, crispbread and rusks” were the main contributors for both Nova groups 3 and 4.

The 3 Nova scenarios (lower bound = lowest degree of processing; middle bound = most likely scenario and upper bound = more processed scenario) performed by the international team (USP and IARC) for the Spanish food list were compared with the coding (most likely scenario) applied in Spain to the Spanish food list as one of the quality controls. This demonstrated good comparability (Spearman correlation for % energy derived from UPF = 0.78) between the codes independently assigned by the two teams for the middle bound/most likely scenario (Table 2). The lower and middle bound scenarios gave very similar results while the associations in the upper bound scenario were lower. The weighted kappa statistics also demonstrated good agreement (kappa ranged between 0.48 and 0.68 depending on sex and region) between the two independently assigned Nova classifications for the Spanish EPIC cohort (Supplementary Table 8).

**TABLE 2:** Correlations between the % of Energy from Ultra-Processed Food (UPF) obtained from the Nova coding performed by the Spanish team versus those obtained via the coding performed by the international team for the Spanish food list using the three different scenarios: lower bound (LB), middle bound (MB) and upper bound (UB) for classifying Nova group 4 (Ultra-processed foods).

		Spearman correlation			Weighted kappa	SE for Kappa	Lower CI	Upper CI	Weighted kappa	SE for Kappa	Lower CI	Upper CI	Weighted kappa	SE for Kappa	Lower CI	Upper CI
					
	*N*	LB	MB	UB	LB				MB				UB			
Total EPIC Spain	41,437	0.77	0.78	0.54	0.58	0.0025	0.57	0.58	0.58	0.0025	0.58	0.59	0.37	0.0025	0.36	0.37
Male	15,629	0.72	0.73	0.45	0.53	0.0040	0.52	0.54	0.53	0.0040	0.53	0.54	0.30	0.0040	0.29	0.31
Female	25,808	0.80	0.80	0.59	0.61	0.0031	0.60	0.61	0.61	0.0031	0.61	0.62	0.40	0.0031	0.40	0.41
Asturias	8,542	0.75	0.75	0.60	0.55	0.0054	0.54	0.56	0.55	0.0054	0.54	0.56	0.42	0.0054	0.41	0.43
Male	3,083	0.71	0.72	0.53	0.51	0.0010	0.51	0.51	0.52	0.0010	0.52	0.52	0.36	0.0010	0.35	0.36
Female	5,459	0.76	0.77	0.64	0.57	0.0013	0.56	0.57	0.57	0.0013	0.56	0.57	0.45	0.0013	0.45	0.45
Granada	7,879	0.84	0.84	0.57	0.65	0.0056	0.64	0.66	0.66	0.0056	0.64	0.67	0.39	0.0056	0.38	0.40
Male	1,796	0.81	0.82	0.51	0.62	0.0007	0.62	0.62	0.62	0.0007	0.62	0.62	0.34	0.0007	0.34	0.34
Female	6,083	0.84	0.85	0.58	0.66	0.0014	0.66	0.66	0.67	0.0014	0.66	0.67	0.40	0.0014	0.40	0.40
Murcia	8,515	0.82	0.83	0.60	0.63	0.0054	0.62	0.64	0.64	0.0054	0.63	0.65	0.41	0.0054	0.40	0.42
Male	2,684	0.78	0.79	0.53	0.59	0.0009	0.59	0.59	0.59	0.0009	0.59	0.60	0.36	0.0009	0.36	0.36
Female	5,831	0.84	0.84	0.63	0.65	0.0013	0.65	0.65	0.65	0.0013	0.65	0.65	0.44	0.0013	0.44	0.44
Navarra	8,084	0.76	0.76	0.53	0.56	0.0056	0.55	0.58	0.57	0.0056	0.56	0.58	0.36	0.0056	0.35	0.37
Male	3,908	0.72	0.72	0.45	0.53	0.0011	0.53	0.53	0.53	0.0011	0.53	0.53	0.30	0.0011	0.30	0.31
Female	4,176	0.77	0.77	0.60	0.58	0.0011	0.58	0.58	0.58	0.0011	0.58	0.58	0.43	0.0011	0.43	0.43
San Sebastian	8,417	0.70	0.71	0.45	0.51	0.0054	0.50	0.52	0.52	0.0054	0.51	0.53	0.30	0.0054	0.28	0.31
Male	4,158	0.66	0.67	0.35	0.47	0.0011	0.47	0.47	0.48	0.0011	0.47	0.48	0.22	0.0011	0.22	0.22
Female	4,259	0.74	0.74	0.55	0.55	0.0011	0.54	0.55	0.55	0.0011	0.55	0.55	0.37	0.0011	0.37	0.37

Associations were investigated between the consumption of the 4 Nova groups and objective biomarkers related to food processing. Associations of industrial ITFA plasma levels (elaidic acid) with intakes of the different Nova groups in g/day, kcal/day, % of g/day and % of kcal/day were investigated in a subset of subjects from the nested case-control studies embedded in EPIC (*N* = 9,460) and are presented in Table 3. The % of grams and energy derived from UPF (Nova group 4) were fair to moderately and statistically significantly positively correlated with ITFA (elaidic acid) plasma levels (Spearman *r* for middle bound scenario = 0.37 and 0.54, respectively), while inverse or lower positive correlations were found with any other Nova group (see Table 3). Nova group 1 (fresh and minimally processed foods) also showed a positive association when considering % grams/day (Spearman *r* for middle bound scenario = 0.17) but an inverse association when considering % kcal/day (Spearman *r* for middle bound scenario =−0.07). Overall, the correlations of the middle bound scenario (the most likely scenario) were most in line with our hypotheses that higher intakes of UPF would lead to higher plasma ITFA levels compared to the lower and upper bound scenario; this suggests better performance of this most likely scenario.

**TABLE 3:** Unadjusted associations of elaidic acid levels in plasma with the daily grams, energy, % grams and % energy intake from the 4 different Nova groups and middle bound scenario (*N* = 9460).

	Pearson correlation		Spearman correlation	
		
Middle bound	*R* (Unadjusted association)	*p*-value (unadjusted)	*R* (Unadjusted association)	*p*-value (unadjusted)
**Expressed in g/day**				
Unprocessed or minimally processed foods –G1	0.20	<0.0001	0.25	<0.0001
Processed culinary ingredients –G2	–0.38	<0.0001	–0.43	<0.0001
Processed foods –G3	–0.35	<0.0001	–0.37	<0.0001
Processed foods –G3 excl. alcohol intake	–0.30	<0.0001	–0.31	<0.0001
Ultra-processed foods –G4	0.37	<0.0001	0.44	<0.0001
Ultra-processed foods –G4 excl. alcohol intake	0.37	<0.0001	0.44	<0.0001
**Expressed in kcal/day**				
Unprocessed or minimally processed foods –G1	–0.11	<0.0001	–0.10	<0.0001
Processed culinary ingredients –G2	–0.43	<0.0001	–0.48	<0.0001
Processed foods –G3	–0.32	<0.0001	–0.32	<0.0001
Processed foods –G3 excl. alcohol intake	–0.25	<0.0001	–0.26	<0.0001
Ultra-processed foods –G4	0.45	<0.0001	0.47	<0.0001
Ultra-processed foods –G4 excl. alcohol intake	0.45	<0.0001	0.48	<0.0001
**Expressed in % of g/day incl. alcohol intake**				
Unprocessed or minimally processed foods –G1	0.18	<0.0001	0.17	<0.0001
Processed culinary ingredients –G2	–0.39	<0.0001	–0.46	<0.0001
Processed foods –G3	–0.40	<0.0001	–0.44	<0.0001
Ultra-processed foods –G4	0.30	<0.0001	0.37	<0.0001
**Expressed in % of kcal/day incl. alcohol intake**				
Unprocessed or minimally processed foods –G1	–0.07	<0.0001	–0.07	<0.0001
Processed culinary ingredients –G2	–0.46	<0.0001	–0.49	<0.0001
Processed foods –G3	–0.34	<0.0001	–0.34	<0.0001
Ultra-processed foods –G4	0.53	<0.0001	0.54	<0.0001
**Expressed in % of g/day excl. alcohol intake**				
Unprocessed or minimally processed foods –G1	0.08	<0.0001	0.07	<0.0001
Processed culinary ingredients –G2	–0.41	<0.0001	–0.47	<0.0001
Processed foods –G3	–0.35	<0.0001	–0.39	<0.0001
Ultra-processed foods –G4	0.28	<0.0001	0.34	<0.0001
**Expressed in % of kcal/day excl. alcohol intake**				
Unprocessed or minimally processed foods –G1	–0.11	<0.0001	–0.11	<0.0001
Processed culinary ingredients –G2	–0.47	<0.0001	–0.51	<0.0001
Processed foods –G3	–0.29	<0.0001	–0.29	<0.0001
Ultra-processed foods –G4	0.52	<0.0001	0.53	<0.0001

Supplementary Table 9 presents the correlations for the 4 different Nova groups for lower, middle and upper bound scenarios adjusted for sex, age, BMI and country.

Associations of urinary methyl syringol sulfate with intakes of the different Nova groups (in g/day, kcal/day, % of g/day and % of kcal/day) were similarly investigated in another subset of subjects, derived from the EPIC calibration study (*N* = 417) and are presented in Table 4. These results also demonstrated fair correlations between the UPF (Nova 4) group and this food processing metabolite while inverse associations for Nova groups 1 and 2 and null for Nova group 3 (except for the Nova group 1 values expressed in grams/day). Associations were again strongest for the middle bound scenario and when using the % kcal/day units.

**TABLE 4:** Unadjusted associations of urinary methylsyringol sulfate with the daily grams, energy, % grams and % energy intake from the 4 different Nova groups and middle bound scenario (*N* = 417).

Middle bound	Pearson correlation		Spearman correlation	
		
	*R* (Unadjusted association)	*p*-value (unadjusted)	*R* (Unadjusted association)	*p*-value (unadjusted)
**Expressed in g/day**				
Unprocessed or minimally processed foods –G1	0.16	0.001	0.22	<0.0001
Processed culinary ingredients –G2	–0.20	0.0001	–0.30	<0.0001
Processed foods –G3	0.13	0.007	0.12	0.01
Processed foods –G3 excluding alcohol intake	–0.07	0.14	–0.04	0.44
Ultra-processed foods –G4	0.35	<0.0001	0.40	<0.0001
Ultra-processed foods –G4 excluding alcohol intake	0.35	<0.0001	0.39	<0.0001
**Expressed in kcal/day**				
Unprocessed or minimally processed foods –G1	–0.24	<0.0001	–0.27	<0.0001
Processed culinary ingredients –G2	–0.30	<0.0001	–0.36	<0.0001
Processed foods –G3	0.06	0.26	0.10	0.03
Processed foods –G3 excluding alcohol intake	0.03	0.55	0.08	0.10
Ultra-processed foods –G4	0.37	<0.0001	0.41	<0.0001
Ultra-processed foods –G4 excluding alcohol intake	0.37	<0.0001	0.40	<0.0001
**Expressed in % g/day including alcohol intake**				
Unprocessed or minimally processed foods –G1	–0.06	0.23	–0.07	0.18
Processed culinary ingredients –G2	–0.37	<0.0001	–0.41	<0.0001
Processed foods –G3	–0.07	0.172	–0.07	0.15
Ultra-processed foods –G4	0.25	<0.0001	0.29	<0.0001
**Expressed in % kcal/day including alcohol intake**				
Unprocessed or minimally processed foods –G1	–0.33	<0.0001	–0.37	<0.0001
Processed culinary ingredients –G2	–0.39	<0.0001	–0.42	<0.0001
Processed foods –G3	0.04	0.36	0.07	0.15
Ultra-processed foods –G4	0.41	<0.0001	0.43	<0.0001
**Expressed in % g/day excluding alcohol intake**				
Unprocessed or minimally processed foods –G1	–0.0003	0.996	–0.02	0.75
Processed culinary ingredients –G2	–0.36	<0.0001	–0.39	<0.0001
Processed foods –G3	–0.26	<0.0001	–0.24	<0.0001
Ultra-processed foods –G4	0.27	<0.0001	0.32	<0.0001
**Expressed in % kcal/day excluding alcohol intake**				
Unprocessed or minimally processed foods –G1	–0.32	<0.0001	–0.36	<0.0001
Processed culinary ingredients –G2	–0.38	<0.0001	–0.41	<0.0001
Processed foods –G3	0.02	0.70	0.05	0.35
Ultra-processed foods –G4	0.42	<0.0001	0.43	<0.0001

Supplementary Table 10 presents the correlations for the four different Nova groups and the lower, middle and upper bound scenarios adjusted for sex, age, BMI and country.

Sensitivity analyses were run for the Nova groups (the three different scenarios and expressed as g/day, kcal/day, % of g/day and % of kcal/day) using partial correlations adjusted for sex, age and BMI which gave very similar results as for the unadjusted analyses, while additionally adjusting for country attenuated the correlations (see Supplementary Tables 9, 10).

## Discussion

The results from this multicenter European study, demonstrate sociodemographic and geographical differences in the consumption of UPF. Furthermore, the comparison with the objective biomarkers, i.e., plasma ITFA and a urinary methylsyringol metabolite showed fair to moderate correlations with the % energy derived from UPF further supporting that the Nova classification is generally suitable for the evaluation of food according to the degree of processing among European populations. The broad variety of foods included in the UPF (Nova 4) group may partially explain the fair to moderate correlations found in relation to the food processing biomarkers. The higher correlations found when considering energy intakes instead of grams of UPF in relation to the food processing biomarkers may be due to the higher energy content of foods high in trans-fat and smoked meat.

The correlation with the food processing biomarkers was slightly higher for the middle bound scenario than for the lower and upper bound scenarios, which suggests better performance of this most likely scenario. Hence, future analyses investigating disease outcomes in relation to the consumption of UPF using the Nova classification are advised to predominantly use the middle bound scenario.

Adjusting the analyses for sex, age and BMI had overall little impact on the correlations with the food processing biomarkers. However, adjustment for country attenuated the correlations (Supplementary Tables 9, 10). These reduced correlations when adjusting for country could potentially be due to loss in power. In addition, the different number of food items in the questionnaires of the various countries (Supplementary Table 1) may also contribute to this attenuation when adjusting for country (e.g., FFQs with fewer food items and less details may underestimate transfatty acid intakes).

Characterisation of the degree of food processing in EPIC demonstrated differences between countries, with contributions of UPF intake to the overall diet in grams/day varying from 7% (France) to 23% (Norway) and their contributions to overall energy intake varying from 16% (Spain and Italy) to 46% (the Norway). In addition, differences were also found between sociodemographic groups in the consumption of ultra-processed and minimally processed foods. Indeed, participants in the highest fourth of UPF consumption tended to be younger, taller, more often current smokers, more physically active, have a lower level of attained education, have a higher reported intake of energy and lower reported intake of alcohol. These results on the characterisation of the degree of food processing in EPIC are in line with the findings from the NutriNet-Santé Cohort (apart from the result for physical activity, showing higher consumption of UPF among highly active people in EPIC) (16, 52). However, overall the consumption of UPF in EPIC was lower than in other surveys and cohorts while the consumption of minimally processed foods was overall higher in comparison with recent studies from the UK and France for instance (13, 16, 19, 52, 53) and a comparison across the nineteen countries (53). This difference may potentially be due to the fact that the baseline data in EPIC, used in this study, have been collected in the late 1990s, when dietary patterns in many European countries may still have been predominantly based on fresh food products and, to a lower extent, UPF. It should also be noted that the characteristics investigated in Table 1 should be interpreted with caution as factors such as age, sex, country, etc. may also play a role in some of these findings (e.g., higher consumers of UPF may potentially be more active because they are younger).

Our study is the largest ongoing multicentre cohort study conducted in Europe with a large battery of detailed participant information. Except for a study investigating associations between UPF consumption and urinary concentrations of phthalates and bisphenol (two biomarkers for exposure to packaging materials) in a nationally representative sample of the US population (54), and two studies investigating metabolic biomarkers of diet quality and UPF in European children (55, 56), according to our knowledge this is the first study that investigates the validity of the Nova classification by comparison with food processing biomarkers in blood and urine. Strengths are the wide range of exposures covered by the 9 different European countries, the use of the standardized methodology and procedures to collect participant information, the use of validated FFQs and standardized methods for classifying food items regarding processing with nutritional experts. Still some limitations need to be acknowledged. Dietary questionnaires provide less detailed information on food processing than data from 24 h recalls or food diaries; though the EPIC questionnaires are very detailed, delivering a food list of more than 11,000 food items after decomposing recipes into ingredients. We acknowledge that differences in dietary questionnaires between the EPIC centres could potentially affect the Nova food processing categories. However, a standardized data coding protocol was employed across the EPIC centres, which included disaggregation of homemade recipes into ingredients (commercial recipes were not decomposed into ingredients). This disaggregation into ingredients was essential to correctly assess the consumption of culinary ingredients (Nova group 2); however, this may have led to an overrepresentation of foods classified as Nova group 1 and 2 items instead of group 3 and group 4 items as some of these ingredients may have been processed (e.g., canned) while this level of detail is not available in dietary questionnaires. In addition, recipes that were made at home in the 1990s may nowadays be industrially processed. All the data used in these methodological analyses, namely the dietary intakes as well as the food processing biomarkers were collected at baseline. It should be considered that for some products, the food processing techniques might have changed over time (e.g., recent trans-fat ban in several countries) (57). To consider such potential changes over time in future etiological analyses, three different scenarios were created, namely lower, middle and upper bound scenarios. Although the middle bound scenario compares best with the objective ITFA measurements also taken at baseline, the lower and upper bound scenarios can still be used in sensitivity analyses to explore the potential impact of further industrialisation of food products and of changes in consumer habits to convenience foods over time (considering that the food environment may have changed over time compared to baseline). Still, the lack of dietary follow-up data could be considered as a potential limitation for etiological analyses. Finally it should also be noted that the objective biomarkers for food processing conveniently available and used in this study (elaidic acid and a syringol metabolite) are only reflecting part of the industrial processes. Therefore, the use of extra food processing biomarkers is recommended for future analyses when resources for additional measurements (e.g., additives metabolites, furan compounds, pyrrole compounds and pyrazine compounds) are available. It should also be noted that dietary biomarkers are also prone to within person variability (depending on people’s recent dietary intakes and the time of specimen collection), while unfortunately only one single biospecimen collection was available for all subjects. In addition, consumption of naturally smoked foods classified as processed foods may also contribute to the measurement of syringol metabolites in addition to the consumption of UPF.

In conclusion, our analyses on the characterisation of the degree of food processing among various participating countries in the EPIC cohort demonstrated a pronounced gradient between and within countries, with higher consumption of UPF in individuals who were younger, taller, current smokers, more physically active, and with lower level of attained education, higher reported intake of energy and lower reported intake of alcohol. In addition, the comparison with the objective biomarkers, i.e., plasma ITFA and urinary syringol metabolites showed fair to moderate correlations with the % energy derived from UPF, supporting that the Nova classification is generally suitable for the evaluation of UPF among European populations.

## Data availability statement

The data analyzed in this study is subject to the following licenses/restrictions: EPIC data and biospecimens are available for investigators who seek to answer important questions on health and disease in the context of research projects that are consistent with the legal and ethical standard practices of IARC/WHO and the EPIC centres. The primary responsibility for accessing the data, including the NOVA categories obtained in the frame of the present publication, belongs to the EPIC centres that provided them. The use of a random sample of anonymised data from the EPIC study can be requested by contacting epic@iarc.fr. The request will then be passed to members of the EPIC Steering Committee for deliberation. Requests to access these datasets should be directed to epic@iarc.fr.

## Ethics statement

The studies involving human participants were reviewed and approved by IARC Ethics Committee (IEC). The patients/participants provided their written informed consent to participate in this study.

## Author contributions

IH: conceptualization and writing – original draft. IH, CB, CC, and GN: data curation. NK, RW, CB, and CC: formal analysis. IH and CM: funding acquisition. IH, FR, GN, CC, NK, RW, CB, CM, and RL: investigation. MG, CM, and RL: supervision. All authors: writing – review and editing and approved the submitted version.

## Footnotes

1
https://fr.openfoodfacts.org/
