# Corrigendum: Characterization of the degree of food processing in the European Prospective Investigation into Cancer and Nutrition: application of the Nova classification and validation using selected biomarkers of food processing

**DOI:** 10.3389/fnut.2023.1207555

**Published:** 2023-05-16

**Authors:** Inge Huybrechts, Fernanda Rauber, Geneviève Nicolas, Corinne Casagrande, Nathalie Kliemann, Roland Wedekind, Carine Biessy, Augustin Scalbert, Mathilde Touvier, Krasimira Aleksandrova, Paula Jakszyn, Guri Skeie, Rashmita Bajracharya, Jolanda M. A. Boer, Yan Borné, Veronique Chajes, Christina C. Dahm, Lucia Dansero, Marcela Guevara, Alicia K. Heath, Daniel B. Ibsen, Keren Papier, Verena Katzke, Cecilie Kyrø, Giovanna Masala, Esther Molina-Montes, Oliver J. K. Robinson, Carmen Santiuste de Pablos, Matthias B. Schulze, Vittorio Simeon, Emily Sonestedt, Anne Tjønneland, Rosario Tumino, Yvonne T. van der Schouw, W. M. Monique Verschuren, Beatrice Vozar, Anna Winkvist, Marc J. Gunter, Carlos A. Monteiro, Christopher Millett, Renata Bertazzi Levy

**Affiliations:** ^1^Nutrition and Metabolism Branch, International Agency for Research on Cancer, Lyon, France; ^2^Department of Preventive Medicine, School of Medicine, University of São Paulo, São Paulo, Brazil; ^3^Center for Epidemiological Research in Nutrition and Health, University of São Paulo, São Paulo, Brazil; ^4^Sorbonne Paris Nord University, INSERM U1153, INRAE U1125, CNAM, Nutritional Epidemiology Research Team (EREN), Epidemiology and Statistics Research Center, University Paris Cité (CRESS), Paris, France; ^5^Department of Epidemiological Methods and Etiological Research, Leibniz Institute for Prevention Research and Epidemiology (BIPS), Bremen, Germany; ^6^Human and Health Sciences, University of Bremen, Bremen, Germany; ^7^Unit of Nutrition and Cancer, Cancer Epidemiology Research Programme, Catalan Institute of Oncology (ICO-IDIBELL), Barcelona, Spain; ^8^Blanquerna School of Health Sciences, Ramon Llull University, Barcelona, Spain; ^9^Department of Community Medicine, UiT the Arctic University of Norway, Tromsø, Norway; ^10^German Cancer Research Center (DKFZ), Heidelberg, Germany; ^11^Centre for Nutrition, Prevention and Health Services, National Institute for Public Health and the Environment (RIVM), Bilthoven, Netherlands; ^12^Department of Clinical Sciences Malmö, Faculty of Medicine, Nutritional Epidemiology, Lund University, Lund, Sweden; ^13^Department of Public Health, Aarhus University, Aarhus, Denmark; ^14^Department of Clinical and Biological Sciences, Centre for Biostatistics, Epidemiology, and Public Health (C-BEPH), University of Turin, Turin, Italy; ^15^Instituto de Salud Pública de Navarra, Pamplona, Spain; ^16^Centro de Investigación Biomédica en Red de Epidemiología y Salud Pública (CIBERESP), Madrid, Spain; ^17^Navarra Institute for Health Research (IdiSNA), Pamplona, Spain; ^18^Department of Epidemiology and Biostatistics, School of Public Health, Imperial College London, London, United Kingdom; ^19^Steno Diabetes Center Aarhus, Aarhus, Denmark; ^20^MRC Epidemiology Unit, University of Cambridge School of Clinical Medicine, Cambridge, United Kingdom; ^21^Department of Nutrition, Exercise and Sports, University of Copenhagen, Frederiksberg, Denmark; ^22^Cancer Epidemiology Unit, Nuffield Department of Population Health, University of Oxford, Oxford, United Kingdom; ^23^Danish Cancer Society Research Center, Danish Cancer Society, Copenhagen, Denmark; ^24^Clinical Epidemiology Unit, Institute for Cancer Research, Prevention and Clinical Network (ISPRO), Florence, Italy; ^25^Department of Nutrition and Food Science, Campus of Cartuja, University of Granada, Granada, Spain; ^26^CIBER of Epidemiology and Public Health (CIBERESP), Madrid, Spain; ^27^Instituto de Investigación Biosanitaria ibs.GRANADA, Granada, Spain; ^28^Biomedical Research Centre, Institute of Nutrition and Food Technology (INYTA) “José Mataix”, University of Granada, Granada, Spain; ^29^MRC Centre for Environment and Health, School of Public Health, Imperial College London, London, United Kingdom; ^30^Department of Epidemiology, Murcia Regional Health Council, IMIB-Arrixaca, Murcia, Spain; ^31^Department of Molecular Epidemiology, German Institute of Human Nutrition Potsdam-Rehbruecke, Nuthetal, Germany; ^32^Institute of Nutritional Science, University of Potsdam, Potsdam, Germany; ^33^Dipartimento di Salute Mentale e Fisica e Medicina Preventiva, Vanvitelli University, Naples, Italy; ^34^Hyblean Association for Epidemiological Research, AIRE ONLUS, Ragusa, Italy; ^35^Julius Center for Health Sciences and Primary Care, University Medical Center Utrecht, Utrecht University, Utrecht, Netherlands; ^36^Sustainable Health, Department Public Health and Clinical Medicine, Umeå University, Umeå, Sweden; ^37^Department of Internal Medicine and Clinical Nutrition, Sahlgrenska Academy, University of Gothenburg, Gothenburg, Sweden; ^38^Department of Nutrition, School of Public Health, University of São Paulo, São Paulo, Brazil; ^39^Public Health Policy Evaluation Unit, School of Public Health, Imperial College London, London, United Kingdom

**Keywords:** food processing, Nova, EPIC, biomarkers, elaidic acid, syringol

In the published article, there was an error in Table 4 as published. A column subheading was inserted in the middle of the table by mistake. Furthermore, two values in the “R (Unadjusted association)” column were not rounded. This subheading was removed. The corrected Table 4 appears below.

**Table 4:** Unadjusted associations of urinary methylsyringol sulfate with the daily grams, energy, % grams and % energy intake from the 4 different Nova groups and middle bound scenario (*N* = 417).

**Middle bound**	**Pearson correlation**		**Spearman correlation**	
	***R*** **(Unadjusted association)**	***p*** **-value (unadjusted)**	***R*** **(Unadjusted association)**	***p*** **-value (unadjusted)**
**Expressed in g/day**				
Unprocessed or minimally processed foods—G1	0.16	0.001	0.22	<0.0001
Processed culinary ingredients—G2	−0.20	0.0001	−0.30	<0.0001
Processed foods—G3	0.13	0.007	0.12	0.01
Processed foods—G3 excluding alcohol intake	−0.07	0.14	−0.04	0.44
Ultra-processed foods—G4	0.35	<0.0001	0.40	<0.0001
Ultra-processed foods—G4 excluding alcohol intake	0.35	<0.0001	0.39	<0.0001
**Expressed in kcal/day**				
Unprocessed or minimally processed foods—G1	−0.24	<0.0001	−0.27	<0.0001
Processed culinary ingredients—G2	−0.30	<0.0001	−0.36	<0.0001
Processed foods—G3	0.06	0.26	0.10	0.03
Processed foods—G3 excluding alcohol intake	0.03	0.55	0.08	0.10
Ultra-processed foods—G4	0.37	<0.0001	0.41	<0.0001
Ultra-processed foods—G4 excluding alcohol intake	0.37	<0.0001	0.40	<0.0001
**Expressed in % g/day including alcohol intake**				
Unprocessed or minimally processed foods—G1	−0.06	0.23	−0.07	0.18
Processed culinary ingredients—G2	−0.37	<0.0001	−0.41	<0.0001
Processed foods—G3	−0.07	0.172	−0.07	0.15
Ultra-processed foods—G4	0.25	<0.0001	0.29	<0.0001
**Expressed in % kcal/day including alcohol intake**				
Unprocessed or minimally processed foods—G1	−0.33	<0.0001	−0.37	<0.0001
Processed culinary ingredients—G2	−0.39	<0.0001	−0.42	<0.0001
Processed foods—G3	0.04	0.36	0.07	0.15
Ultra-processed foods—G4	0.41	<0.0001	0.43	<0.0001
**Expressed in % g/day excluding alcohol intake**				
Unprocessed or minimally processed foods—G1	−0.0003	0.996	−0.02	0.75
Processed culinary ingredients—G2	−0.36	<0.0001	−0.39	<0.0001
Processed foods—G3	−0.26	<0.0001	−0.24	<0.0001
Ultra-processed foods—G4	0.27	<0.0001	0.32	<0.0001
**Expressed in % kcal/day excluding alcohol intake**				
Unprocessed or minimally processed foods—G1	−0.32	<0.0001	−0.36	<0.0001
Processed culinary ingredients—G2	−0.38	<0.0001	−0.41	<0.0001
Processed foods—G3	0.02	0.70	0.05	0.35
Ultra-processed foods—G4	0.42	<0.0001	0.43	<0.0001

The authors apologize for this error and state that this does not change the scientific conclusions of the article in any way. The original article has been updated.

