# Supplementary material for: Characterization of the degree of food processing in the European Prospective Investigation into Cancer and Nutrition: application of the Nova classification and validation using selected biomarkers of food processing
Source: Front Nutr. 2022 Dec 16;9:1035580. doi: 10.3389/fnut.2022.1035580 (PMC9800919; doi:10.3389/fnut.2022.1035580)
Supplement: Supplementary file 1 [file Data_Sheet_1.docx]

Supplementary Material

# Overview of Supplementary material

**Supplementary Table 1: Dietary assessment methods used to estimate dietary intakes across countries/centres in the EPIC cohort study (centralised database) and the number of food items in the diet questionnaire food list per country and in total.**

**Supplementary Table 2: NOVA groups and subgroups used for the coding of the foods in the EPIC food list.**

**Supplementary Table 3: Examples of the classification of foods and ingredients according to the NOVA classification, using the following three scenarios: the lower bound (LB) scenario reflecting the lowest degree of processing, the middle bound scenario (MB) reflecting the most likely scenario, and the more processed or upper bound (UB) scenario.**

**Supplementary Table 4: Distributions of the different NOVA categories, group G1 to G4, using the three different scenarios (Lower, Middle and Upper Bound scenario) for the total EPIC cohort (N= 476,768; distributions per country are provided in Supplementary Table 5). Results derived from the NOVA classification in which alcoholic drinks were excluded have been shaded in grey.**

**Supplementary Table 5: Distributions of the different NOVA groups (G1 to G4) by country using the three different scenarios (Lower, Middle and Upper bound).**

**Supplementary Table 6a: Characteristics of the study population by sex-specific quartiles of relative intake of NOVA group 1 - Unprocessed or minimally processed foods (% g/day and % kcal/day including alcohol)**

**Supplementary Table 6b: Characteristics of the study population by sex-specific quartiles of relative intake of NOVA group 2 - Processed culinary ingredients (% g/day and % kcal/day including alcohol)**

**Supplementary Table 6c: Characteristics of the study population by sex-specific quartiles of relative intake of NOVA group 3 - Processed foods (% g/day and % kcal/day including alcohol)**

**Supplementary Table 7a: The contributions of the different EPIC food groups to the four NOVA categories expressed in kcal/day for the middle bound (MB) scenario.**

**Supplementary Table 7b: The contributions of the different EPIC food groups to the four NOVA categories expressed in g/day for the middle bound (MB) scenario**

**Supplementary Table 8: Correlations for the NOVA group 4 data (expressed in absolute and in % kcal/day) coded by the Spanish team versus those obtained via the coding performed by the international team (IARC and USP) for the Spanish food list using the three different scenarios.**

**Supplementary Table 9: Partial Correlations of elaidic acid with intakes of the 4 different NOVA groups and for the lower, middle and upper bound scenarios expressed as g/day, kcal/day, %g/day and %kcal/day including or excluding alcohol. Partial correlations are adjusted for sex, age, BMI and country (N=9,460)**

**Supplementary Table 10: Partial correlations of urinary methyl syringol sulfate with intakes of the 4 different NOVA and for the lower, middle and upper bound scenarios expressed as g/day, kcal/day, % of g/day and % of kcal/day including or excluding alcohol. Partial correlations are adjusted for sex, age, BMI and country (N=417)**

# Supplementary Figure 1a: Distributions of the different NOVA categories by Country, group (G) 1 to G 4 using the three different scenarios (Lower, Middle and Upper bound) expressed in % g/day.

**Supplementary Figure 1b: Distributions of the different NOVA categories by Country, group (G) 1 to G 4 using the three different scenarios (Lower, Middle and Upper bound) expressed in % kcal/day.**

# Supplementary Tables

**Supplementary table 1:** Dietary assessment methods used to estimate dietary intakes across countries/centres in the EPIC cohort study (centralised database) and the number of food items in the diet questionnaire food list per country and in total.

| **Country** | **Centres (where methods differed) (**Riboli 2002 ([1](#_ENREF_1))**)** | **Dietary assessment tool** | **Mode of administration** | **Number of food items in questionnaire** | **Comments** | **References** | **Number of items in final database (after cleaning)** | |
| --- | --- | --- | --- | --- | --- | --- | --- | --- |
|  |  |  |  |  |  |  | **Foods & recipes** | **Foods & ingredients*** |
| **France** |  | Diet history questionnaire | Self | 238 | Structured by French meal pattern, in two steps: qualitative and quantitative information. Photo book. | Van Liere 1997 ([2](#_ENREF_2)),  Kesse 2001 ([3](#_ENREF_3)) | 210 | 236 |
| **Italy** | Florence, Turin, Varese (North-Central Italy) | Semi-quantitative FFQ | Self | 188 | 248 questions, including on seasoning and food preparation. 17 sets of pictures for portions. | Pala 2003 ([4](#_ENREF_4)) (Sieri 2002 ([5](#_ENREF_5)), Sieri 2004 ([6](#_ENREF_6)), Pisani 1997 ([7](#_ENREF_7))) | 233 | 296 |
|  | Ragusa | Semi-quantitative FFQ | Face-to-face | 217 | 438 questions, including on cooking methods and type of fat used for cooking. 15 sets of pictures. | Pala 2003 ([4](#_ENREF_4)) | 265 | 407 |
|  | Naples | Semi-quantitative FFQ | Face-to-face | 140 | 154 questions, including on cooking methods and type of fat used for cooking. Standard units or possibility to tell smaller or bigger. | Pala 2003 ([4](#_ENREF_4)) | 158 | 177 |
| **Spain** |  | Diet history questionnaire | Face-to-face | Open. Starting list of 600 commonly consumed foods and recipes from each region to facilitate reporting. | Structured by meals. Weekly frequency of consumption reported for each food consumed at least twice a month, taking into account the seasonal consumption. Preparation mode and amount consumed. 35 photograph series, natural units and household measures. | (EPIC group of Spain 1997 ([8](#_ENREF_8)), Agudo 1999 ([9](#_ENREF_9))) | 721 | 2,151 (7,345) |
| **United Kingdom** |  | FFQ | Self | 130 | Brand name and type of most often used breakfast cereals. Questions on fat most often used for frying and for baking and if visible fat usually eaten on meat. Standard portions except for milk specified to the nearest quarter of a pint/day. | Bingham 2001 ([10](#_ENREF_10)), Davey 2003 ([11](#_ENREF_11)) | 170 | 210 |
| **The Netherlands** |  | Semi-quantitative FFQ | Self | 79 | Additional questions on preparation and additions to reach 178 food items. Picture booklet for 21 foods. | Ocke 1997 ([12](#_ENREF_12)) | 213 | 244 |
| **Greece** |  | Semi-quantitative FFQ | Face-to-face | 190 | For 20 items, separate questions concerning the seasonal differences in consumption.Picture booklet. | Katsouyanni 1997 ([13](#_ENREF_13)), Gnardellis 1995 ([14](#_ENREF_14)) | 268 | 807 |
| **Germany** |  | FFQ | Self | 146 | Questions about specific food items, such as the frequency of sauce consumption, the fat content of several food items, and seasonal consumption of fruit and vegetables to reach 252 items. Final check with main food consumption patterns. With photos. | Kroke 1999 ([15](#_ENREF_15))(Potsdam) | 252 | Heidelberg: 559  Potsdam: 526 |
| **Sweden** | Umeå | FFQ | Self | 84 | Average portion of (1) potato/pasta/rice, (2) vegetables and (3) meat/ground meat/sausages assessed by comparison with four colour photos illustrating four plates with increasing portion sizes of potato, vegetables and meat. For the other food items, standard portion size value. | Johansson 2002 ([16](#_ENREF_16)) | 98 | 146 |
|  | Malmö | FFQ +  7-day Menu book for hot meals +  12 cold beverages | Self | 168 + Open | Both checked face-to-face and manually entered in software Kostsvar containing ca 1,600 basic foods. Picture booklet | Wirfält 2000 ([17](#_ENREF_17)) | 2,059 | 5,024  (18,631) |
| **Denmark** |  | Semi-quantitative FFQ | Self | 192 | Frequency and semi-quantity of food consumption, type of fat used on bread/in cooking, doneness of meat, use of dietary supplements Standardized recipes and portion sizes. Software FoodCalc. | Tjønneland 2007 ([18](#_ENREF_18)) | 457 | 904  (5,922) |
| **Norway** |  | FFQ | Self | 85 | Questions on cooking methods, on cod liver oil use, on supplements and 3 alcoholic beverages. | Brustad 2004 ([19](#_ENREF_19)), Hjartåker 2007 ([20](#_ENREF_20)) | 88 | 171 |
| **Total EPIC cohort** | | | | | | | **5,444** | **11,858  (31,898)** |

* **Additional items in complete list**

**Supplementary table 2:** NOVA groups and subgroups used for the coding of the foods in the EPIC food list.

| **NOVA group 1** | **Unprocessed or minimally processed foods** |
| --- | --- |
|  | *Definition: Unprocessed foods are edible parts of plants (fruits, seeds, leaves, stems, roots, tubers) or of animals (muscle, offal, eggs, milk), and also fungi, algae and water, after separation from nature. Minimally processed foods are natural foods that were altered by industrial processes such as removal of inedible or unwanted parts, drying, crushing, grinding, fractioning, filtering, roasting, boiling, pasteurisation, refrigeration, freezing, placing in containers, vacuum packaging, or non-alcoholic fermentation. None of these processes adds sugar, oils or fats or salt or other ingredient of exclusive industrial use to the original food. It also includes foods made up from two or more items in this group, such as dried mixed fruits, granola made from cereals, nuts and dried fruits with no added sugar, honey or oil; pasta, couscous and polenta made with flours, flakes or grits and water;* |
| **Subgroups** | **Label** |
| 101 | Water |
| 102 | Fruit |
| 103 | Milk and plain yoghurt |
| 104 | Cereal, grains and flour made from these foods |
| 105 | Potatoes |
| 106 | Pasta |
| 107 | Beans, lentils and chickpeas |
| 108 | Vegetables |
| 109 | Nuts and Seeds |
| 110 | Eggs |
| 111 | Poultry |
| 112 | Red meat |
| 113 | Fish |
| 114 | Sea food |
| 115 | Fungi |
| 116 | Coffee/tea |
| 117 | Fruit juice fresh and smoothies |
| **Subgroups** | **Label** |
| 118 | Fruit juice UHT or pasteurised |
| 119 | Desserts - homemade |
| 120 | Soup (not commercial) |
| 121 | Sauce homemade, sweet or savoury |
| 122 | Dishes - homemade |
| 123 | Homemade broth |
| **NOVA group 2** | **Processed culinary ingredients** |
|  | *Definition: Processed culinary ingredients are substances obtained directly from group 1 foods or from nature by industrial processes such as pressing, refining, grinding and milling. Examples: vegetable oils crushed from seeds; butter and lard obtained from milk and pork; sugar and molasses obtained from cane or beet; honey extracted from combs and syrup from maple trees; starches extracted from corn and other plants, and salt mined or from seawater* |
| **Subgroups** | **Label** |
| 201 | Table sugar |
| 202 | Plant oil |
| 203 | Animal fats |
| 204 | Other processed culinary ingredients |
| 205 | Salt table |

| **NOVA group 3** | **Processed foods** |
| --- | --- |
|  | *These are relatively simple industrial products made by adding salt, oil, sugar or other group 2 ingredients to group 1 foods. Most processed foods have two or three ingredients. Industrial processes include various preservation or cooking methods and non-alcoholic fermentation. Examples: canned or bottled vegetables and legumes in brine; salted or sugared nuts and seeds; salted, cured, or smoked meats and fish; canned fish; fruits in syrup; freshly made unpackaged breads and cheeses.* |
| **Subgroups** | **Label** |
| 301 | Cheese |
| 302 | Salted, smoked or canned meat, without additives |
| 303 | Salted, smoked or canned fish |
| 304 | Processed bread |
| 305 | Vegetables and other plant foods preserved |
| 306 | Legumes preserved |
| 307 | Fruit preserved |
| 308 | Nuts salted and nut spreads |
| 309 | Beer and Wine |
| 310 | Condensed milk, yogurt plain sweetened, |
| 311 | Bread crumbs |
| 312 | Meringue, non-ultra-processed bakeries |
| 313 | Sauce homemade, sweet or savoury |

| **NOVA group 4** | **Ultra-processed foods (UPF)** |
| --- | --- |
|  | *Formulations of several ingredients that, besides sugar, oil or salt, include protein isolates, modified oils or other food substances of no culinary use, and flavours, colours, and other additives designed to make the product intensely attractive.* |
|  | Ingredients characteristic of ultra-processed foods can be divided into food substances of no (or rare) culinary use and classes of additives whose function is to make the final product palatable or often hyper-palatable. |
|  | - *Food substances only found in UPF include varieties of sugars (fructose, high fructose corn syrup, ‘fruit juice concentrates’, invert sugar, maltodextrin, dextrose, lactose), modified oils (hydrogenated or interesterified oils), and protein sources (hydrolysed proteins, soy protein isolate, gluten, casein, whey protein), and ‘mechanically separated meat’.* - *Additives only found in UPF include flavours, flavour enhancers, colours, emulsifiers, emulsifying salts, sweeteners, thickeners, and anti-foaming, bulking, carbonating, foaming, gelling, and glazing agents. These functional classes of additives disguise undesirable sensory properties created by ingredients, processes or packaging used in the manufacture of UPF, or else give the final product sensory properties especially attractive to see, taste, smell, and/or touch.* |
| **Subgroups** | **Label** |
| 401 | Ultra-processed breads |
| 402 | Pastries, buns, and cakes |
| 403 | Biscuits |
| 404 | Breakfast cereals |
| 405 | Ice cream, ice pops and frozen yogurts |
| 406 | Industrial desserts |
| 407 | Packaged salty snacks |
| 408 | Potato products |
| 409 | Pizza and focaccia (dough) |
| 410 | Pasta (filled) |
| 411 | Instant and canned soups |
| 412 | Dairy substitute products |
| 413 | Processed cheese |
| 414 | Sauces, dressing and gravies - also in powder/dehydrated form/condensed form |
| 415 | Vegetable spread and products |
| **Subgroups** | **Label** |
| 417 | Soft drinks |
| 418 | Dairy desserts and drinks (Ultra Processed versions) |
| 419 | Fruit drinks, iced tea and other sweetened beverages |
| 420 | Beverages dry weight |
| 421 | Alcoholic distilled drinks and other alcoholic drinks |
| 422 | Artificial sweeteners |
| 423 | Sweet snacks |
| 424 | Processed meat (beef, pork and fish) |
| 425 | Meat alternatives |
| 426 | Nutrition powders and drinks |
| 427 | Margarine |
| 428 | Ready meal |
| 429 | Alcohol-free versions of alcoholic beverages |
| 430 | Vegetables and legumes in ultra-processed medium |
| **NOVA group 5** | **Supplements** |
| **Subgroups** | **Label** |
| 501 | Supplements |

**Supplementary Table 3:** Examples of the classification of foods and ingredients according to the NOVA classification, using the following three scenarios: the lower bound (LB) scenario reflecting the lowest degree of processing, the middle bound scenario (MB) reflecting the most likely scenario, and the more processed or upper bound (UB) scenario.

| **Item name** | **Food/ingredient in DQ** | **Lower bound** | **Middle bound** | **Upper bound** |
| --- | --- | --- | --- | --- |
| **Risotto** | Food | Treated as a homemade recipe so decomposed | Treated as a homemade recipe so decomposed | NOVA G4 – readymade commercial |
| **Legumes** | Food | NOVA G1 - dried | NOVA G1 – dried | NOVA G3 – canned |
| **Spring roll, deep dried in the Netherlands** | Food | NOVA G4 - commercial | NOVA G4 - commercial | NOVA G4 - commercial |
| **Bread in France, Italy** | Food | NOVA G3 - bakery | NOVA G3 - bakery | NOVA G4 – commercial |
| **Bread in the United Kingdom** | Food | NOVA G3 - bakery | NOVA G4 – commercial | NOVA G4 – commercial |
| **Cooked tomato** | Ingredient of pizza in Italy | NOVA G1 - fresh | NOVA G1 - fresh | NOVA G4 – commercial pizza |
| **Canned mushroom** | Ingredient of pizza in Sweden | NOVA G3 - canned | NOVA G4 – commercial pizza | NOVA G4 – commercial pizza |

**Supplementary Table 4:** Distributions of the different NOVA categories, group G1 to G4, using the three different scenarios (Lower, Middle and Upper Bound scenario) for the total EPIC cohort (N= 476,768; distributions per country are provided in Supplementary Table 5). Results derived from the NOVA classification in which alcoholic drinks were excluded have been shaded in grey.

| **Label** | **Mean** | **Std** | **Min** | **P25** | **P50** | **P75** | **Max** |
| --- | --- | --- | --- | --- | --- | --- | --- |
| **Grams/day** |  |  |  |  |  |  |  |
| Unprocessed or minimally processed foods -G1 [LB] | **2017.7** | 832.8 | 5.4 | 1,389.6 | 1,913.9 | 2,512.3 | 16,347.4 |
| Unprocessed or minimally processed foods -G1 [MB] | **1970.7** | 833.2 | 5.4 | 1,340.6 | 1,870.6 | 2,466.8 | 16,347.4 |
| Unprocessed or minimally processed foods -G1 [UB] | **1890.7** | 822.5 | 5.4 | 1,267.2 | 1,791.6 | 2,377.4 | 16,033.0 |
| Processed culinary ingredients -G2 [LB] | **32.5** | 23.6 | 0 | 14.0 | 27.0 | 45.6 | 500.5 |
| Processed culinary ingredients -G2 [MB] | **28.7** | 23.4 | 0 | 9.9 | 22.9 | 41.7 | 497.6 |
| Processed culinary ingredients -G2 [UB] | **26.4** | 23.3 | 0 | 7.4 | 20.5 | 39.5 | 493.8 |
| Processed foods -G3 [LB] | **369.2** | 302.4 | 0 | 189.7 | 288.3 | 446.7 | 7598.9 |
| Processed foods -G3 [MB] | **354.7** | 305.6 | 0 | 163.6 | 272.3 | 440.7 | 7622.9 |
| Processed foods -G3 [UB] | **277.3** | 283.6 | 0 | 113.9 | 193.4 | 337.9 | 7477.2 |
| Processed foods -G3 excluding alcohol [LB] | **206.0** | 101.7 | 0 | 133.8 | 191.7 | 263.0 | 1441.5 |
| Processed foods -G3 excluding alcohol [MB] | **191.6** | 122.8 | 0 | 99.0 | 164.6 | 257.0 | 1581.4 |
| Processed foods -G3 excluding alcohol [UB] | **114.2** | 68.8 | 0 | 66.6 | 101.4 | 147.1 | 1388.4 |
| Ultra-processed foods -G4 [LB] | **298.2** | 240.1 | 0 | 143.7 | 234.8 | 375.0 | 4754.1 |
| Ultra-processed foods -G4 [MB] | **363.5** | 263.4 | 0 | 186.9 | 309.0 | 465.5 | 4976.4 |
| Ultra-processed foods -G4 [UB] | **523.2** | 270.1 | 0 | 345.4 | 470.8 | 634.3 | 5281.1 |
| Ultra-processed foods -G4 excluding alcohol [LB] | **289.7** | 239.0 | 0 | 136.5 | 225.7 | 364.5 | 4754.1 |
| Ultra-processed foods -G4 excluding alcohol [MB] | **355.0** | 262.7 | 0 | 178.6 | 299.6 | 456.2 | 4975.7 |
| Ultra-processed foods -G4 excluding alcohol [UB] | **514.7** | 269.2 | 0 | 337.6 | 461.8 | 624.6 | 5281.1 |
| **Kcal/day** |  |  |  |  |  |  |  |
| Energy: Unprocessed or minimally processed foods -G1 [LB] | **799.6** | 281.4 | 5.1 | 601.0 | 766.9 | 959.3 | 4,052.2 |
| Energy: Unprocessed or minimally processed foods -G1 [MB] | **751.5** | 271.3 | 5.1 | 559.7 | 719.5 | 904.5 | 4,019.9 |
| Energy: Unprocessed or minimally processed foods -G1 [UB] | **677.8** | 250.6 | 5.1 | 501.3 | 648.7 | 818.1 | 3,991.1 |
| Energy: Processed culinary ingredients -G2 [LB] | **174.6** | 142.7 | 0 | 62.1 | 135.4 | 252.3 | 2042.0 |
| Energy: Processed culinary ingredients -G2 [MB] | **159.2** | 144.5 | 0 | 42.7 | 118.2 | 239.0 | 2041.9 |
| Energy: Processed culinary ingredients -G2 [UB] | **151.6** | 143.7 | 0 | 34.7 | 109.9 | 231.2 | 2035.5 |
| Energy: Processed foods -G3 [LB] | **612.0** | 315.4 | 0 | 386.2 | 562.4 | 779.2 | 4181.9 |
| Energy: Processed foods -G3 [MB] | **538.7** | 334.8 | 0 | 284.8 | 472.9 | 719.4 | 4203.1 |
| Energy: Processed foods -G3 [UB] | **322.5** | 214.7 | 0 | 174.2 | 272.0 | 414.1 | 3911.9 |
| Energy: Processed foods -G3 excluding alcohol [LB] | **517.0** | 263.2 | 0 | 330.4 | 479.4 | 662.3 | 4176.4 |
| Energy: Processed foods -G3 excluding alcohol [MB] | **443.8** | 291.1 | 0 | 217.3 | 383.5 | 607.5 | 4197.6 |
| Energy: Processed foods -G3 excluding alcohol [UB] | **227.6** | 149.1 | 0 | 127.1 | 196.2 | 288.5 | 3906.4 |
| Energy: Ultra-processed foods -G4 [LB] | **546.4** | 314.2 | 0 | 323.3 | 488.4 | 704.0 | 4182.6 |
| Energy: Ultra-processed foods -G4 [MB] | **683.2** | 392.6 | 0 | 390.8 | 625.1 | 903.9 | 4482.3 |
| Energy: Ultra-processed foods -G4 [UB] | **980.7** | 418.3 | 0 | 685.4 | 921.0 | 1207.7 | 4652.8 |
| Energy: Ultra-processed foods -G4 excluding alcohol [LB] | **529.2** | 309.9 | 0 | 310.0 | 471.6 | 682.7 | 4178.6 |
| Energy: Ultra-processed foods -G4 excluding alcohol [MB] | **666.1** | 389.5 | 0 | 375.5 | 606.7 | 884.2 | 4481.0 |
| Energy: Ultra-processed foods -G4 excluding alcohol [UB] | **963.5** | 415.2 | 0 | 670.4 | 903.5 | 1188.3 | 4651.4 |
| **% grams/day (including alcohol intake)** |  |  |  |  |  |  |  |
| Unprocessed or minimally processed foods -G1 [LB] | **73.6** | 11.6 | 1.5 | 67.2 | 75.7 | 82.1 | 100.0 |
| Unprocessed or minimally processed foods -G1 [MB] | **71.7** | 12.1 | 1.5 | 64.7 | 73.7 | 80.5 | 100.0 |
| Unprocessed or minimally processed foods -G1 [UB] | **68.5** | 12.3 | 1.5 | 61.3 | 70.4 | 77.5 | 100.0 |
| Processed culinary ingredients -G2 [LB] | **1.3** | 1.0 | 0 | 0.5 | 1.0 | 1.8 | 15.9 |
| Processed culinary ingredients -G2 [MB] | **1.2** | 1.0 | 0 | 0.4 | 0.9 | 1.7 | 15.8 |
| Processed culinary ingredients -G2 [UB] | **1.1** | 1.0 | 0 | 0.3 | 0.8 | 1.6 | 15.8 |
| Processed foods -G3 [LB] | **13.8** | 9.1 | 0 | 7.6 | 11.5 | 17.4 | 92.2 |
| Processed foods -G3 [MB] | **13.4** | 9.9 | 0 | 6.5 | 10.5 | 17.3 | 92.4 |
| Processed foods -G3 [UB] | **10.1** | 8.4 | 0 | 4.7 | 7.6 | 12.6 | 90.3 |
| Ultra-processed foods -G4 [LB] | **11.2** | 7.8 | 0 | 5.8 | 9.4 | 14.5 | 89.6 |
| Ultra-processed foods -G4 [MB] | **13.7** | 8.8 | 0 | 7.5 | 11.9 | 17.9 | 90.5 |
| Ultra-processed foods -G4 [UB] | **20.3** | 9.3 | 0 | 13.6 | 18.8 | 25.3 | 91.1 |
| **% Kcal/day (including alcohol intake)** |  |  |  |  |  |  |  |
| Energy: Unprocessed or minimally processed foods -G1 [LB] | **38.3** | 10.6 | 0.8 | 30.9 | 37.9 | 45.1 | 100.0 |
| Energy: Unprocessed or minimally processed foods -G1 [MB] | **36.0** | 10.5 | 0.8 | 28.7 | 35.5 | 42.6 | 100.0 |
| Energy: Unprocessed or minimally processed foods -G1 [UB] | **32.5** | 9.9 | 0.4 | 25.6 | 31.9 | 38.7 | 100.0 |
| Energy: Processed culinary ingredients -G2 [LB] | **8.1** | 5.9 | 0 | 3.2 | 6.7 | 11.8 | 57.4 |
| Energy: Processed culinary ingredients -G2 [MB] | **7.3** | 6.0 | 0 | 2.3 | 5.8 | 11.3 | 57.4 |
| Energy: Processed culinary ingredients -G2 [UB] | **7.0** | 6.0 | 0 | 1.8 | 5.4 | 10.9 | 57.4 |
| Energy: Processed foods -G3 [LB] | **28.2** | 10.6 | 0 | 20.7 | 27.7 | 35.1 | 90.0 |
| Energy: Processed foods -G3 [MB] | **24.6** | 11.8 | 0 | 15.1 | 23.5 | 32.7 | 90.0 |
| Energy: Processed foods -G3 [UB] | **14.8** | 7.9 | 0 | 9.2 | 13.4 | 18.9 | 90.0 |
| Energy: Ultra-processed foods -G4 [LB] | **25.4** | 11.2 | 0 | 17.1 | 24.9 | 32.8 | 83.9 |
| Energy: Ultra-processed foods -G4 [MB] | **32.1** | 14.9 | 0 | 20.1 | 32.3 | 43.1 | 97.5 |
| Energy: Ultra-processed foods -G4 [UB] | **45.7** | 12.6 | 0 | 37.0 | 46.2 | 54.7 | 97.5 |
| **% grams/day (excluding alcohol intake)** |  |  |  |  |  |  |  |
| Unprocessed or minimally processed foods -G1 [LB] | **78.2** | 9.7 | 1.8 | 72.9 | 79.8 | 85.2 | 100.0 |
| Unprocessed or minimally processed foods -G1 [MB] | **76.1** | 10.4 | 1.8 | 70.2 | 77.8 | 83.7 | 100.0 |
| Unprocessed or minimally processed foods -G1 [UB] | **72.7** | 10.8 | 1.8 | 66.4 | 74.3 | 80.6 | 100.0 |
| Processed culinary ingredients -G2 [LB] | **1.4** | 1.1 | 0 | 0.6 | 1.1 | 2.0 | 15.9 |
| Processed culinary ingredients -G2 [MB] | **1.3** | 1.1 | 0 | 0.4 | 0.9 | 1.8 | 15.7 |
| Processed culinary ingredients -G2 [UB] | **1.2** | 1.1 | 0 | 0.3 | 0.8 | 1.7 | 15.7 |
| Processed foods -G3 [LB] | **8.8** | 4.8 | 0 | 5.4 | 7.8 | 11.2 | 58.9 |
| Processed foods -G3 [MB] | **8.4** | 6.2 | 0 | 3.9 | 6.6 | 10.9 | 65.2 |
| Processed foods -G3 [UB] | **4.8** | 3.0 | 0 | 2.7 | 4.1 | 6.1 | 52.7 |
| Ultra-processed foods -G4 [LB] | **11.6** | 8.2 | 0 | 5.9 | 9.7 | 15.1 | 89.8 |
| Ultra-processed foods -G4 [MB] | **14.3** | 9.2 | 0 | 7.6 | 12.4 | 18.8 | 98.1 |
| Ultra-processed foods -G4 [UB] | **21.3** | 9.9 | 0 | 14.2 | 19.7 | 26.8 | 98.1 |
| **% Kcal/day (excluding alcohol intake)** |  |  |  |  |  |  |  |
| Energy: Unprocessed or minimally processed foods -G1 [LB] | **40.3** | 10.7 | 0.8 | 33.0 | 40.0 | 47.2 | 99.8 |
| Energy: Unprocessed or minimally processed foods -G1 [MB] | **37.9** | 10.6 | 0.8 | 30.6 | 37.5 | 44.7 | 99.8 |
| Energy: Unprocessed or minimally processed foods -G1 [UB] | **34.2** | 10.0 | 0.7 | 27.3 | 33.7 | 40.5 | 99.8 |
| Energy: Processed culinary ingredients -G2 [LB] | **8.5** | 6.2 | 0 | 3.4 | 7.0 | 12.5 | 57.5 |
| Energy: Processed culinary ingredients -G2 [MB] | **7.7** | 6.3 | 0 | 2.4 | 6.2 | 11.9 | 57.5 |
| Energy: Processed culinary ingredients -G2 [UB] | **7.3** | 6.3 | 0 | 1.9 | 5.7 | 11.5 | 57.5 |
| Energy: Processed foods -G3 [LB] | **25.3** | 9.8 | 0 | 18.4 | 24.9 | 31.7 | 85.1 |
| Energy: Processed foods -G3 [MB] | **21.4** | 11.3 | 0 | 11.9 | 20.2 | 29.5 | 85.1 |
| Energy: Processed foods -G3 [UB] | **11.2** | 6.1 | 0 | 7.0 | 10.1 | 14.1 | 81.0 |
| Energy: Ultra-processed foods -G4 [LB] | **25.9** | 11.5 | 0 | 17.4 | 25.5 | 33.5 | 86.3 |
| Energy: Ultra-processed foods -G4 [MB] | **32.9** | 15.5 | 0 | 20.4 | 33.2 | 44.5 | 99.1 |
| Energy: Ultra-processed foods -G4 [UB] | **47.3** | 12.9 | 0 | 38.5 | 47.9 | 56.5 | 99.1 |

LB=Lower Bound scenario, MB=Middle Bound scenario and UB=Upper Bound scenario

**Supplementary Table 5:** Distributions of the different NOVA groups (G1 to G4) by Country, using the three different scenarios (Lower, Middle and Upper bound).

| **Label** | **Country** | **N** | **Mean** | **Std** | **Min** | **P25** | **P50** | **P75** | **Max** |
| --- | --- | --- | --- | --- | --- | --- | --- | --- | --- |
| **% gram/day including alcohol** |  |  |  |  |  |  |  |  |  |
| **Unprocessed or minimally processed foods -G1 Lower** | **France** | 73,035 | **80.3** | 7.8 | 20.8 | 76.1 | 81.4 | 85.8 | 98.7 |
| **Unprocessed or minimally processed foods -G1 Middle** | **France** | 73,035 | **79.6** | 8.0 | 20.1 | 75.2 | 80.6 | 85.2 | 98.7 |
| **Unprocessed or minimally processed foods -G1 Upper** | **France** | 73,035 | **74.5** | 8.7 | 15.1 | 69.5 | 75.4 | 80.6 | 98.6 |
| **Unprocessed or minimally processed foods -G1 Lower** | **Italy** | 45,908 | **66.1** | 10.7 | 7.7 | 59.4 | 67.0 | 73.7 | 95.6 |
| **Unprocessed or minimally processed foods -G1 Middle** | **Italy** | 45,908 | **61.1** | 10.9 | 7.3 | 54.0 | 61.7 | 68.8 | 91.8 |
| **Unprocessed or minimally processed foods -G1 Upper** | **Italy** | 45,908 | **57.3** | 10.7 | 6.4 | 50.3 | 57.8 | 64.8 | 91.4 |
| **Unprocessed or minimally processed foods -G1 Lower** | **Spain** | 40,621 | **73.1** | 12.7 | 13.9 | 65.6 | 75.4 | 82.7 | 100.0 |
| **Unprocessed or minimally processed foods -G1 Middle** | **Spain** | 40,621 | **70.9** | 12.7 | 12.6 | 63.2 | 73.0 | 80.4 | 99.6 |
| **Unprocessed or minimally processed foods -G1 Upper** | **Spain** | 40,621 | **64.8** | 13.2 | 7.7 | 56.5 | 66.6 | 74.4 | 99.6 |
| **Unprocessed or minimally processed foods -G1 Lower** | **United Kingdom** | 81,097 | **75.3** | 10.5 | 9.1 | 70.3 | 77.5 | 82.7 | 100.0 |
| **Unprocessed or minimally processed foods -G1 Middle** | **United Kingdom** | 81,097 | **73.1** | 10.7 | 7.7 | 67.8 | 75.2 | 80.6 | 100.0 |
| **Unprocessed or minimally processed foods -G1 Upper** | **United Kingdom** | 81,097 | **70.1** | 10.9 | 6.5 | 64.5 | 72.1 | 77.7 | 100.0 |
| **Unprocessed or minimally processed foods -G1 Lower** | **The Netherlands** | 39,036 | **74.3** | 9.8 | 16.4 | 68.9 | 75.9 | 81.3 | 98.6 |
| **Unprocessed or minimally processed foods -G1 Middle** | **The Netherlands** | 39,036 | **73.4** | 10.1 | 16.4 | 67.8 | 75.1 | 80.7 | 98.6 |
| **Unprocessed or minimally processed foods -G1 Upper** | **The Netherlands** | 39,036 | **70.9** | 10.4 | 13.8 | 65.0 | 72.5 | 78.4 | 98.5 |
| **Unprocessed or minimally processed foods -G1 Lower** | **Germany** | 52,013 | **65.3** | 13.3 | 4.5 | 57.0 | 66.9 | 75.1 | 97.5 |
| **Unprocessed or minimally processed foods -G1 Middle** | **Germany** | 52,013 | **64.3** | 13.4 | 4.2 | 55.8 | 65.8 | 74.2 | 97.3 |
| **Unprocessed or minimally processed foods -G1 Upper** | **Germany** | 52,013 | **62.7** | 13.6 | 3.8 | 54.0 | 64.1 | 72.7 | 96.7 |
| **Unprocessed or minimally processed foods -G1 Lower** | **Sweden** | 52,750 | **75.4** | 9.7 | 16.1 | 69.9 | 76.8 | 82.4 | 98.1 |
| **Unprocessed or minimally processed foods -G1 Middle** | **Sweden** | 52,750 | **74.0** | 9.9 | 16.1 | 68.3 | 75.3 | 81.1 | 97.6 |
| **Unprocessed or minimally processed foods -G1 Upper** | **Sweden** | 52,750 | **72.5** | 10.1 | 14.2 | 66.5 | 73.8 | 79.7 | 97.6 |
| **Unprocessed or minimally processed foods -G1 Lower** | **Denmark** | 55,860 | **76.1** | 11.6 | 10.1 | 70.3 | 78.6 | 84.5 | 97.8 |
| **Unprocessed or minimally processed foods -G1 Middle** | **Denmark** | 55,860 | **74.1** | 11.6 | 9.5 | 68.2 | 76.6 | 82.5 | 97.5 |
| **Unprocessed or minimally processed foods -G1 Upper** | **Denmark** | 55,860 | **72.5** | 11.8 | 8.9 | 66.4 | 74.9 | 81.1 | 96.8 |
| **Label** | **Country** | **N** | **Mean** | **Std** | **Min** | **P25** | **P50** | **P75** | **Max** |
| **Unprocessed or minimally processed foods -G1 Lower** | **Norway** | 36,448 | **71.0** | 9.4 | 1.5 | 66.1 | 72.4 | 77.5 | 96.4 |
| **Unprocessed or minimally processed foods -G1 Middle** | **Norway** | 36,448 | **68.2** | 9.7 | 1.5 | 62.9 | 69.4 | 74.8 | 96.4 |
| **Unprocessed or minimally processed foods -G1 Upper** | **Norway** | 36,448 | **65.1** | 10.1 | 1.5 | 59.5 | 66.3 | 72.1 | 96.4 |
| **Processed culinary ingredients -G2 Lower** | **France** | 73,035 | **1.5** | 0.7 | 0 | 1.1 | 1.4 | 1.9 | 12.4 |
| **Processed culinary ingredients -G2 Middle** | **France** | 73,035 | **1.4** | 0.6 | 0 | 1.0 | 1.3 | 1.8 | 12.3 |
| **Processed culinary ingredients -G2 Upper** | **France** | 73,035 | **1.3** | 0.6 | 0 | 0.9 | 1.2 | 1.7 | 12.2 |
| **Processed culinary ingredients -G2 Lower** | **Italy** | 45,908 | **2.9** | 1.0 | 0 | 2.2 | 2.8 | 3.5 | 11.9 |
| **Processed culinary ingredients -G2 Middle** | **Italy** | 45,908 | **2.7** | 1.0 | 0 | 2.1 | 2.6 | 3.3 | 11.6 |
| **Processed culinary ingredients -G2 Upper** | **Italy** | 45,908 | **2.6** | 0.9 | 0 | 2.0 | 2.5 | 3.1 | 11.6 |
| **Processed culinary ingredients -G2 Lower** | **Spain** | 40,621 | **2.3** | 1.0 | 0 | 1.6 | 2.1 | 2.8 | 15.8 |
| **Processed culinary ingredients -G2 Middle** | **Spain** | 40,621 | **2.2** | 1.0 | 0 | 1.5 | 2.1 | 2.8 | 15.8 |
| **Processed culinary ingredients -G2 Upper** | **Spain** | 40,621 | **2.2** | 1.0 | 0 | 1.5 | 2.1 | 2.8 | 15.8 |
| **Processed culinary ingredients -G2 Lower** | **United Kingdom** | 81,097 | **0.9** | 0.7 | 0 | 0.4 | 0.7 | 1.2 | 8.1 |
| **Processed culinary ingredients -G2 Middle** | **United Kingdom** | 81,097 | **0.6** | 0.6 | 0 | 0.2 | 0.3 | 0.8 | 7.8 |
| **Processed culinary ingredients -G2 Upper** | **United Kingdom** | 81,097 | **0.5** | 0.6 | 0 | 0.1 | 0.3 | 0.7 | 7.8 |
| **Processed culinary ingredients -G2 Lower** | **The Netherlands** | 39,036 | **0.9** | 0.8 | 0.001 | 0.3 | 0.6 | 1.2 | 8.2 |
| **Processed culinary ingredients -G2 Middle** | **The Netherlands** | 39,036 | **0.8** | 0.8 | 0.001 | 0.3 | 0.5 | 1.1 | 8.2 |
| **Processed culinary ingredients -G2 Upper** | **The Netherlands** | 39,036 | **0.8** | 0.8 | 0.001 | 0.3 | 0.5 | 1.1 | 8.2 |
| **Processed culinary ingredients -G2 Lower** | **Germany** | 52,013 | **1.1** | 0.8 | 0.004 | 0.6 | 0.9 | 1.5 | 12.5 |
| **Processed culinary ingredients -G2 Middle** | **Germany** | 52,013 | **1.0** | 0.8 | 0.004 | 0.4 | 0.8 | 1.4 | 12.4 |
| **Processed culinary ingredients -G2 Upper** | **Germany** | 52,013 | **0.9** | 0.8 | 0 | 0.4 | 0.7 | 1.3 | 12.3 |
| **Processed culinary ingredients -G2 Lower** | **Sweden** | 52,750 | **1.2** | 1.0 | 0 | 0.5 | 0.9 | 1.5 | 15.9 |
| **Processed culinary ingredients -G2 Middle** | **Sweden** | 52,750 | **1.1** | 1.0 | 0 | 0.4 | 0.8 | 1.4 | 14.4 |
| **Processed culinary ingredients -G2 Upper** | **Sweden** | 52,750 | **1.0** | 1.0 | 0 | 0.3 | 0.7 | 1.3 | 14.2 |
| **Processed culinary ingredients -G2 Lower** | **Denmark** | 55,860 | **0.6** | 0.4 | 0 | 0.3 | 0.5 | 0.8 | 4.7 |
| **Processed culinary ingredients -G2 Middle** | **Denmark** | 55,860 | **0.5** | 0.4 | 0 | 0.2 | 0.3 | 0.6 | 4.5 |
| **Processed culinary ingredients -G2 Upper** | **Denmark** | 55,860 | **0.4** | 0.4 | 0 | 0.1 | 0.2 | 0.5 | 4.3 |
|  |  |  |  |  |  |  |  |  |  |
| **Label** | **Country** | **N** | **Mean** | **Std** | **Min** | **P25** | **P50** | **P75** | **Max** |
| **Processed culinary ingredients -G2 Lower** | **Norway** | 36,448 | **0.8** | 0.6 | 0 | 0.5 | 0.7 | 1.0 | 9.4 |
| **Processed culinary ingredients -G2 Middle** | **Norway** | 36,448 | **0.7** | 0.5 | 0 | 0.4 | 0.6 | 0.9 | 9.4 |
| **Processed culinary ingredients -G2 Upper** | **Norway** | 36,448 | **0.5** | 0.5 | 0 | 0.2 | 0.4 | 0.7 | 9.3 |
| **Processed foods -G3 Lower** | **France** | 73,035 | **11.7** | 6.4 | 0.03 | 7.3 | 10.5 | 14.7 | 77.3 |
| **Processed foods -G3 Middle** | **France** | 73,035 | **11.8** | 6.4 | 0.04 | 7.4 | 10.7 | 14.9 | 77.3 |
| **Processed foods -G3 Upper** | **France** | 73,035 | **9.2** | 5.7 | 0 | 5.4 | 7.8 | 11.5 | 75.6 |
| **Processed foods -G3 Lower** | **Italy** | 45,908 | **20.6** | 10.1 | 0.4 | 13.1 | 18.9 | 26.3 | 78.7 |
| **Processed foods -G3 Middle** | **Italy** | 45,908 | **25.8** | 10.4 | 1.2 | 18.1 | 24.4 | 32.1 | 79.6 |
| **Processed foods -G3 Upper** | **Italy** | 45,908 | **14.2** | 9.2 | 0 | 7.2 | 11.8 | 19.1 | 74.3 |
| **Processed foods -G3 Lower** | **Spain** | 40,621 | **16.7** | 11.6 | 0 | 8.2 | 13.5 | 22.5 | 81.9 |
| **Processed foods -G3 Middle** | **Spain** | 40,621 | **18.8** | 11.7 | 0 | 10.3 | 15.9 | 24.9 | 82.9 |
| **Processed foods -G3 Upper** | **Spain** | 40,621 | **14.0** | 10.6 | 0 | 6.4 | 10.4 | 18.9 | 80.9 |
| **Processed foods -G3 Lower** | **United Kingdom** | 81,097 | **9.6** | 7.0 | 0 | 5.2 | 7.8 | 11.7 | 76.9 |
| **Processed foods -G3 Middle** | **United Kingdom** | 81,097 | **7.8** | 6.6 | 0 | 3.8 | 6.0 | 9.4 | 74.3 |
| **Processed foods -G3 Upper** | **United Kingdom** | 81,097 | **9.0** | 6.7 | 0 | 4.8 | 7.1 | 10.8 | 75.2 |
| **Processed foods -G3 Lower** | **The Netherlands** | 39,036 | **11.3** | 7.1 | 0.1 | 6.7 | 9.3 | 13.6 | 78.6 |
| **Processed foods -G3 Middle** | **The Netherlands** | 39,036 | **10.8** | 7.0 | 0.2 | 6.3 | 8.9 | 12.9 | 78.4 |
| **Processed foods -G3 Upper** | **The Netherlands** | 39,036 | **6.9** | 6.5 | 0.06 | 3.1 | 4.7 | 8.0 | 76.9 |
| **Processed foods -G3 Lower** | **Germany** | 52,013 | **17.4** | 10.8 | 0.1 | 9.9 | 14.5 | 21.9 | 92.2 |
| **Processed foods -G3 Middle** | **Germany** | 52,013 | **18.2** | 10.8 | 0.2 | 10.7 | 15.3 | 22.7 | 92.4 |
| **Processed foods -G3 Upper** | **Germany** | 52,013 | **12.9** | 10.5 | 0.2 | 5.7 | 9.4 | 16.6 | 90.3 |
| **Processed foods -G3 Lower** | **Sweden** | 52,750 | **11.9** | 6.7 | 0.2 | 7.3 | 10.4 | 14.9 | 75.2 |
| **Processed foods -G3 Middle** | **Sweden** | 52,750 | **11.8** | 6.7 | 0.02 | 7.2 | 10.2 | 14.7 | 76.2 |
| **Processed foods -G3 Upper** | **Sweden** | 52,750 | **7.4** | 6.2 | 0 | 3.0 | 5.6 | 9.9 | 75.7 |
| **Processed foods -G3 Lower** | **Denmark** | 55,860 | **15.2** | 10.0 | 0.5 | 8.4 | 12.4 | 18.5 | 84.8 |
| **Processed foods -G3 Middle** | **Denmark** | 55,860 | **12.1** | 9.8 | 0.2 | 5.6 | 9.1 | 14.9 | 83.5 |
| **Processed foods -G3 Upper** | **Denmark** | 55,860 | **11.4** | 9.9 | 0.04 | 4.9 | 8.3 | 14.3 | 83.5 |
|  |  |  |  |  |  |  |  |  |  |
| **Label** | **Country** | **N** | **Mean** | **Std** | **Min** | **P25** | **P50** | **P75** | **Max** |
| **Processed foods -G3 Lower** | **Norway** | 36,448 | **14.3** | 5.8 | 0.2 | 10.3 | 13.6 | 17.4 | 53.7 |
| **Processed foods -G3 Middle** | **Norway** | 36,448 | **8.3** | 4.2 | 0 | 5.6 | 7.6 | 10.1 | 52.4 |
| **Processed foods -G3 Upper** | **Norway** | 36,448 | **6.6** | 4.2 | 0 | 3.8 | 5.7 | 8.2 | 50.7 |
| **Ultra-processed foods -G4 Lower** | **France** | 73,035 | **6.5** | 4.2 | 0.01 | 3.6 | 5.6 | 8.2 | 60.6 |
| **Ultra-processed foods -G4 Middle** | **France** | 73,035 | **7.1** | 4.5 | 0.01 | 4.1 | 6.2 | 9.1 | 61.5 |
| **Ultra-processed foods -G4 Upper** | **France** | 73,035 | **14.9** | 6.5 | 0 | 10.3 | 14.2 | 18.6 | 67.0 |
| **Ultra-processed foods -G4 Lower** | **Italy** | 45,908 | **10.4** | 6.6 | 0 | 6.0 | 8.9 | 13.2 | 75.0 |
| **Ultra-processed foods -G4 Middle** | **Italy** | 45,908 | **10.4** | 6.6 | 0 | 5.9 | 8.9 | 13.2 | 75.0 |
| **Ultra-processed foods -G4 Upper** | **Italy** | 45,908 | **25.9** | 8.6 | 2.1 | 20.0 | 25.1 | 30.8 | 87.5 |
| **Ultra-processed foods -G4 Lower** | **Spain** | 40,621 | **7.9** | 6.4 | 0 | 3.6 | 6.2 | 10.4 | 68.4 |
| **Ultra-processed foods -G4 Middle** | **Spain** | 40,621 | **8.0** | 6.4 | 0 | 3.6 | 6.3 | 10.5 | 68.4 |
| **Ultra-processed foods -G4 Upper** | **Spain** | 40,621 | **19.0** | 8.2 | 0 | 13.3 | 18.0 | 23.5 | 80.5 |
| **Ultra-processed foods -G4 Lower** | **United Kingdom** | 81,097 | **14.2** | 8.5 | 0 | 8.4 | 12.0 | 17.4 | 89.6 |
| **Ultra-processed foods -G4 Middle** | **United Kingdom** | 81,097 | **18.5** | 9.1 | 0 | 12.4 | 16.5 | 22.2 | 90.5 |
| **Ultra-processed foods -G4 Upper** | **United Kingdom** | 81,097 | **20.4** | 9.2 | 0 | 14.2 | 18.6 | 24.5 | 91.1 |
| **Ultra-processed foods -G4 Lower** | **The Netherlands** | 39,036 | **13.5** | 6.5 | 0.8 | 8.9 | 12.2 | 16.6 | 68.0 |
| **Ultra-processed foods -G4 Middle** | **The Netherlands** | 39,036 | **15.0** | 7.0 | 0.8 | 10.0 | 13.7 | 18.3 | 69.8 |
| **Ultra-processed foods -G4 Upper** | **The Netherlands** | 39,036 | **21.4** | 8.1 | 1.2 | 15.7 | 20.3 | 25.8 | 75.6 |
| **Ultra-processed foods -G4 Lower** | **Germany** | 52,013 | **16.1** | 8.9 | 0 | 9.7 | 14.2 | 20.4 | 85.1 |
| **Ultra-processed foods -G4 Middle** | **Germany** | 52,013 | **16.5** | 9.0 | 0 | 10.1 | 14.6 | 20.8 | 85.7 |
| **Ultra-processed foods -G4 Upper** | **Germany** | 52,013 | **23.5** | 9.9 | 0 | 16.5 | 22.1 | 28.8 | 88.5 |
| **Ultra-processed foods -G4 Lower** | **Sweden** | 52,750 | **11.5** | 6.5 | 0 | 7.1 | 10.0 | 14.3 | 80.8 |
| **Ultra-processed foods -G4 Middle** | **Sweden** | 52,750 | **13.2** | 6.8 | 0 | 8.6 | 11.8 | 16.2 | 80.8 |
| **Ultra-processed foods -G4 Upper** | **Sweden** | 52,750 | **19.2** | 8.0 | 1.1 | 13.6 | 18.0 | 23.4 | 83.6 |
| **Ultra-processed foods -G4 Lower** | **Denmark** | 55,860 | **8.2** | 7.0 | 0 | 3.7 | 6.0 | 10.0 | 81.2 |
| **Ultra-processed foods -G4 Middle** | **Denmark** | 55,860 | **13.3** | 7.4 | 0 | 8.4 | 11.5 | 16.0 | 82.9 |
| **Ultra-processed foods -G4 Upper** | **Denmark** | 55,860 | **15.7** | 7.8 | 1.0 | 10.4 | 13.9 | 18.9 | 87.2 |
|  |  |  |  |  |  |  |  |  |  |
| **Label** | **Country** | **N** | **Mean** | **Std** | **Min** | **P25** | **P50** | **P75** | **Max** |
| **Ultra-processed foods -G4 Lower** | **Norway** | 36,448 | **13.8** | 7.8 | 0.2 | 8.8 | 12.1 | 16.6 | 79.2 |
| **Ultra-processed foods -G4 Middle** | **Norway** | 36,448 | **22.8** | 8.8 | 1.0 | 16.9 | 21.3 | 26.9 | 85.3 |
| **Ultra-processed foods -G4 Upper** | **Norway** | 36,448 | **27.7** | 9.4 | 1.8 | 21.3 | 26.4 | 32.7 | 86.1 |
| **%kcal/day including alcohol** |  |  |  |  |  |  |  |  |  |
| **Energy: Unprocessed or minimally processed foods -G1 Lower** | **France** | 73,035 | **40.8** | 9.7 | 4.2 | 34.0 | 40.2 | 46.8 | 89.0 |
| **Energy: Unprocessed or minimally processed foods -G1 Middle** | **France** | 73,035 | **39.8** | 9.8 | 4.2 | 33.0 | 39.2 | 45.9 | 88.2 |
| **Energy: Unprocessed or minimally processed foods -G1 Upper** | **France** | 73,035 | **35.1** | 9.6 | 1.3 | 28.4 | 34.3 | 41.0 | 85.6 |
| **Energy: Unprocessed or minimally processed foods -G1 Lower** | **Italy** | 45,908 | **37.2** | 8.1 | 5.1 | 31.7 | 36.9 | 42.3 | 80.6 |
| **Energy: Unprocessed or minimally processed foods -G1 Middle** | **Italy** | 45,908 | **34.7** | 7.7 | 4.9 | 29.4 | 34.4 | 39.7 | 76.3 |
| **Energy: Unprocessed or minimally processed foods -G1 Upper** | **Italy** | 45,908 | **31.6** | 7.5 | 4.6 | 26.5 | 31.3 | 36.4 | 75.2 |
| **Energy: Unprocessed or minimally processed foods -G1 Lower** | **Spain** | 40,621 | **44.5** | 10.1 | 5.8 | 37.5 | 43.9 | 50.9 | 100.0 |
| **Energy: Unprocessed or minimally processed foods -G1 Middle** | **Spain** | 40,621 | **42.7** | 10.1 | 5.7 | 35.8 | 42.0 | 49.0 | 99.6 |
| **Energy: Unprocessed or minimally processed foods -G1 Upper** | **Spain** | 40,621 | **36.4** | 9.6 | 0.4 | 29.7 | 35.6 | 42.2 | 99.6 |
| **Energy: Unprocessed or minimally processed foods -G1 Lower** | **United Kingdom** | 81,097 | **41.8** | 10.7 | 5.5 | 34.4 | 41.3 | 48.6 | 100.0 |
| **Energy: Unprocessed or minimally processed foods -G1 Middle** | **United Kingdom** | 81,097 | **37.8** | 10.9 | 5.1 | 30.1 | 37.0 | 44.6 | 100.0 |
| **Energy: Unprocessed or minimally processed foods -G1 Upper** | **United Kingdom** | 81,097 | **34.3** | 10.2 | 3.1 | 27.1 | 33.6 | 40.7 | 100.0 |
| **Energy: Unprocessed or minimally processed foods -G1 Lower** | **The Netherlands** | 39,036 | **35.1** | 8.4 | 4.9 | 29.2 | 34.6 | 40.5 | 85.8 |
| **Energy: Unprocessed or minimally processed foods -G1 Middle** | **The Netherlands** | 39,036 | **34.1** | 8.6 | 3.4 | 28.1 | 33.7 | 39.7 | 85.8 |
| **Energy: Unprocessed or minimally processed foods -G1 Upper** | **The Netherlands** | 39,036 | **31.9** | 8.4 | 3.1 | 26.1 | 31.5 | 37.3 | 82.3 |
| **Energy: Unprocessed or minimally processed foods -G1 Lower** | **Germany** | 52,013 | **26.4** | 7.8 | 2.2 | 21.0 | 25.5 | 30.7 | 82.9 |
| **Energy: Unprocessed or minimally processed foods -G1 Middle** | **Germany** | 52,013 | **24.7** | 7.8 | 2.2 | 19.4 | 23.7 | 28.9 | 82.1 |
| **Energy: Unprocessed or minimally processed foods -G1 Upper** | **Germany** | 52,013 | **22.3** | 7.5 | 2.2 | 17.1 | 21.3 | 26.4 | 81.8 |
| **Energy: Unprocessed or minimally processed foods -G1 Lower** | **Sweden** | 52,750 | **39.4** | 10.6 | 1.2 | 31.8 | 38.9 | 46.5 | 92.3 |
| **Energy: Unprocessed or minimally processed foods -G1 Middle** | **Sweden** | 52,750 | **37.0** | 10.4 | 1.2 | 29.5 | 36.4 | 43.9 | 90.1 |
| **Energy: Unprocessed or minimally processed foods -G1 Upper** | **Sweden** | 52,750 | **34.6** | 10.2 | 1.2 | 27.4 | 33.9 | 41.1 | 89.2 |
| **Energy: Unprocessed or minimally processed foods -G1 Lower** | **Denmark** | 55,860 | **37.3** | 9.1 | 5.2 | 30.9 | 36.8 | 43.1 | 92.8 |
| **Energy: Unprocessed or minimally processed foods -G1 Middle** | **Denmark** | 55,860 | **34.8** | 8.8 | 4.2 | 28.6 | 34.3 | 40.3 | 84.2 |
| **Energy: Unprocessed or minimally processed foods -G1 Upper** | **Denmark** | 55,860 | **31.6** | 8.7 | 3.6 | 25.6 | 31.1 | 37.1 | 81.5 |
|  |  |  |  |  |  |  |  |  |  |
| **Label** | **Country** | **N** | **Mean** | **Std** | **Min** | **P25** | **P50** | **P75** | **Max** |
| **Energy: Unprocessed or minimally processed foods -G1 Lower** | **Norway** | 36,448 | **40.7** | 8.9 | 0.8 | 34.7 | 40.4 | 46.3 | 89.1 |
| **Energy: Unprocessed or minimally processed foods -G1 Middle** | **Norway** | 36,448 | **37.6** | 8.8 | 0.8 | 31.7 | 37.2 | 43.1 | 89.1 |
| **Energy: Unprocessed or minimally processed foods -G1 Upper** | **Norway** | 36,448 | **33.7** | 8.8 | 0.8 | 27.7 | 33.1 | 39.1 | 89.1 |
| **Energy: Processed culinary ingredients -G2 Lower** | **France** | 73,035 | **10.9** | 4.3 | 0 | 7.9 | 10.4 | 13.3 | 48.8 |
| **Energy: Processed culinary ingredients -G2 Middle** | **France** | 73,035 | **10.5** | 4.3 | 0 | 7.5 | 10.0 | 12.9 | 48.8 |
| **Energy: Processed culinary ingredients -G2 Upper** | **France** | 73,035 | **10.0** | 4.3 | 0 | 7.0 | 9.5 | 12.5 | 45.5 |
| **Energy: Processed culinary ingredients -G2 Lower** | **Italy** | 45,908 | **15.9** | 4.6 | 0 | 12.7 | 15.5 | 18.5 | 47.9 |
| **Energy: Processed culinary ingredients -G2 Middle** | **Italy** | 45,908 | **15.5** | 4.6 | 0 | 12.3 | 15.1 | 18.2 | 47.9 |
| **Energy: Processed culinary ingredients -G2 Upper** | **Italy** | 45,908 | **15.0** | 4.6 | 0 | 11.9 | 14.6 | 17.7 | 47.9 |
| **Energy: Processed culinary ingredients -G2 Lower** | **Spain** | 40,621 | **13.9** | 5.0 | 0 | 10.4 | 13.4 | 17.0 | 57.4 |
| **Energy: Processed culinary ingredients -G2 Middle** | **Spain** | 40,621 | **13.9** | 5.0 | 0 | 10.3 | 13.4 | 16.9 | 57.4 |
| **Energy: Processed culinary ingredients -G2 Upper** | **Spain** | 40,621 | **13.9** | 5.1 | 0 | 10.3 | 13.3 | 16.9 | 57.4 |
| **Energy: Processed culinary ingredients -G2 Lower** | **United Kingdom** | 81,097 | **5.2** | 4.2 | 0 | 2.3 | 3.9 | 6.8 | 40.4 |
| **Energy: Processed culinary ingredients -G2 Middle** | **United Kingdom** | 81,097 | **3.2** | 3.8 | 0 | 0.6 | 1.5 | 4.4 | 38.8 |
| **Energy: Processed culinary ingredients -G2 Upper** | **United Kingdom** | 81,097 | **3.0** | 3.8 | 0 | 0.4 | 1.3 | 4.3 | 38.8 |
| **Energy: Processed culinary ingredients -G2 Lower** | **The Netherlands** | 39,036 | **6.6** | 4.4 | 0.01 | 3.4 | 5.5 | 8.7 | 49.0 |
| **Energy: Processed culinary ingredients -G2 Middle** | **The Netherlands** | 39,036 | **5.5** | 4.2 | 0.01 | 2.3 | 4.3 | 7.5 | 48.6 |
| **Energy: Processed culinary ingredients -G2 Upper** | **The Netherlands** | 39,036 | **5.4** | 4.2 | 0.01 | 2.3 | 4.3 | 7.5 | 48.6 |
| **Energy: Processed culinary ingredients -G2 Lower** | **Germany** | 52,013 | **8.0** | 4.9 | 0.01 | 4.3 | 6.9 | 10.6 | 47.7 |
| **Energy: Processed culinary ingredients -G2 Middle** | **Germany** | 52,013 | **7.4** | 4.9 | 0.01 | 3.7 | 6.4 | 10.1 | 47.4 |
| **Energy: Processed culinary ingredients -G2 Upper** | **Germany** | 52,013 | **7.0** | 4.9 | 0 | 3.3 | 5.9 | 9.6 | 46.8 |
| **Energy: Processed culinary ingredients -G2 Lower** | **Sweden** | 52,750 | **5.4** | 4.9 | 0 | 2.2 | 4.0 | 7.0 | 49.4 |
| **Energy: Processed culinary ingredients -G2 Middle** | **Sweden** | 52,750 | **5.2** | 4.9 | 0 | 2.0 | 3.8 | 6.8 | 49.4 |
| **Energy: Processed culinary ingredients -G2 Upper** | **Sweden** | 52,750 | **4.8** | 4.8 | 0 | 1.6 | 3.3 | 6.2 | 49.4 |
| **Energy: Processed culinary ingredients -G2 Lower** | **Denmark** | 55,860 | **3.7** | 3.2 | 0 | 1.4 | 2.8 | 5.0 | 27.6 |
| **Energy: Processed culinary ingredients -G2 Middle** | **Denmark** | 55,860 | **3.1** | 3.1 | 0 | 0.8 | 2.2 | 4.3 | 27.3 |
| **Energy: Processed culinary ingredients -G2 Upper** | **Denmark** | 55,860 | **2.7** | 3.1 | 0 | 0.4 | 1.6 | 3.7 | 27.1 |
|  |  |  |  |  |  |  |  |  |  |
| **Label** | **Country** | **N** | **Mean** | **Std** | **Min** | **P25** | **P50** | **P75** | **Max** |
| **Energy: Processed culinary ingredients -G2 Lower** | **Norway** | 36,448 | **4.4** | 3.0 | 0 | 2.5 | 3.6 | 5.5 | 34.9 |
| **Energy: Processed culinary ingredients -G2 Middle** | **Norway** | 36,448 | **4.0** | 3.0 | 0 | 2.1 | 3.1 | 5.0 | 34.9 |
| **Energy: Processed culinary ingredients -G2 Upper** | **Norway** | 36,448 | **3.1** | 2.9 | 0 | 1.3 | 2.1 | 3.9 | 34.7 |
| **Energy: Processed foods -G3 Lower** | **France** | 73,035 | **29.9** | 10.8 | 0.03 | 22.4 | 29.6 | 37.0 | 85.2 |
| **Energy: Processed foods -G3 Middle** | **France** | 73,035 | **30.1** | 10.8 | 0.03 | 22.6 | 29.8 | 37.2 | 85.2 |
| **Energy: Processed foods -G3 Upper** | **France** | 73,035 | **19.3** | 8.7 | 0 | 13.3 | 18.0 | 24.0 | 79.7 |
| **Energy: Processed foods -G3 Lower** | **Italy** | 45,908 | **31.4** | 9.9 | 0.7 | 24.6 | 31.1 | 37.9 | 82.7 |
| **Energy: Processed foods -G3 Middle** | **Italy** | 45,908 | **34.4** | 9.7 | 2.1 | 27.7 | 34.1 | 40.8 | 82.9 |
| **Energy: Processed foods -G3 Upper** | **Italy** | 45,908 | **16.6** | 7.8 | 0 | 11.0 | 15.5 | 21.0 | 65.2 |
| **Energy: Processed foods -G3 Lower** | **Spain** | 40,621 | **26.0** | 11.3 | 0 | 18.0 | 25.6 | 33.6 | 74.6 |
| **Energy: Processed foods -G3 Middle** | **Spain** | 40,621 | **27.7** | 11.3 | 0 | 19.7 | 27.4 | 35.4 | 74.9 |
| **Energy: Processed foods -G3 Upper** | **Spain** | 40,621 | **15.0** | 8.4 | 0 | 8.6 | 13.4 | 19.8 | 69.4 |
| **Energy: Processed foods -G3 Lower** | **United Kingdom** | 81,097 | **20.6** | 8.6 | 0 | 14.4 | 19.6 | 25.7 | 70.4 |
| **Energy: Processed foods -G3 Middle** | **United Kingdom** | 81,097 | **14.3** | 6.9 | 0 | 9.5 | 13.3 | 17.9 | 68.7 |
| **Energy: Processed foods -G3 Upper** | **United Kingdom** | 81,097 | **13.5** | 6.8 | 0 | 8.8 | 12.1 | 16.6 | 73.1 |
| **Energy: Processed foods -G3 Lower** | **The Netherlands** | 39,036 | **27.8** | 8.1 | 1.0 | 22.3 | 27.3 | 32.8 | 75.6 |
| **Energy: Processed foods -G3 Middle** | **The Netherlands** | 39,036 | **27.1** | 8.1 | 0.8 | 21.7 | 26.6 | 32.0 | 75.3 |
| **Energy: Processed foods -G3 Upper** | **The Netherlands** | 39,036 | **12.5** | 6.0 | 0.08 | 8.4 | 11.4 | 15.4 | 74.6 |
| **Energy: Processed foods -G3 Lower** | **Germany** | 52,013 | **33.5** | 10.2 | 0.4 | 26.4 | 33.1 | 40.1 | 90.0 |
| **Energy: Processed foods -G3 Middle** | **Germany** | 52,013 | **35.0** | 10.0 | 0.6 | 28.1 | 34.6 | 41.4 | 90.0 |
| **Energy: Processed foods -G3 Upper** | **Germany** | 52,013 | **16.1** | 7.8 | 0.2 | 10.7 | 14.7 | 19.9 | 90.0 |
| **Energy: Processed foods -G3 Lower** | **Sweden** | 52,750 | **24.9** | 8.1 | 0.9 | 19.2 | 24.3 | 29.9 | 72.5 |
| **Energy: Processed foods -G3 Middle** | **Sweden** | 52,750 | **24.2** | 8.0 | 0.2 | 18.6 | 23.6 | 29.1 | 72.5 |
| **Energy: Processed foods -G3 Upper** | **Sweden** | 52,750 | **9.9** | 5.7 | 0 | 5.7 | 8.9 | 13.1 | 59.5 |
| **Energy: Processed foods -G3 Lower** | **Denmark** | 55,860 | **33.7** | 9.8 | 1.4 | 26.8 | 33.2 | 40.0 | 77.8 |
| **Energy: Processed foods -G3 Middle** | **Denmark** | 55,860 | **18.6** | 8.2 | 0.1 | 12.9 | 17.2 | 22.9 | 73.2 |
| **Energy: Processed foods -G3 Upper** | **Denmark** | 55,860 | **16.8** | 8.4 | 0.07 | 10.9 | 15.3 | 21.2 | 72.4 |
|  |  |  |  |  |  |  |  |  |  |
| **Label** | **Country** | **N** | **Mean** | **Std** | **Min** | **P25** | **P50** | **P75** | **Max** |
| **Energy: Processed foods -G3 Lower** | **Norway** | 36,448 | **29.6** | 9.1 | 0.2 | 23.8 | 29.7 | 35.6 | 77.5 |
| **Energy: Processed foods -G3 Middle** | **Norway** | 36,448 | **12.4** | 4.8 | 0 | 8.9 | 11.9 | 15.2 | 43.3 |
| **Energy: Processed foods -G3 Upper** | **Norway** | 36,448 | **11.0** | 4.9 | 0 | 7.5 | 10.4 | 13.9 | 45.1 |
| **Energy: Ultra-processed foods -G4 Lower** | **France** | 73,035 | **18.5** | 8.6 | 0.1 | 12.3 | 17.3 | 23.4 | 76.8 |
| **Energy: Ultra-processed foods -G4 Middle** | **France** | 73,035 | **19.6** | 8.9 | 0.1 | 13.2 | 18.5 | 24.8 | 76.8 |
| **Energy: Ultra-processed foods -G4 Upper** | **France** | 73,035 | **35.6** | 10.5 | 0 | 28.6 | 35.6 | 42.5 | 85.1 |
| **Energy: Ultra-processed foods -G4 Lower** | **Italy** | 45,908 | **15.5** | 7.6 | 0 | 10.1 | 14.4 | 19.6 | 68.4 |
| **Energy: Ultra-processed foods -G4 Middle** | **Italy** | 45,908 | **15.5** | 7.6 | 0 | 10.1 | 14.4 | 19.6 | 68.4 |
| **Energy: Ultra-processed foods -G4 Upper** | **Italy** | 45,908 | **36.7** | 9.8 | 3.1 | 30.1 | 36.4 | 43.1 | 84.6 |
| **Energy: Ultra-processed foods -G4 Lower** | **Spain** | 40,621 | **15.5** | 9.3 | 0 | 8.6 | 14.0 | 20.7 | 80.8 |
| **Energy: Ultra-processed foods -G4 Middle** | **Spain** | 40,621 | **15.7** | 9.3 | 0 | 8.8 | 14.2 | 20.9 | 80.8 |
| **Energy: Ultra-processed foods -G4 Upper** | **Spain** | 40,621 | **34.8** | 10.4 | 0 | 27.8 | 34.6 | 41.6 | 82.5 |
| **Energy: Ultra-processed foods -G4 Lower** | **United Kingdom** | 81,097 | **32.4** | 10.5 | 0 | 25.1 | 31.9 | 39.2 | 83.9 |
| **Energy: Ultra-processed foods -G4 Middle** | **United Kingdom** | 81,097 | **44.7** | 11.3 | 0 | 37.0 | 44.7 | 52.4 | 89.8 |
| **Energy: Ultra-processed foods -G4 Upper** | **United Kingdom** | 81,097 | **49.2** | 11.4 | 0 | 41.6 | 49.4 | 57.1 | 89.8 |
| **Energy: Ultra-processed foods -G4 Lower** | **The Netherlands** | 39,036 | **30.5** | 7.8 | 4.1 | 25.3 | 30.3 | 35.4 | 69.7 |
| **Energy: Ultra-processed foods -G4 Middle** | **The Netherlands** | 39,036 | **33.3** | 8.2 | 4.7 | 27.8 | 33.0 | 38.5 | 70.8 |
| **Energy: Ultra-processed foods -G4 Upper** | **The Netherlands** | 39,036 | **50.1** | 8.9 | 5.6 | 44.4 | 50.3 | 56.0 | 89.8 |
| **Energy: Ultra-processed foods -G4 Lower** | **Germany** | 52,013 | **32.1** | 10.2 | 0 | 25.0 | 31.7 | 38.8 | 79.6 |
| **Energy: Ultra-processed foods -G4 Middle** | **Germany** | 52,013 | **32.9** | 10.3 | 0 | 25.7 | 32.5 | 39.6 | 80.0 |
| **Energy: Ultra-processed foods -G4 Upper** | **Germany** | 52,013 | **54.6** | 10.4 | 0 | 48.0 | 55.2 | 61.8 | 89.4 |
| **Energy: Ultra-processed foods -G4 Lower** | **Sweden** | 52,750 | **30.3** | 9.1 | 0 | 23.9 | 29.8 | 36.2 | 76.3 |
| **Energy: Ultra-processed foods -G4 Middle** | **Sweden** | 52,750 | **33.5** | 9.4 | 0 | 27.0 | 33.2 | 39.8 | 77.0 |
| **Energy: Ultra-processed foods -G4 Upper** | **Sweden** | 52,750 | **50.7** | 9.8 | 7.7 | 44.2 | 50.8 | 57.3 | 93.1 |
| **Energy: Ultra-processed foods -G4 Lower** | **Denmark** | 55,860 | **25.3** | 9.3 | 0 | 18.8 | 24.6 | 30.9 | 81.6 |
| **Energy: Ultra-processed foods -G4 Middle** | **Denmark** | 55,860 | **43.5** | 10.0 | 1.9 | 36.8 | 43.3 | 50.0 | 88.5 |
| **Energy: Ultra-processed foods -G4 Upper** | **Denmark** | 55,860 | **48.9** | 10.3 | 3.3 | 42.0 | 48.9 | 55.7 | 90.3 |
|  |  |  |  |  |  |  |  |  |  |
| **Label** | **Country** | **N** | **Mean** | **Std** | **Min** | **P25** | **P50** | **P75** | **Max** |
| **Energy: Ultra-processed foods -G4 Lower** | **Norway** | 36,448 | **25.2** | 7.3 | 0.5 | 20.3 | 24.8 | 29.6 | 68.3 |
| **Energy: Ultra-processed foods -G4 Middle** | **Norway** | 36,448 | **46.0** | 9.0 | 3.2 | 40.2 | 46.1 | 52.0 | 97.5 |
| **Energy: Ultra-processed foods -G4 Upper** | **Norway** | 36,448 | **52.2** | 9.2 | 6.0 | 46.3 | 52.5 | 58.4 | 97.5 |
| **grams/day** |  |  |  |  |  |  |  |  |  |
| **Unprocessed or minimally processed foods -G1 Lower** | **France** | 73,035 | **2,516.0** | 798.6 | 199.2 | 1,968.1 | 2,412.5 | 2,939.6 | 16,347.4 |
| **Unprocessed or minimally processed foods -G1 Middle** | **France** | 73,035 | **2,493.9** | 798.3 | 187.9 | 1,945.9 | 2,389.9 | 2,917.1 | 16,347.4 |
| **Unprocessed or minimally processed foods -G1 Upper** | **France** | 73,035 | **2,341.6** | 782.0 | 178.0 | 1,801.8 | 2,235.2 | 2,755.1 | 16,033.0 |
| **Unprocessed or minimally processed foods -G1 Lower** | **Italy** | 45,908 | **1,219.5** | 389.8 | 116.2 | 952.5 | 1,173.3 | 1,429.2 | 4,388.9 |
| **Unprocessed or minimally processed foods -G1 Middle** | **Italy** | 45,908 | **1,126.1** | 366.0 | 109.4 | 874.4 | 1,082.2 | 1,325.1 | 4,221.3 |
| **Unprocessed or minimally processed foods -G1 Upper** | **Italy** | 45,908 | **1,057.6** | 353.5 | 107.2 | 816.4 | 1,014.5 | 1,247.7 | 4,184.0 |
| **Unprocessed or minimally processed foods -G1 Lower** | **Spain** | 40,621 | **1,419.5** | 398.5 | 253.1 | 1,142.3 | 1,379.4 | 1,652.8 | 4,786.9 |
| **Unprocessed or minimally processed foods -G1 Middle** | **Spain** | 40,621 | **1,376.3** | 391.8 | 206.5 | 1,104.3 | 1,336.3 | 1,605.9 | 4,721.4 |
| **Unprocessed or minimally processed foods -G1 Upper** | **Spain** | 40,621 | **1,256.7** | 379.9 | 121.4 | 995.1 | 1,215.9 | 1,476.5 | 4,721.4 |
| **Unprocessed or minimally processed foods -G1 Lower** | **United Kingdom** | 81,097 | **2,133.2** | 619.4 | 239.4 | 1,726.3 | 2,125.2 | 2,518.1 | 7,531.3 |
| **Unprocessed or minimally processed foods -G1 Middle** | **United Kingdom** | 81,097 | **2,073.1** | 612.7 | 186.2 | 1,671.8 | 2,064.7 | 2,452.3 | 7,493.4 |
| **Unprocessed or minimally processed foods -G1 Upper** | **United Kingdom** | 81,097 | **1,989.4** | 602.6 | 150.4 | 1,596.5 | 1,985.3 | 2,365.4 | 6,789.1 |
| **Unprocessed or minimally processed foods -G1 Lower** | **The Netherlands** | 39,036 | **2,214.9** | 622.4 | 361.8 | 1,792.3 | 2,157.7 | 2,566.6 | 9,858.6 |
| **Unprocessed or minimally processed foods -G1 Middle** | **The Netherlands** | 39,036 | **2,189.9** | 624.6 | 332.0 | 1,767.2 | 2,133.1 | 2,543.7 | 9,848.6 |
| **Unprocessed or minimally processed foods -G1 Upper** | **The Netherlands** | 39,036 | **2,116.6** | 618.6 | 310.2 | 1,696.0 | 2,058.9 | 2,466.6 | 9,819.9 |
| **Unprocessed or minimally processed foods -G1 Lower** | **Germany** | 52,013 | **1,887.5** | 771.6 | 118.7 | 1,366.2 | 1,751.7 | 2,244.4 | 14,460.0 |
| **Unprocessed or minimally processed foods -G1 Middle** | **Germany** | 52,013 | **1,860.6** | 770.9 | 118.7 | 1,339.0 | 1,723.7 | 2,216.5 | 14,425.0 |
| **Unprocessed or minimally processed foods -G1 Upper** | **Germany** | 52,013 | **1,814.4** | 766.9 | 114.5 | 1,297.7 | 1,677.4 | 2,167.3 | 14,373.6 |
| **Unprocessed or minimally processed foods -G1 Lower** | **Sweden** | 52,750 | **1,994.4** | 750.0 | 129.5 | 1,465.1 | 1,871.9 | 2,386.5 | 7,799.9 |
| **Unprocessed or minimally processed foods -G1 Middle** | **Sweden** | 52,750 | **1,959.4** | 746.1 | 100.9 | 1,433.4 | 1,836.6 | 2,348.7 | 7,750.1 |
| **Unprocessed or minimally processed foods -G1 Upper** | **Sweden** | 52,750 | **1,923.2** | 746.8 | 89.5 | 1,397.2 | 1,798.4 | 2,314.2 | 7,750.1 |
| **Unprocessed or minimally processed foods -G1 Lower** | **Denmark** | 55,860 | **2,802.1** | 808.5 | 206.3 | 2,245.6 | 2,741.8 | 3,293.1 | 7,994.0 |
| **Unprocessed or minimally processed foods -G1 Middle** | **Denmark** | 55,860 | **2,732.5** | 800.3 | 186.4 | 2,183.8 | 2,672.7 | 3,219.0 | 7,889.8 |
| **Unprocessed or minimally processed foods -G1 Upper** | **Denmark** | 55,860 | **2,675.5** | 798.3 | 180.3 | 2,127.2 | 2,614.7 | 3,158.8 | 7,820.6 |
|  |  |  |  |  |  |  |  |  |  |
| **Label** | **Country** | **N** | **Mean** | **Std** | **Min** | **P25** | **P50** | **P75** | **Max** |
| **Unprocessed or minimally processed foods -G1 Lower** | **Norway** | 36,448 | **1,240.5** | 387.6 | 5.4 | 972.1 | 1,217.8 | 1,483.4 | 4,117.7 |
| **Unprocessed or minimally processed foods -G1 Middle** | **Norway** | 36,448 | **1,191.8** | 381.6 | 5.4 | 927.9 | 1,168.5 | 1,431.4 | 4,051.1 |
| **Unprocessed or minimally processed foods -G1 Upper** | **Norway** | 36,448 | **1,140.4** | 376.0 | 5.4 | 880.7 | 1,116.9 | 1,375.6 | 3,933.7 |
| **Processed culinary ingredients -G2 Lower** | **France** | 73,035 | **45.9** | 19.8 | 0 | 32.1 | 43.5 | 56.8 | 318.9 |
| **Processed culinary ingredients -G2 Middle** | **France** | 73,035 | **42.7** | 19.4 | 0 | 29.1 | 40.3 | 53.3 | 317.6 |
| **Processed culinary ingredients -G2 Upper** | **France** | 73,035 | **39.6** | 19.3 | 0 | 26.0 | 37.3 | 50.2 | 309.4 |
| **Processed culinary ingredients -G2 Lower** | **Italy** | 45,908 | **53.3** | 21.8 | 0 | 38.1 | 50.3 | 65.1 | 285.1 |
| **Processed culinary ingredients -G2 Middle** | **Italy** | 45,908 | **50.1** | 21.1 | 0 | 35.4 | 47.3 | 61.5 | 277.6 |
| **Processed culinary ingredients -G2 Upper** | **Italy** | 45,908 | **47.7** | 20.4 | 0 | 33.4 | 44.8 | 58.5 | 254.0 |
| **Processed culinary ingredients -G2 Lower** | **Spain** | 40,621 | **44.0** | 21.5 | 0 | 28.5 | 40.8 | 55.8 | 296.5 |
| **Processed culinary ingredients -G2 Middle** | **Spain** | 40,621 | **43.2** | 21.4 | 0 | 27.7 | 40.0 | 54.9 | 296.2 |
| **Processed culinary ingredients -G2 Upper** | **Spain** | 40,621 | **43.0** | 21.4 | 0 | 27.5 | 39.8 | 54.7 | 296.2 |
| **Processed culinary ingredients -G2 Lower** | **United Kingdom** | 81,097 | **24.5** | 19.4 | 0 | 10.6 | 18.3 | 33.6 | 232.4 |
| **Processed culinary ingredients -G2 Middle** | **United Kingdom** | 81,097 | **15.7** | 16.7 | 0 | 4.4 | 9.1 | 21.4 | 213.0 |
| **Processed culinary ingredients -G2 Upper** | **United Kingdom** | 81,097 | **14.2** | 16.7 | 0 | 2.9 | 7.2 | 19.6 | 209.4 |
| **Processed culinary ingredients -G2 Lower** | **The Netherlands** | 39,036 | **26.5** | 23.9 | 0.02 | 10.2 | 17.8 | 35.4 | 329.7 |
| **Processed culinary ingredients -G2 Middle** | **The Netherlands** | 39,036 | **23.4** | 23.0 | 0.02 | 7.7 | 14.9 | 31.9 | 318.1 |
| **Processed culinary ingredients -G2 Upper** | **The Netherlands** | 39,036 | **23.3** | 23.0 | 0.02 | 7.6 | 14.8 | 31.7 | 317.9 |
| **Processed culinary ingredients -G2 Lower** | **Germany** | 52,013 | **31.4** | 24.0 | 0.2 | 15.2 | 25.2 | 40.6 | 500.5 |
| **Processed culinary ingredients -G2 Middle** | **Germany** | 52,013 | **28.2** | 23.5 | 0.2 | 12.4 | 21.9 | 36.8 | 497.6 |
| **Processed culinary ingredients -G2 Upper** | **Germany** | 52,013 | **25.8** | 23.1 | 0 | 10.2 | 19.3 | 34.0 | 493.8 |
| **Processed culinary ingredients -G2 Lower** | **Sweden** | 52,750 | **29.2** | 25.5 | 0 | 11.9 | 21.8 | 38.0 | 317.2 |
| **Processed culinary ingredients -G2 Middle** | **Sweden** | 52,750 | **27.7** | 25.3 | 0 | 10.5 | 20.2 | 36.1 | 314.0 |
| **Processed culinary ingredients -G2 Upper** | **Sweden** | 52,750 | **24.9** | 24.7 | 0 | 8.1 | 17.4 | 32.8 | 292.1 |
| **Processed culinary ingredients -G2 Lower** | **Denmark** | 55,860 | **21.1** | 14.4 | 0.005 | 11.0 | 17.1 | 27.4 | 181.1 |
| **Processed culinary ingredients -G2 Middle** | **Denmark** | 55,860 | **16.5** | 13.6 | 0 | 7.0 | 12.2 | 22.1 | 171.7 |
| **Processed culinary ingredients -G2 Upper** | **Denmark** | 55,860 | **12.9** | 12.9 | 0 | 4.0 | 8.1 | 17.9 | 166.6 |
|  |  |  |  |  |  |  |  |  |  |
| **Label** | **Country** | **N** | **Mean** | **Std** | **Min** | **P25** | **P50** | **P75** | **Max** |
| **Processed culinary ingredients -G2 Lower** | **Norway** | 36,448 | **14.2** | 9.1 | 0 | 8.1 | 12.1 | 17.8 | 127.6 |
| **Processed culinary ingredients -G2 Middle** | **Norway** | 36,448 | **12.5** | 8.8 | 0 | 6.7 | 10.3 | 15.8 | 127.6 |
| **Processed culinary ingredients -G2 Upper** | **Norway** | 36,448 | **9.2** | 8.2 | 0 | 3.8 | 6.8 | 12.0 | 123.1 |
| **Processed foods -G3 Lower** | **France** | 73,035 | **351.0** | 196.3 | 0.9 | 217.0 | 312.5 | 440.4 | 2,473.8 |
| **Processed foods -G3 Middle** | **France** | 73,035 | **356.3** | 196.9 | 1.2 | 221.9 | 318.0 | 446.5 | 2,474.3 |
| **Processed foods -G3 Upper** | **France** | 73,035 | **278.9** | 178.5 | 0 | 159.8 | 234.9 | 348.8 | 2,339.4 |
| **Processed foods -G3 Lower** | **Italy** | 45,908 | **385.6** | 233.6 | 5.6 | 218.5 | 330.1 | 494.6 | 2,799.3 |
| **Processed foods -G3 Middle** | **Italy** | 45,908 | **482.5** | 255.2 | 15.7 | 298.1 | 429.0 | 612.8 | 2,917.4 |
| **Processed foods -G3 Upper** | **Italy** | 45,908 | **268.8** | 205.8 | 0 | 121.3 | 207.2 | 359.7 | 2,514.5 |
| **Processed foods -G3 Lower** | **Spain** | 40,621 | **351.1** | 319.6 | 0 | 139.9 | 245.3 | 456.8 | 4,117.6 |
| **Processed foods -G3 Middle** | **Spain** | 40,621 | **393.4** | 327.5 | 0 | 175.5 | 288.9 | 505.8 | 4,168.7 |
| **Processed foods -G3 Upper** | **Spain** | 40,621 | **298.4** | 293.0 | 0 | 109.7 | 188.8 | 385.3 | 3,943.5 |
| **Processed foods -G3 Lower** | **United Kingdom** | 81,097 | **274.3** | 239.4 | 0 | 140.0 | 213.6 | 323.6 | 2,758.6 |
| **Processed foods -G3 Middle** | **United Kingdom** | 81,097 | **224.9** | 227.9 | 0 | 100.9 | 163.0 | 261.6 | 2,572.4 |
| **Processed foods -G3 Upper** | **United Kingdom** | 81,097 | **256.0** | 232.7 | 0 | 128.5 | 192.7 | 297.0 | 2,607.2 |
| **Processed foods -G3 Lower** | **The Netherlands** | 39,036 | **339.8** | 271.2 | 4.2 | 190.0 | 263.4 | 389.4 | 6,188.2 |
| **Processed foods -G3 Middle** | **The Netherlands** | 39,036 | **325.0** | 267.9 | 6.6 | 179.1 | 249.0 | 370.5 | 6,187.9 |
| **Processed foods -G3 Upper** | **The Netherlands** | 39,036 | **211.0** | 247.8 | 1.4 | 86.6 | 132.3 | 233.2 | 6,114.3 |
| **Processed foods -G3 Lower** | **Germany** | 52,013 | **503.5** | 405.2 | 4.6 | 260.5 | 381.1 | 610.3 | 7,598.9 |
| **Processed foods -G3 Middle** | **Germany** | 52,013 | **523.1** | 406.2 | 5.4 | 278.5 | 401.0 | 631.7 | 7,622.9 |
| **Processed foods -G3 Upper** | **Germany** | 52,013 | **379.2** | 393.3 | 5.2 | 147.3 | 247.1 | 466.9 | 7,477.2 |
| **Processed foods -G3 Lower** | **Sweden** | 52,750 | **314.7** | 217.0 | 4.0 | 168.6 | 261.1 | 400.2 | 4,682.0 |
| **Processed foods -G3 Middle** | **Sweden** | 52,750 | **310.8** | 215.9 | 0.5 | 164.9 | 256.9 | 397.0 | 4,676.5 |
| **Processed foods -G3 Upper** | **Sweden** | 52,750 | **201.2** | 196.9 | 0 | 69.2 | 144.2 | 271.7 | 4,595.4 |
| **Processed foods -G3 Lower** | **Denmark** | 55,860 | **561.7** | 436.7 | 17.0 | 294.6 | 437.4 | 650.4 | 4,912.5 |
| **Processed foods -G3 Middle** | **Denmark** | 55,860 | **452.9** | 425.4 | 8.4 | 197.6 | 323.1 | 522.9 | 4,841.8 |
| **Processed foods -G3 Upper** | **Denmark** | 55,860 | **429.0** | 426.5 | 1.8 | 171.7 | 294.7 | 500.4 | 4,837.7 |
|  |  |  |  |  |  |  |  |  |  |
| **Label** | **Country** | **N** | **Mean** | **Std** | **Min** | **P25** | **P50** | **P75** | **Max** |
| **Processed foods -G3 Lower** | **Norway** | 36,448 | **240.0** | 98.2 | 2.8 | 170.9 | 232.4 | 295.7 | 894.2 |
| **Processed foods -G3 Middle** | **Norway** | 36,448 | **141.0** | 74.5 | 0 | 92.4 | 129.3 | 171.1 | 725.5 |
| **Processed foods -G3 Upper** | **Norway** | 36,448 | **111.8** | 73.6 | 0 | 64.1 | 96.6 | 138.6 | 739.9 |
| **Processed foods -G3 excluding alcohol -Lower** | **France** | 73,035 | **237.6** | 110.4 | 0.9 | 159.0 | 223.6 | 299.3 | 1,201.6 |
| **Processed foods -G3 excluding alcohol -Middle** | **France** | 73,035 | **242.8** | 111.5 | 0.9 | 163.4 | 229.2 | 305.6 | 1,202.3 |
| **Processed foods -G3 excluding alcohol -Upper** | **France** | 73,035 | **165.4** | 79.0 | 0 | 111.5 | 153.0 | 203.8 | 883.8 |
| **Processed foods -G3 excluding alcohol -Lower** | **Italy** | 45,908 | **240.8** | 109.5 | 5.6 | 163.1 | 224.8 | 301.5 | 1,195.4 |
| **Processed foods -G3 excluding alcohol -Middle** | **Italy** | 45,908 | **337.7** | 146.2 | 15.7 | 233.7 | 315.9 | 414.8 | 1,581.4 |
| **Processed foods -G3 excluding alcohol -Upper** | **Italy** | 45,908 | **124.0** | 69.3 | 0 | 76.8 | 111.1 | 155.6 | 1,083.0 |
| **Processed foods -G3 excluding alcohol -Lower** | **Spain** | 40,621 | **187.2** | 106.8 | 0 | 110.4 | 170.5 | 246.2 | 1,048.6 |
| **Processed foods -G3 excluding alcohol -Middle** | **Spain** | 40,621 | **229.6** | 119.8 | 0 | 143.6 | 212.2 | 296.5 | 1,076.9 |
| **Processed foods -G3 excluding alcohol -Upper** | **Spain** | 40,621 | **134.6** | 67.7 | 0 | 86.9 | 122.9 | 169.4 | 739.1 |
| **Processed foods -G3 excluding alcohol -Lower** | **United Kingdom** | 81,097 | **144.7** | 73.5 | 0 | 89.7 | 132.6 | 188.5 | 867.8 |
| **Processed foods -G3 excluding alcohol -Middle** | **United Kingdom** | 81,097 | **95.7** | 55.9 | 0 | 57.2 | 83.4 | 121.1 | 804.9 |
| **Processed foods -G3 excluding alcohol -Upper** | **United Kingdom** | 81,097 | **126.8** | 61.6 | 0 | 85.2 | 116.4 | 155.7 | 1,026.5 |
| **Processed foods -G3 excluding alcohol -Lower** | **The Netherlands** | 39,036 | **218.0** | 91.0 | 0.3 | 155.4 | 204.2 | 265.4 | 1,080.7 |
| **Processed foods -G3 excluding alcohol -Middle** | **The Netherlands** | 39,036 | **203.2** | 87.4 | 0.3 | 143.1 | 190.0 | 247.8 | 1,068.8 |
| **Processed foods -G3 excluding alcohol -Upper** | **The Netherlands** | 39,036 | **89.2** | 43.6 | 1.4 | 60.0 | 81.4 | 109.2 | 811.2 |
| **Processed foods -G3 excluding alcohol -Lower** | **Germany** | 52,013 | **238.1** | 103.0 | 0 | 164.7 | 225.7 | 295.5 | 1,441.5 |
| **Processed foods -G3 excluding alcohol -Middle** | **Germany** | 52,013 | **258.1** | 105.7 | 0 | 183.1 | 245.2 | 317.0 | 1,451.4 |
| **Processed foods -G3 excluding alcohol -Upper** | **Germany** | 52,013 | **114.2** | 59.0 | 0 | 74.9 | 102.9 | 140.4 | 1,388.4 |
| **Processed foods -G3 excluding alcohol -Lower** | **Sweden** | 52,750 | **180.5** | 90.6 | 0 | 116.5 | 163.0 | 226.3 | 1,296.6 |
| **Processed foods -G3 excluding alcohol -Middle** | **Sweden** | 52,750 | **176.6** | 90.9 | 0 | 112.5 | 158.9 | 222.0 | 1,296.6 |
| **Processed foods -G3 excluding alcohol -Upper** | **Sweden** | 52,750 | **67.0** | 50.6 | 0 | 29.1 | 53.8 | 91.7 | 1,073.9 |
| **Processed foods -G3 excluding alcohol -Lower** | **Denmark** | 55,860 | **235.4** | 91.9 | 2.5 | 169.1 | 224.8 | 288.7 | 1,077.7 |
| **Processed foods -G3 excluding alcohol -Middle** | **Denmark** | 55,860 | **126.6** | 67.6 | 0 | 81.3 | 112.4 | 154.3 | 1,161.1 |
| **Processed foods -G3 excluding alcohol -Upper** | **Denmark** | 55,860 | **102.7** | 56.9 | 0 | 65.1 | 91.8 | 126.6 | 995.6 |
|  |  |  |  |  |  |  |  |  |  |
| **Label** | **Country** | **N** | **Mean** | **Std** | **Min** | **P25** | **P50** | **P75** | **Max** |
| **Processed foods -G3 excluding alcohol -Lower** | **Norway** | 36,448 | **189.3** | 75.6 | 2.2 | 139.9 | 175.9 | 247.0 | 509.7 |
| **Processed foods -G3 excluding alcohol -Middle** | **Norway** | 36,448 | **90.3** | 41.3 | 0 | 60.0 | 85.7 | 116.3 | 362.5 |
| **Processed foods -G3 excluding alcohol -Upper** | **Norway** | 36,448 | **61.2** | 31.4 | 0 | 38.1 | 56.3 | 78.9 | 291.1 |
| **Ultra-processed foods -G4 Lower** | **France** | 73,035 | **195.0** | 128.7 | 0.3 | 107.7 | 166.8 | 247.5 | 2,155.7 |
| **Ultra-processed foods -G4 Middle** | **France** | 73,035 | **215.0** | 136.1 | 0.3 | 121.6 | 186.6 | 274.3 | 2,188.2 |
| **Ultra-processed foods -G4 Upper** | **France** | 73,035 | **447.8** | 196.9 | 0.7 | 306.7 | 424.5 | 562.5 | 2,330.5 |
| **Ultra-processed foods -G4 Lower** | **Italy** | 45,908 | **194.7** | 146.4 | 0 | 100.9 | 157.7 | 244.5 | 3,242.8 |
| **Ultra-processed foods -G4 Middle** | **Italy** | 45,908 | **194.5** | 146.5 | 0 | 100.6 | 157.4 | 244.3 | 3,242.8 |
| **Ultra-processed foods -G4 Upper** | **Italy** | 45,908 | **479.2** | 210.1 | 22.9 | 332.3 | 445.8 | 584.7 | 3,608.1 |
| **Ultra-processed foods -G4 Lower** | **Spain** | 40,621 | **155.4** | 140.7 | 0 | 66.8 | 118.0 | 198.9 | 2,230.8 |
| **Ultra-processed foods -G4 Middle** | **Spain** | 40,621 | **157.0** | 140.9 | 0 | 68.3 | 119.9 | 200.8 | 2,230.8 |
| **Ultra-processed foods -G4 Upper** | **Spain** | 40,621 | **371.9** | 192.3 | 0 | 240.8 | 338.3 | 462.9 | 2,600.6 |
| **Ultra-processed foods -G4 Lower** | **United Kingdom** | 81,097 | **399.8** | 277.3 | 0 | 222.3 | 325.6 | 483.9 | 4,686.9 |
| **Ultra-processed foods -G4 Middle** | **United Kingdom** | 81,097 | **518.2** | 293.3 | 0 | 327.6 | 448.4 | 622.2 | 4,976.4 |
| **Ultra-processed foods -G4 Upper** | **United Kingdom** | 81,097 | **572.3** | 301.4 | 0 | 373.9 | 505.1 | 688.1 | 5,066.8 |
| **Ultra-processed foods -G4 Lower** | **The Netherlands** | 39,036 | **399.6** | 222.0 | 24.0 | 250.6 | 350.3 | 491.7 | 4,754.1 |
| **Ultra-processed foods -G4 Middle** | **The Netherlands** | 39,036 | **442.5** | 236.6 | 26.4 | 282.7 | 390.3 | 543.9 | 4,874.2 |
| **Ultra-processed foods -G4 Upper** | **The Netherlands** | 39,036 | **629.9** | 275.6 | 60.4 | 441.1 | 577.9 | 759.3 | 5,281.1 |
| **Ultra-processed foods -G4 Lower** | **Germany** | 52,013 | **452.8** | 294.3 | 0.7 | 260.2 | 381.2 | 561.7 | 4,666.1 |
| **Ultra-processed foods -G4 Middle** | **Germany** | 52,013 | **463.3** | 295.9 | 0.7 | 269.3 | 392.0 | 573.5 | 4,664.6 |
| **Ultra-processed foods -G4 Upper** | **Germany** | 52,013 | **655.9** | 322.8 | 1.6 | 441.9 | 588.9 | 790.3 | 5,010.1 |
| **Ultra-processed foods -G4 Lower** | **Sweden** | 52,750 | **298.5** | 196.7 | 5.9 | 168.3 | 249.2 | 371.5 | 3,158.6 |
| **Ultra-processed foods -G4 Middle** | **Sweden** | 52,750 | **338.9** | 204.1 | 7.1 | 203.3 | 290.8 | 419.1 | 3,157.7 |
| **Ultra-processed foods -G4 Upper** | **Sweden** | 52,750 | **487.5** | 236.6 | 50.0 | 325.5 | 439.8 | 594.4 | 3,375.9 |
| **Ultra-processed foods -G4 Lower** | **Denmark** | 55,860 | **299.1** | 277.1 | 0 | 129.2 | 210.8 | 350.1 | 3,531.3 |
| **Ultra-processed foods -G4 Middle** | **Denmark** | 55,860 | **482.1** | 295.1 | 12.0 | 297.0 | 404.8 | 563.5 | 3,699.1 |
| **Ultra-processed foods -G4 Upper** | **Denmark** | 55,860 | **566.7** | 308.0 | 19.5 | 367.8 | 491.0 | 665.8 | 3,763.0 |
|  |  |  |  |  |  |  |  |  |  |
| **Label** | **Country** | **N** | **Mean** | **Std** | **Min** | **P25** | **P50** | **P75** | **Max** |
| **Ultra-processed foods -G4 Lower** | **Norway** | 36,448 | **236.8** | 145.1 | 1.6 | 143.7 | 203.2 | 279.9 | 1,530.5 |
| **Ultra-processed foods -G4 Middle** | **Norway** | 36,448 | **386.2** | 163.3 | 11.3 | 277.3 | 359.7 | 457.1 | 1,776.1 |
| **Ultra-processed foods -G4 Upper** | **Norway** | 36,448 | **470.0** | 176.8 | 22.9 | 349.5 | 444.9 | 558.6 | 1,883.9 |
| **Ultra-processed foods -G4 excluding alcohol -Lower** | **France** | 73,035 | **181.3** | 124.8 | 0.3 | 97.5 | 152.9 | 230.3 | 2,155.7 |
| **Ultra-processed foods -G4 excluding alcohol -Middle** | **France** | 73,035 | **201.4** | 132.2 | 0.3 | 111.1 | 172.9 | 256.8 | 2,188.2 |
| **Ultra-processed foods -G4 excluding alcohol -Upper** | **France** | 73,035 | **434.2** | 195.4 | 0.7 | 293.6 | 410.7 | 547.6 | 2,330.5 |
| **Ultra-processed foods -G4 excluding alcohol -Lower** | **Italy** | 45,908 | **189.8** | 145.1 | 0 | 97.1 | 152.8 | 238.0 | 3,122.8 |
| **Ultra-processed foods -G4 excluding alcohol -Middle** | **Italy** | 45,908 | **189.6** | 145.1 | 0 | 96.9 | 152.5 | 237.9 | 3,122.8 |
| **Ultra-processed foods -G4 excluding alcohol -Upper** | **Italy** | 45,908 | **474.3** | 208.9 | 22.9 | 328.3 | 441.1 | 578.6 | 3,488.1 |
| **Ultra-processed foods -G4 excluding alcohol -Lower** | **Spain** | 40,621 | **149.9** | 137.7 | 0 | 62.9 | 112.8 | 192.4 | 2,230.8 |
| **Ultra-processed foods -G4 excluding alcohol -Middle** | **Spain** | 40,621 | **151.6** | 137.9 | 0 | 64.5 | 114.7 | 194.4 | 2,230.8 |
| **Ultra-processed foods -G4 excluding alcohol -Upper** | **Spain** | 40,621 | **366.4** | 188.3 | 0 | 238.3 | 333.9 | 455.4 | 2,600.6 |
| **Ultra-processed foods -G4 excluding alcohol -Lower** | **United Kingdom** | 81,097 | **392.8** | 276.8 | 0 | 215.3 | 317.7 | 476.7 | 4,686.1 |
| **Ultra-processed foods -G4 excluding alcohol -Middle** | **United Kingdom** | 81,097 | **511.2** | 292.8 | 0 | 321.5 | 441.1 | 614.3 | 4,975.7 |
| **Ultra-processed foods -G4 excluding alcohol -Upper** | **United Kingdom** | 81,097 | **565.3** | 300.9 | 0 | 367.1 | 498.1 | 681.0 | 5,066.0 |
| **Ultra-processed foods -G4 excluding alcohol -Lower** | **The Netherlands** | 39,036 | **381.3** | 221.0 | 19.3 | 232.3 | 330.0 | 470.9 | 4,754.1 |
| **Ultra-processed foods -G4 excluding alcohol -Middle** | **The Netherlands** | 39,036 | **424.1** | 236.2 | 26.4 | 264.0 | 370.0 | 523.8 | 4,874.2 |
| **Ultra-processed foods -G4 excluding alcohol -Upper** | **The Netherlands** | 39,036 | **611.6** | 276.4 | 46.1 | 420.9 | 557.9 | 740.8 | 5,281.1 |
| **Ultra-processed foods -G4 excluding alcohol -Lower** | **Germany** | 52,013 | **449.0** | 293.6 | 0.7 | 257.0 | 376.9 | 557.4 | 4,665.9 |
| **Ultra-processed foods -G4 excluding alcohol -Middle** | **Germany** | 52,013 | **459.6** | 295.2 | 0.7 | 266.0 | 387.9 | 568.9 | 4,664.4 |
| **Ultra-processed foods -G4 excluding alcohol -Upper** | **Germany** | 52,013 | **652.1** | 322.1 | 1.6 | 438.4 | 585.2 | 785.7 | 5,010.1 |
| **Ultra-processed foods -G4 excluding alcohol -Lower** | **Sweden** | 52,750 | **288.3** | 194.6 | 5.9 | 160.5 | 238.0 | 359.0 | 3,158.6 |
| **Ultra-processed foods -G4 excluding alcohol -Middle** | **Sweden** | 52,750 | **328.7** | 202.0 | 7.1 | 194.9 | 279.6 | 406.8 | 3,157.7 |
| **Ultra-processed foods -G4 excluding alcohol -Upper** | **Sweden** | 52,750 | **477.3** | 235.1 | 40.5 | 316.9 | 429.2 | 582.5 | 3,375.9 |
| **Ultra-processed foods -G4 excluding alcohol -Lower** | **Denmark** | 55,860 | **289.3** | 276.8 | 0 | 119.9 | 199.4 | 338.9 | 3,528.8 |
| **Ultra-processed foods -G4 excluding alcohol -Middle** | **Denmark** | 55,860 | **472.3** | 294.9 | 11.0 | 287.4 | 394.4 | 553.1 | 3,696.6 |
| **Ultra-processed foods -G4 excluding alcohol -Upper** | **Denmark** | 55,860 | **556.9** | 307.7 | 18.5 | 358.4 | 480.3 | 655.2 | 3,760.5 |
|  |  |  |  |  |  |  |  |  |  |
| **Label** | **Country** | **N** | **Mean** | **Std** | **Min** | **P25** | **P50** | **P75** | **Max** |
| **Ultra-processed foods -G4 excluding alcohol -Lower** | **Norway** | 36,448 | **236.0** | 145.0 | 1.6 | 143.0 | 202.5 | 279.0 | 1,530.5 |
| **Ultra-processed foods -G4 excluding alcohol -Middle** | **Norway** | 36,448 | **385.4** | 163.3 | 11.3 | 276.8 | 358.9 | 456.3 | 1,776.1 |
| **Ultra-processed foods -G4 excluding alcohol -Upper** | **Norway** | 36,448 | **469.3** | 176.7 | 22.9 | 348.7 | 444.1 | 557.9 | 1,883.9 |
| **kcal/day** |  |  |  |  |  |  |  |  |  |
| **Energy: Unprocessed or minimally processed foods -G1 Lower** | **France** | 73,035 | **875.9** | 285.2 | 54.5 | 680.6 | 840.3 | 1,031.3 | 3,418.0 |
| **Energy: Unprocessed or minimally processed foods -G1 Middle** | **France** | 73,035 | **855.2** | 283.4 | 51.9 | 660.9 | 819.5 | 1,009.0 | 3,418.0 |
| **Energy: Unprocessed or minimally processed foods -G1 Upper** | **France** | 73,035 | **754.5** | 267.8 | 13.2 | 571.3 | 718.2 | 895.2 | 3,274.7 |
| **Energy: Unprocessed or minimally processed foods -G1 Lower** | **Italy** | 45,908 | **845.8** | 282.2 | 44.1 | 645.3 | 812.1 | 1,007.1 | 2,776.4 |
| **Energy: Unprocessed or minimally processed foods -G1 Middle** | **Italy** | 45,908 | **787.3** | 262.6 | 42.2 | 598.9 | 756.9 | 940.8 | 2,549.4 |
| **Energy: Unprocessed or minimally processed foods -G1 Upper** | **Italy** | 45,908 | **717.3** | 245.6 | 39.8 | 544.0 | 688.4 | 855.9 | 2,491.1 |
| **Energy: Unprocessed or minimally processed foods -G1 Lower** | **Spain** | 40,621 | **956.3** | 276.8 | 82.8 | 763.9 | 925.7 | 1,114.2 | 3,122.7 |
| **Energy: Unprocessed or minimally processed foods -G1 Middle** | **Spain** | 40,621 | **915.9** | 265.3 | 82.2 | 731.9 | 887.1 | 1,067.1 | 3,070.7 |
| **Energy: Unprocessed or minimally processed foods -G1 Upper** | **Spain** | 40,621 | **776.0** | 232.7 | 7.1 | 615.9 | 749.9 | 906.0 | 2,825.5 |
| **Energy: Unprocessed or minimally processed foods -G1 Lower** | **United Kingdom** | 81,097 | **854.7** | 268.0 | 117.2 | 670.0 | 824.2 | 1,004.2 | 3,288.3 |
| **Energy: Unprocessed or minimally processed foods -G1 Middle** | **United Kingdom** | 81,097 | **769.1** | 252.8 | 107.1 | 595.8 | 739.7 | 906.2 | 3,217.9 |
| **Energy: Unprocessed or minimally processed foods -G1 Upper** | **United Kingdom** | 81,097 | **698.4** | 233.1 | 56.3 | 538.7 | 673.5 | 827.7 | 2,816.3 |
| **Energy: Unprocessed or minimally processed foods -G1 Lower** | **The Netherlands** | 39,036 | **748.7** | 220.7 | 67.0 | 598.2 | 727.5 | 873.2 | 2,664.0 |
| **Energy: Unprocessed or minimally processed foods -G1 Middle** | **The Netherlands** | 39,036 | **727.1** | 218.1 | 67.0 | 577.6 | 705.9 | 850.0 | 2,651.4 |
| **Energy: Unprocessed or minimally processed foods -G1 Upper** | **The Netherlands** | 39,036 | **681.6** | 213.4 | 65.9 | 535.2 | 660.5 | 801.6 | 2,384.3 |
| **Energy: Unprocessed or minimally processed foods -G1 Lower** | **Germany** | 52,013 | **551.1** | 197.0 | 19.9 | 418.7 | 520.0 | 646.9 | 2,610.3 |
| **Energy: Unprocessed or minimally processed foods -G1 Middle** | **Germany** | 52,013 | **515.7** | 190.7 | 19.9 | 388.0 | 484.4 | 606.4 | 2,415.5 |
| **Energy: Unprocessed or minimally processed foods -G1 Upper** | **Germany** | 52,013 | **465.3** | 178.7 | 19.8 | 345.8 | 435.2 | 549.1 | 2,310.3 |
| **Energy: Unprocessed or minimally processed foods -G1 Lower** | **Sweden** | 52,750 | **793.3** | 282.8 | 31.5 | 599.1 | 750.6 | 938.7 | 4,052.2 |
| **Energy: Unprocessed or minimally processed foods -G1 Middle** | **Sweden** | 52,750 | **745.7** | 272.9 | 31.5 | 557.6 | 706.3 | 886.5 | 4,019.9 |
| **Energy: Unprocessed or minimally processed foods -G1 Upper** | **Sweden** | 52,750 | **698.9** | 262.4 | 25.8 | 518.6 | 662.6 | 835.9 | 3,991.1 |
| **Energy: Unprocessed or minimally processed foods -G1 Lower** | **Denmark** | 55,860 | **822.8** | 265.6 | 94.2 | 633.8 | 791.7 | 977.2 | 2,661.3 |
| **Energy: Unprocessed or minimally processed foods -G1 Middle** | **Denmark** | 55,860 | **767.4** | 251.5 | 88.2 | 589.3 | 737.9 | 912.7 | 2,612.2 |
| **Energy: Unprocessed or minimally processed foods -G1 Upper** | **Denmark** | 55,860 | **697.8** | 239.1 | 73.1 | 527.9 | 668.7 | 836.7 | 2,571.2 |
|  |  |  |  |  |  |  |  |  |  |
| **Label** | **Country** | **N** | **Mean** | **Std** | **Min** | **P25** | **P50** | **P75** | **Max** |
| **Energy: Unprocessed or minimally processed foods -G1 Lower** | **Norway** | 36,448 | **674.0** | 199.5 | 5.1 | 534.5 | 656.5 | 796.0 | 1,867.8 |
| **Energy: Unprocessed or minimally processed foods -G1 Middle** | **Norway** | 36,448 | **623.0** | 190.4 | 5.1 | 489.4 | 604.9 | 738.1 | 1,808.0 |
| **Energy: Unprocessed or minimally processed foods -G1 Upper** | **Norway** | 36,448 | **556.9** | 181.8 | 5.1 | 429.2 | 538.1 | 664.1 | 1,801.5 |
| **Energy: Processed culinary ingredients -G2 Lower** | **France** | 73,035 | **233.8** | 105.6 | 0 | 159.2 | 219.6 | 291.2 | 1,280.2 |
| **Energy: Processed culinary ingredients -G2 Middle** | **France** | 73,035 | **225.5** | 104.8 | 0 | 151.5 | 211.4 | 282.5 | 1,276.9 |
| **Energy: Processed culinary ingredients -G2 Upper** | **France** | 73,035 | **214.5** | 104.4 | 0 | 141.0 | 201.1 | 271.7 | 1,243.6 |
| **Energy: Processed culinary ingredients -G2 Lower** | **Italy** | 45,908 | **361.2** | 138.3 | 0 | 265.4 | 343.4 | 435.5 | 1,412.5 |
| **Energy: Processed culinary ingredients -G2 Middle** | **Italy** | 45,908 | **351.6** | 136.7 | 0 | 256.6 | 334.3 | 425.2 | 1,384.3 |
| **Energy: Processed culinary ingredients -G2 Upper** | **Italy** | 45,908 | **341.7** | 134.5 | 0 | 248.6 | 323.9 | 413.2 | 1,328.1 |
| **Energy: Processed culinary ingredients -G2 Lower** | **Spain** | 40,621 | **307.4** | 142.0 | 0 | 205.6 | 284.5 | 386.0 | 2,042.0 |
| **Energy: Processed culinary ingredients -G2 Middle** | **Spain** | 40,621 | **306.2** | 142.0 | 0 | 204.4 | 283.2 | 384.4 | 2,041.9 |
| **Energy: Processed culinary ingredients -G2 Upper** | **Spain** | 40,621 | **305.5** | 142.0 | 0 | 203.6 | 282.5 | 383.8 | 2,035.5 |
| **Energy: Processed culinary ingredients -G2 Lower** | **United Kingdom** | 81,097 | **110.4** | 98.3 | 0 | 43.3 | 78.7 | 143.8 | 979.2 |
| **Energy: Processed culinary ingredients -G2 Middle** | **United Kingdom** | 81,097 | **67.9** | 87.9 | 0 | 11.7 | 30.3 | 88.6 | 880.3 |
| **Energy: Processed culinary ingredients -G2 Upper** | **United Kingdom** | 81,097 | **65.0** | 87.8 | 0 | 8.3 | 26.1 | 85.4 | 880.0 |
| **Energy: Processed culinary ingredients -G2 Lower** | **The Netherlands** | 39,036 | **147.7** | 114.7 | 0.2 | 66.0 | 114.7 | 198.3 | 1,715.0 |
| **Energy: Processed culinary ingredients -G2 Middle** | **The Netherlands** | 39,036 | **122.4** | 105.3 | 0.2 | 46.2 | 90.8 | 168.6 | 1,542.0 |
| **Energy: Processed culinary ingredients -G2 Upper** | **The Netherlands** | 39,036 | **122.1** | 105.3 | 0.2 | 46.0 | 90.5 | 168.3 | 1,541.9 |
| **Energy: Processed culinary ingredients -G2 Lower** | **Germany** | 52,013 | **170.9** | 121.2 | 0.3 | 85.1 | 140.8 | 223.6 | 1,998.9 |
| **Energy: Processed culinary ingredients -G2 Middle** | **Germany** | 52,013 | **159.3** | 120.0 | 0.1 | 73.7 | 129.0 | 211.1 | 1,990.4 |
| **Energy: Processed culinary ingredients -G2 Upper** | **Germany** | 52,013 | **150.2** | 118.3 | 0 | 65.8 | 119.5 | 200.4 | 1,973.0 |
| **Energy: Processed culinary ingredients -G2 Lower** | **Sweden** | 52,750 | **115.4** | 118.2 | 0 | 40.0 | 79.6 | 145.8 | 1,446.9 |
| **Energy: Processed culinary ingredients -G2 Middle** | **Sweden** | 52,750 | **111.1** | 117.8 | 0 | 36.2 | 75.0 | 140.6 | 1,440.1 |
| **Energy: Processed culinary ingredients -G2 Upper** | **Sweden** | 52,750 | **102.3** | 114.5 | 0 | 29.7 | 66.9 | 129.7 | 1,431.7 |
| **Energy: Processed culinary ingredients -G2 Lower** | **Denmark** | 55,860 | **83.9** | 77.8 | 0 | 28.5 | 60.2 | 110.2 | 857.6 |
| **Energy: Processed culinary ingredients -G2 Middle** | **Denmark** | 55,860 | **70.6** | 76.2 | 0 | 16.2 | 45.2 | 94.6 | 813.7 |
| **Energy: Processed culinary ingredients -G2 Upper** | **Denmark** | 55,860 | **60.0** | 74.6 | 0 | 7.6 | 32.8 | 80.4 | 806.9 |
|  |  |  |  |  |  |  |  |  |  |
| **Label** | **Country** | **N** | **Mean** | **Std** | **Min** | **P25** | **P50** | **P75** | **Max** |
| **Energy: Processed culinary ingredients -G2 Lower** | **Norway** | 36,448 | **74.6** | 56.4 | 0 | 38.7 | 58.4 | 92.4 | 873.6 |
| **Energy: Processed culinary ingredients -G2 Middle** | **Norway** | 36,448 | **67.4** | 55.6 | 0 | 32.3 | 50.5 | 83.6 | 873.5 |
| **Energy: Processed culinary ingredients -G2 Upper** | **Norway** | 36,448 | **52.7** | 53.6 | 0 | 20.0 | 34.2 | 65.5 | 839.4 |
| **Energy: Processed foods -G3 Lower** | **France** | 73,035 | **660.5** | 319.1 | 0.4 | 434.0 | 618.2 | 839.5 | 3,010.1 |
| **Energy: Processed foods -G3 Middle** | **France** | 73,035 | **664.8** | 319.7 | 0.4 | 437.7 | 622.6 | 844.1 | 3,004.8 |
| **Energy: Processed foods -G3 Upper** | **France** | 73,035 | **424.3** | 235.4 | 0 | 261.4 | 379.1 | 536.3 | 2,547.0 |
| **Energy: Processed foods -G3 Lower** | **Italy** | 45,908 | **734.1** | 341.4 | 9.2 | 487.0 | 683.1 | 929.9 | 3,450.5 |
| **Energy: Processed foods -G3 Middle** | **Italy** | 45,908 | **803.0** | 353.0 | 27.6 | 548.1 | 750.2 | 1,005.7 | 3,973.5 |
| **Energy: Processed foods -G3 Upper** | **Italy** | 45,908 | **387.5** | 225.5 | 0 | 226.5 | 342.1 | 500.5 | 2,432.6 |
| **Energy: Processed foods -G3 Lower** | **Spain** | 40,621 | **610.6** | 387.0 | 0 | 330.3 | 530.4 | 811.4 | 3,027.8 |
| **Energy: Processed foods -G3 Middle** | **Spain** | 40,621 | **649.0** | 398.0 | 0 | 360.4 | 569.1 | 858.6 | 3,054.0 |
| **Energy: Processed foods -G3 Upper** | **Spain** | 40,621 | **351.8** | 264.7 | 0 | 162.0 | 277.9 | 467.0 | 2,356.6 |
| **Energy: Processed foods -G3 Lower** | **United Kingdom** | 81,097 | **436.7** | 230.8 | 0 | 269.6 | 396.2 | 561.2 | 2,372.9 |
| **Energy: Processed foods -G3 Middle** | **United Kingdom** | 81,097 | **304.7** | 181.0 | 0 | 178.5 | 264.4 | 387.2 | 1,844.2 |
| **Energy: Processed foods -G3 Upper** | **United Kingdom** | 81,097 | **281.8** | 169.3 | 0 | 170.1 | 243.5 | 346.6 | 2,385.1 |
| **Energy: Processed foods -G3 Lower** | **The Netherlands** | 39,036 | **614.9** | 272.7 | 10.5 | 428.5 | 565.9 | 746.3 | 3,096.9 |
| **Energy: Processed foods -G3 Middle** | **The Netherlands** | 39,036 | **599.8** | 268.2 | 10.5 | 416.8 | 551.7 | 729.0 | 3,073.7 |
| **Energy: Processed foods -G3 Upper** | **The Netherlands** | 39,036 | **276.0** | 164.2 | 1.2 | 165.9 | 239.8 | 343.9 | 2,824.9 |
| **Energy: Processed foods -G3 Lower** | **Germany** | 52,013 | **719.7** | 310.0 | 4.8 | 502.6 | 675.9 | 887.5 | 4,181.9 |
| **Energy: Processed foods -G3 Middle** | **Germany** | 52,013 | **750.9** | 314.4 | 6.0 | 530.2 | 706.8 | 920.9 | 4,203.1 |
| **Energy: Processed foods -G3 Upper** | **Germany** | 52,013 | **347.1** | 211.0 | 5.1 | 204.8 | 300.0 | 436.2 | 3,911.9 |
| **Energy: Processed foods -G3 Lower** | **Sweden** | 52,750 | **522.9** | 257.7 | 11.5 | 339.0 | 476.3 | 656.8 | 2,966.1 |
| **Energy: Processed foods -G3 Middle** | **Sweden** | 52,750 | **508.1** | 251.7 | 1.9 | 328.8 | 462.9 | 639.0 | 2,888.1 |
| **Energy: Processed foods -G3 Upper** | **Sweden** | 52,750 | **208.9** | 145.1 | 0 | 101.8 | 176.6 | 281.0 | 1,970.4 |
| **Energy: Processed foods -G3 Lower** | **Denmark** | 55,860 | **758.6** | 310.5 | 19.9 | 534.9 | 717.4 | 934.6 | 2,830.2 |
| **Energy: Processed foods -G3 Middle** | **Denmark** | 55,860 | **420.4** | 228.2 | 2.0 | 261.8 | 373.1 | 524.2 | 2,131.1 |
| **Energy: Processed foods -G3 Upper** | **Denmark** | 55,860 | **379.8** | 225.2 | 1.0 | 223.2 | 330.5 | 481.8 | 2,082.0 |
|  |  |  |  |  |  |  |  |  |  |
| **Label** | **Country** | **N** | **Mean** | **Std** | **Min** | **P25** | **P50** | **P75** | **Max** |
| **Energy: Processed foods -G3 Lower** | **Norway** | 36,448 | **500.1** | 197.8 | 1.8 | 365.6 | 475.3 | 642.3 | 1,396.8 |
| **Energy: Processed foods -G3 Middle** | **Norway** | 36,448 | **207.6** | 96.8 | 0 | 136.6 | 193.7 | 264.9 | 836.9 |
| **Energy: Processed foods -G3 Upper** | **Norway** | 36,448 | **185.7** | 94.7 | 0 | 115.3 | 170.6 | 241.6 | 844.1 |
| **Energy: Processed foods -G3 excluding alcohol -Lower** | **France** | 73,035 | **576.7** | 290.2 | 0.3 | 371.1 | 537.9 | 735.4 | 2,997.4 |
| **Energy: Processed foods -G3 excluding alcohol -Middle** | **France** | 73,035 | **581.0** | 291.0 | 0.2 | 374.5 | 542.2 | 740.7 | 2,992.1 |
| **Energy: Processed foods -G3 excluding alcohol -Upper** | **France** | 73,035 | **340.6** | 195.3 | 0 | 209.1 | 302.7 | 426.6 | 2,293.1 |
| **Energy: Processed foods -G3 excluding alcohol -Lower** | **Italy** | 45,908 | **622.2** | 285.7 | 9.2 | 419.6 | 580.5 | 780.4 | 3,443.6 |
| **Energy: Processed foods -G3 excluding alcohol -Middle** | **Italy** | 45,908 | **691.1** | 299.8 | 27.6 | 476.6 | 649.5 | 860.0 | 3,966.6 |
| **Energy: Processed foods -G3 excluding alcohol -Upper** | **Italy** | 45,908 | **275.7** | 159.7 | 0 | 166.1 | 246.4 | 346.8 | 1,882.8 |
| **Energy: Processed foods -G3 excluding alcohol -Lower** | **Spain** | 40,621 | **503.3** | 294.2 | 0 | 291.9 | 455.6 | 665.0 | 2,586.7 |
| **Energy: Processed foods -G3 excluding alcohol -Middle** | **Spain** | 40,621 | **541.9** | 306.2 | 0 | 321.2 | 492.5 | 711.6 | 2,588.6 |
| **Energy: Processed foods -G3 excluding alcohol -Upper** | **Spain** | 40,621 | **244.9** | 163.4 | 0 | 131.4 | 204.5 | 314.1 | 1,854.4 |
| **Energy: Processed foods -G3 excluding alcohol -Lower** | **United Kingdom** | 81,097 | **359.7** | 192.6 | 0 | 214.9 | 326.4 | 470.9 | 2,237.4 |
| **Energy: Processed foods -G3 excluding alcohol -Middle** | **United Kingdom** | 81,097 | **228.1** | 141.7 | 0 | 131.7 | 195.8 | 285.8 | 1,663.7 |
| **Energy: Processed foods -G3 excluding alcohol -Upper** | **United Kingdom** | 81,097 | **205.1** | 116.7 | 0 | 129.8 | 182.7 | 251.7 | 1,618.1 |
| **Energy: Processed foods -G3 excluding alcohol -Lower** | **The Netherlands** | 39,036 | **545.8** | 232.3 | 0.5 | 385.9 | 510.6 | 664.0 | 2,563.7 |
| **Energy: Processed foods -G3 excluding alcohol -Middle** | **The Netherlands** | 39,036 | **530.7** | 228.6 | 0.5 | 373.3 | 496.0 | 646.8 | 2,541.8 |
| **Energy: Processed foods -G3 excluding alcohol -Upper** | **The Netherlands** | 39,036 | **206.9** | 106.0 | 1.2 | 133.4 | 188.0 | 258.3 | 1,480.8 |
| **Energy: Processed foods -G3 excluding alcohol -Lower** | **Germany** | 52,013 | **588.7** | 258.7 | 0 | 405.1 | 556.9 | 733.3 | 4,176.4 |
| **Energy: Processed foods -G3 excluding alcohol -Middle** | **Germany** | 52,013 | **620.4** | 264.8 | 0 | 432.0 | 587.4 | 767.8 | 4,197.6 |
| **Energy: Processed foods -G3 excluding alcohol -Upper** | **Germany** | 52,013 | **216.6** | 124.3 | 0 | 134.8 | 192.6 | 267.2 | 3,906.4 |
| **Energy: Processed foods -G3 excluding alcohol -Lower** | **Sweden** | 52,750 | **467.5** | 235.5 | 0 | 302.5 | 423.0 | 586.1 | 2,966.1 |
| **Energy: Processed foods -G3 excluding alcohol -Middle** | **Sweden** | 52,750 | **452.7** | 230.2 | 0 | 291.0 | 409.6 | 568.9 | 2,888.1 |
| **Energy: Processed foods -G3 excluding alcohol -Upper** | **Sweden** | 52,750 | **153.4** | 110.8 | 0 | 74.0 | 126.0 | 208.9 | 1,742.1 |
| **Energy: Processed foods -G3 excluding alcohol -Lower** | **Denmark** | 55,860 | **580.3** | 237.4 | 6.6 | 407.9 | 552.7 | 719.2 | 2,397.7 |
| **Energy: Processed foods -G3 excluding alcohol -Middle** | **Denmark** | 55,860 | **242.1** | 115.3 | 0 | 156.1 | 225.3 | 307.3 | 1,138.5 |
| **Energy: Processed foods -G3 excluding alcohol -Upper** | **Denmark** | 55,860 | **201.5** | 107.3 | 0 | 121.2 | 185.1 | 261.7 | 1,012.3 |
|  |  |  |  |  |  |  |  |  |  |
| **Label** | **Country** | **N** | **Mean** | **Std** | **Min** | **P25** | **P50** | **P75** | **Max** |
| **Energy: Processed foods -G3 excluding alcohol -Lower** | **Norway** | 36,448 | **471.0** | 197.3 | 1.4 | 340.1 | 438.8 | 615.9 | 1,396.8 |
| **Energy: Processed foods -G3 excluding alcohol -Middle** | **Norway** | 36,448 | **178.5** | 94.2 | 0 | 108.2 | 163.3 | 233.0 | 836.9 |
| **Energy: Processed foods -G3 excluding alcohol -Upper** | **Norway** | 36,448 | **156.7** | 88.9 | 0 | 90.1 | 139.2 | 207.4 | 828.9 |
| **Energy: Ultra-processed foods -G4 Lower** | **France** | 73,035 | **404.4** | 227.9 | 1.4 | 243.5 | 362.2 | 517.5 | 2,288.7 |
| **Energy: Ultra-processed foods -G4 Middle** | **France** | 73,035 | **429.0** | 237.3 | 1.4 | 260.9 | 386.0 | 548.8 | 2,317.4 |
| **Energy: Ultra-processed foods -G4 Upper** | **France** | 73,035 | **781.2** | 330.6 | 2.9 | 546.4 | 738.9 | 966.6 | 3,076.9 |
| **Energy: Ultra-processed foods -G4 Lower** | **Italy** | 45,908 | **356.7** | 208.4 | 0 | 210.6 | 317.5 | 457.5 | 2,241.8 |
| **Energy: Ultra-processed foods -G4 Middle** | **Italy** | 45,908 | **356.0** | 208.1 | 0 | 210.2 | 316.8 | 456.7 | 2,241.8 |
| **Energy: Ultra-processed foods -G4 Upper** | **Italy** | 45,908 | **851.3** | 353.3 | 40.3 | 599.5 | 803.3 | 1,050.1 | 3,895.2 |
| **Energy: Ultra-processed foods -G4 Lower** | **Spain** | 40,621 | **345.9** | 239.3 | 0 | 173.6 | 297.7 | 464.5 | 2,732.8 |
| **Energy: Ultra-processed foods -G4 Middle** | **Spain** | 40,621 | **349.1** | 240.0 | 0 | 176.4 | 300.8 | 467.9 | 2,747.6 |
| **Energy: Ultra-processed foods -G4 Upper** | **Spain** | 40,621 | **786.9** | 370.6 | 0 | 522.2 | 733.4 | 994.6 | 3,529.3 |
| **Energy: Ultra-processed foods -G4 Lower** | **United Kingdom** | 81,097 | **693.8** | 339.6 | 0 | 452.3 | 628.9 | 866.9 | 3,825.5 |
| **Energy: Ultra-processed foods -G4 Middle** | **United Kingdom** | 81,097 | **953.9** | 406.9 | 0 | 661.7 | 888.0 | 1,175.1 | 4,482.3 |
| **Energy: Ultra-processed foods -G4 Upper** | **United Kingdom** | 81,097 | **1,050.4** | 436.0 | 0 | 735.3 | 982.9 | 1,293.3 | 4,652.8 |
| **Energy: Ultra-processed foods -G4 Lower** | **The Netherlands** | 39,036 | **671.1** | 266.6 | 46.2 | 483.4 | 635.3 | 815.9 | 3,242.3 |
| **Energy: Ultra-processed foods -G4 Middle** | **The Netherlands** | 39,036 | **733.1** | 292.3 | 50.6 | 527.7 | 691.4 | 888.5 | 3,420.4 |
| **Energy: Ultra-processed foods -G4 Upper** | **The Netherlands** | 39,036 | **1,102.7** | 395.2 | 85.5 | 826.3 | 1,046.0 | 1,310.2 | 4,102.0 |
| **Energy: Ultra-processed foods -G4 Lower** | **Germany** | 52,013 | **707.3** | 358.8 | 0.8 | 456.2 | 641.2 | 884.7 | 4,182.6 |
| **Energy: Ultra-processed foods -G4 Middle** | **Germany** | 52,013 | **723.1** | 362.0 | 0.8 | 469.0 | 656.9 | 902.6 | 4,210.7 |
| **Energy: Ultra-processed foods -G4 Upper** | **Germany** | 52,013 | **1,186.5** | 466.3 | 2.1 | 857.7 | 1,115.2 | 1,436.9 | 4,556.6 |
| **Energy: Ultra-processed foods -G4 Lower** | **Sweden** | 52,750 | **636.3** | 301.6 | 5.1 | 413.8 | 585.0 | 802.4 | 2,703.5 |
| **Energy: Ultra-processed foods -G4 Middle** | **Sweden** | 52,750 | **702.9** | 318.6 | 5.1 | 469.1 | 653.4 | 880.8 | 2,872.4 |
| **Energy: Ultra-processed foods -G4 Upper** | **Sweden** | 52,750 | **1,057.8** | 418.2 | 144.0 | 756.3 | 995.4 | 1,290.4 | 3,777.4 |
| **Energy: Ultra-processed foods -G4 Lower** | **Denmark** | 55,860 | **577.2** | 293.4 | 0 | 370.2 | 524.7 | 724.7 | 3,022.9 |
| **Energy: Ultra-processed foods -G4 Middle** | **Denmark** | 55,860 | **984.1** | 380.2 | 26.8 | 710.6 | 931.2 | 1,195.6 | 3,891.7 |
| **Energy: Ultra-processed foods -G4 Upper** | **Denmark** | 55,860 | **1,104.9** | 409.4 | 32.7 | 810.2 | 1,048.7 | 1,336.3 | 4,013.5 |
|  |  |  |  |  |  |  |  |  |  |
| **Label** | **Country** | **N** | **Mean** | **Std** | **Min** | **P25** | **P50** | **P75** | **Max** |
| **Energy: Ultra-processed foods -G4 Lower** | **Norway** | 36,448 | **424.3** | 167.6 | 4.2 | 303.5 | 404.4 | 521.5 | 1,419.0 |
| **Energy: Ultra-processed foods -G4 Middle** | **Norway** | 36,448 | **775.0** | 254.7 | 24.2 | 591.7 | 759.0 | 941.0 | 2,148.8 |
| **Energy: Ultra-processed foods -G4 Upper** | **Norway** | 36,448 | **877.7** | 276.8 | 68.1 | 679.9 | 860.4 | 1,058.0 | 2,240.9 |
| **Energy: Ultra-processed foods -G4 excluding alcohol -Lower** | **France** | 73,035 | **380.9** | 221.1 | 1.4 | 224.5 | 338.3 | 488.5 | 2,286.6 |
| **Energy: Ultra-processed foods -G4 excluding alcohol -Middle** | **France** | 73,035 | **405.5** | 230.6 | 1.4 | 241.9 | 362.7 | 520.0 | 2,315.8 |
| **Energy: Ultra-processed foods -G4 excluding alcohol -Upper** | **France** | 73,035 | **757.8** | 326.2 | 2.9 | 526.1 | 714.6 | 941.1 | 2,943.6 |
| **Energy: Ultra-processed foods -G4 excluding alcohol -Lower** | **Italy** | 45,908 | **346.5** | 205.3 | 0 | 202.5 | 307.7 | 445.3 | 2,241.7 |
| **Energy: Ultra-processed foods -G4 excluding alcohol -Middle** | **Italy** | 45,908 | **345.8** | 205.0 | 0 | 202.2 | 306.9 | 444.2 | 2,241.7 |
| **Energy: Ultra-processed foods -G4 excluding alcohol -Upper** | **Italy** | 45,908 | **841.2** | 350.7 | 40.3 | 591.0 | 793.3 | 1,037.7 | 3,895.2 |
| **Energy: Ultra-processed foods -G4 excluding alcohol -Lower** | **Spain** | 40,621 | **332.6** | 234.1 | 0 | 163.9 | 285.1 | 448.0 | 2,732.8 |
| **Energy: Ultra-processed foods -G4 excluding alcohol -Middle** | **Spain** | 40,621 | **335.9** | 234.7 | 0 | 167.0 | 288.7 | 452.0 | 2,747.6 |
| **Energy: Ultra-processed foods -G4 excluding alcohol -Upper** | **Spain** | 40,621 | **773.6** | 362.1 | 0 | 514.7 | 721.7 | 977.0 | 3,489.7 |
| **Energy: Ultra-processed foods -G4 excluding alcohol -Lower** | **United Kingdom** | 81,097 | **679.5** | 338.0 | 0 | 439.6 | 614.7 | 849.2 | 3,824.0 |
| **Energy: Ultra-processed foods -G4 excluding alcohol -Middle** | **United Kingdom** | 81,097 | **939.8** | 405.8 | 0 | 647.9 | 873.5 | 1,160.0 | 4,481.0 |
| **Energy: Ultra-processed foods -G4 excluding alcohol -Upper** | **United Kingdom** | 81,097 | **1,036.4** | 435.0 | 0 | 721.9 | 968.1 | 1,278.2 | 4,651.4 |
| **Energy: Ultra-processed foods -G4 excluding alcohol -Lower** | **The Netherlands** | 39,036 | **636.9** | 261.9 | 37.9 | 452.6 | 600.7 | 775.8 | 3,242.3 |
| **Energy: Ultra-processed foods -G4 excluding alcohol -Middle** | **The Netherlands** | 39,036 | **698.8** | 289.0 | 38.9 | 495.4 | 656.0 | 849.4 | 3,420.4 |
| **Energy: Ultra-processed foods -G4 excluding alcohol -Upper** | **The Netherlands** | 39,036 | **1,068.5** | 396.2 | 50.6 | 791.3 | 1,009.2 | 1,277.3 | 4,102.0 |
| **Energy: Ultra-processed foods -G4 excluding alcohol -Lower** | **Germany** | 52,013 | **698.9** | 356.5 | 0.8 | 448.9 | 632.4 | 874.2 | 4,178.6 |
| **Energy: Ultra-processed foods -G4 excluding alcohol -Middle** | **Germany** | 52,013 | **714.7** | 359.7 | 0.8 | 462.1 | 648.6 | 893.1 | 4,206.8 |
| **Energy: Ultra-processed foods -G4 excluding alcohol -Upper** | **Germany** | 52,013 | **1,178.1** | 464.2 | 2.1 | 850.6 | 1,106.7 | 1,427.6 | 4,553.1 |
| **Energy: Ultra-processed foods -G4 excluding alcohol -Lower** | **Sweden** | 52,750 | **614.9** | 290.5 | 5.1 | 402.4 | 565.7 | 773.5 | 2,703.5 |
| **Energy: Ultra-processed foods -G4 excluding alcohol -Middle** | **Sweden** | 52,750 | **681.5** | 307.5 | 5.1 | 457.2 | 633.9 | 852.8 | 2,872.4 |
| **Energy: Ultra-processed foods -G4 excluding alcohol -Upper** | **Sweden** | 52,750 | **1,036.4** | 410.3 | 144.0 | 740.8 | 974.2 | 1,263.2 | 3,777.4 |
| **Energy: Ultra-processed foods -G4 excluding alcohol -Lower** | **Denmark** | 55,860 | **553.3** | 290.1 | 0 | 348.3 | 499.7 | 697.4 | 3,022.9 |
| **Energy: Ultra-processed foods -G4 excluding alcohol -Middle** | **Denmark** | 55,860 | **960.3** | 378.0 | 24.5 | 689.1 | 906.4 | 1,170.0 | 3,891.7 |
| **Energy: Ultra-processed foods -G4 excluding alcohol -Upper** | **Denmark** | 55,860 | **1,081.0** | 407.3 | 30.4 | 787.6 | 1,024.2 | 1,309.5 | 4,013.5 |
|  |  |  |  |  |  |  |  |  |  |
| **Label** | **Country** | **N** | **Mean** | **Std** | **Min** | **P25** | **P50** | **P75** | **Max** |
| **Energy: Ultra-processed foods -G4 excluding alcohol -Lower** | **Norway** | 36,448 | **422.3** | 167.6 | 4.2 | 301.6 | 402.3 | 519.7 | 1,419.0 |
| **Energy: Ultra-processed foods -G4 excluding alcohol -Middle** | **Norway** | 36,448 | **773.0** | 254.9 | 24.2 | 589.3 | 757.0 | 939.4 | 2,148.8 |
| **Energy: Ultra-processed foods -G4 excluding alcohol -Upper** | **Norway** | 36,448 | **875.7** | 277.0 | 68.1 | 677.7 | 858.1 | 1,056.2 | 2,236.6 |
| **% gram/day excluding alcohol** |  |  |  |  |  |  |  |  |  |
| **Unprocessed or minimally processed foods -G1 Lower** | **France** | 73,035 | **83.8** | 6.1 | 31.9 | 80.3 | 84.4 | 88.0 | 98.9 |
| **Unprocessed or minimally processed foods -G1 Middle** | **France** | 73,035 | **83.0** | 6.4 | 31.0 | 79.4 | 83.7 | 87.5 | 98.9 |
| **Unprocessed or minimally processed foods -G1 Upper** | **France** | 73,035 | **77.8** | 7.6 | 28.4 | 73.2 | 78.4 | 83.1 | 98.8 |
| **Unprocessed or minimally processed foods -G1 Lower** | **Italy** | 45,908 | **71.4** | 8.5 | 11.8 | 66.5 | 72.1 | 77.3 | 95.7 |
| **Unprocessed or minimally processed foods -G1 Middle** | **Italy** | 45,908 | **66.0** | 9.1 | 10.2 | 60.5 | 66.6 | 72.2 | 92.8 |
| **Unprocessed or minimally processed foods -G1 Upper** | **Italy** | 45,908 | **61.9** | 9.2 | 6.9 | 56.3 | 62.4 | 68.2 | 91.4 |
| **Unprocessed or minimally processed foods -G1 Lower** | **Spain** | 40,621 | **78.7** | 8.8 | 18.6 | 73.8 | 79.9 | 84.9 | 100.0 |
| **Unprocessed or minimally processed foods -G1 Middle** | **Spain** | 40,621 | **76.3** | 9.0 | 17.3 | 71.1 | 77.3 | 82.7 | 99.7 |
| **Unprocessed or minimally processed foods -G1 Upper** | **Spain** | 40,621 | **69.5** | 10.0 | 12.8 | 63.5 | 70.4 | 76.5 | 99.7 |
| **Unprocessed or minimally processed foods -G1 Lower** | **United Kingdom** | 81,097 | **79.0** | 9.4 | 9.1 | 74.9 | 80.9 | 85.3 | 100.0 |
| **Unprocessed or minimally processed foods -G1 Middle** | **United Kingdom** | 81,097 | **76.7** | 9.7 | 7.8 | 72.2 | 78.5 | 83.3 | 100.0 |
| **Unprocessed or minimally processed foods -G1 Upper** | **United Kingdom** | 81,097 | **73.4** | 10.0 | 6.5 | 68.7 | 75.3 | 80.4 | 100.0 |
| **Unprocessed or minimally processed foods -G1 Lower** | **The Netherlands** | 39,036 | **77.7** | 8.2 | 22.5 | 73.1 | 78.9 | 83.5 | 98.6 |
| **Unprocessed or minimally processed foods -G1 Middle** | **The Netherlands** | 39,036 | **76.8** | 8.7 | 21.4 | 71.9 | 78.0 | 82.9 | 98.6 |
| **Unprocessed or minimally processed foods -G1 Upper** | **The Netherlands** | 39,036 | **74.1** | 9.1 | 18.4 | 69.0 | 75.3 | 80.6 | 98.5 |
| **Unprocessed or minimally processed foods -G1 Lower** | **Germany** | 52,013 | **71.5** | 11.0 | 9.0 | 65.0 | 72.8 | 79.5 | 97.7 |
| **Unprocessed or minimally processed foods -G1 Middle** | **Germany** | 52,013 | **70.4** | 11.2 | 7.8 | 63.7 | 71.6 | 78.5 | 97.4 |
| **Unprocessed or minimally processed foods -G1 Upper** | **Germany** | 52,013 | **68.5** | 11.4 | 7.1 | 61.6 | 69.7 | 76.8 | 96.9 |
| **Unprocessed or minimally processed foods -G1 Lower** | **Sweden** | 52,750 | **79.5** | 8.2 | 16.0 | 75.1 | 80.7 | 85.2 | 98.2 |
| **Unprocessed or minimally processed foods -G1 Middle** | **Sweden** | 52,750 | **78.0** | 8.4 | 16.0 | 73.4 | 79.2 | 83.9 | 97.7 |
| **Unprocessed or minimally processed foods -G1 Upper** | **Sweden** | 52,750 | **76.4** | 8.8 | 14.2 | 71.5 | 77.5 | 82.6 | 97.6 |
| **Unprocessed or minimally processed foods -G1 Lower** | **Denmark** | 55,860 | **83.4** | 8.1 | 13.0 | 80.0 | 85.2 | 88.9 | 98.8 |
| **Unprocessed or minimally processed foods -G1 Middle** | **Denmark** | 55,860 | **81.2** | 8.4 | 12.4 | 77.5 | 83.0 | 87.1 | 98.6 |
| **Unprocessed or minimally processed foods -G1 Upper** | **Denmark** | 55,860 | **79.5** | 8.7 | 11.2 | 75.3 | 81.1 | 85.5 | 98.2 |
|  |  |  |  |  |  |  |  |  |  |
| **Label** | **Country** | **N** | **Mean** | **Std** | **Min** | **P25** | **P50** | **P75** | **Max** |
| **Unprocessed or minimally processed foods -G1 Lower** | **Norway** | 36,448 | **73.3** | 9.3 | 1.8 | 68.6 | 74.7 | 79.7 | 97.0 |
| **Unprocessed or minimally processed foods -G1 Middle** | **Norway** | 36,448 | **70.3** | 9.7 | 1.8 | 65.2 | 71.6 | 76.9 | 96.6 |
| **Unprocessed or minimally processed foods -G1 Upper** | **Norway** | 36,448 | **67.2** | 10.1 | 1.8 | 61.6 | 68.4 | 74.1 | 96.6 |
| **Processed culinary ingredients -G2 Lower** | **France** | 73,035 | **1.6** | 0.7 | 0 | 1.1 | 1.5 | 2.0 | 12.7 |
| **Processed culinary ingredients -G2 Middle** | **France** | 73,035 | **1.5** | 0.7 | 0 | 1.0 | 1.4 | 1.9 | 12.6 |
| **Processed culinary ingredients -G2 Upper** | **France** | 73,035 | **1.4** | 0.7 | 0 | 0.9 | 1.3 | 1.8 | 12.5 |
| **Processed culinary ingredients -G2 Lower** | **Italy** | 45,908 | **3.2** | 1.1 | 0 | 2.4 | 3.1 | 3.8 | 12.2 |
| **Processed culinary ingredients -G2 Middle** | **Italy** | 45,908 | **3.0** | 1.0 | 0 | 2.3 | 2.9 | 3.6 | 11.7 |
| **Processed culinary ingredients -G2 Upper** | **Italy** | 45,908 | **2.8** | 1.0 | 0 | 2.1 | 2.7 | 3.4 | 11.6 |
| **Processed culinary ingredients -G2 Lower** | **Spain** | 40,621 | **2.5** | 1.1 | 0 | 1.7 | 2.3 | 3.1 | 15.7 |
| **Processed culinary ingredients -G2 Middle** | **Spain** | 40,621 | **2.4** | 1.1 | 0 | 1.7 | 2.3 | 3.0 | 15.7 |
| **Processed culinary ingredients -G2 Upper** | **Spain** | 40,621 | **2.4** | 1.1 | 0 | 1.6 | 2.3 | 3.0 | 15.7 |
| **Processed culinary ingredients -G2 Lower** | **United Kingdom** | 81,097 | **0.9** | 0.7 | 0 | 0.4 | 0.7 | 1.3 | 8.6 |
| **Processed culinary ingredients -G2 Middle** | **United Kingdom** | 81,097 | **0.6** | 0.6 | 0 | 0.2 | 0.4 | 0.8 | 8.2 |
| **Processed culinary ingredients -G2 Upper** | **United Kingdom** | 81,097 | **0.5** | 0.6 | 0 | 0.1 | 0.3 | 0.8 | 8.1 |
| **Processed culinary ingredients -G2 Lower** | **The Netherlands** | 39,036 | **1.0** | 0.8 | 0.001 | 0.4 | 0.6 | 1.3 | 8.3 |
| **Processed culinary ingredients -G2 Middle** | **The Netherlands** | 39,036 | **0.9** | 0.8 | 0.001 | 0.3 | 0.5 | 1.2 | 8.3 |
| **Processed culinary ingredients -G2 Upper** | **The Netherlands** | 39,036 | **0.8** | 0.8 | 0.001 | 0.3 | 0.5 | 1.2 | 8.3 |
| **Processed culinary ingredients -G2 Lower** | **Germany** | 52,013 | **1.3** | 0.9 | 0.004 | 0.6 | 1.0 | 1.7 | 12.5 |
| **Processed culinary ingredients -G2 Middle** | **Germany** | 52,013 | **1.1** | 0.9 | 0.004 | 0.5 | 0.9 | 1.5 | 12.4 |
| **Processed culinary ingredients -G2 Upper** | **Germany** | 52,013 | **1.0** | 0.9 | 0 | 0.4 | 0.8 | 1.4 | 12.3 |
| **Processed culinary ingredients -G2 Lower** | **Sweden** | 52,750 | **1.2** | 1.1 | 0 | 0.5 | 0.9 | 1.6 | 15.9 |
| **Processed culinary ingredients -G2 Middle** | **Sweden** | 52,750 | **1.2** | 1.1 | 0 | 0.5 | 0.9 | 1.5 | 14.5 |
| **Processed culinary ingredients -G2 Upper** | **Sweden** | 52,750 | **1.0** | 1.1 | 0 | 0.3 | 0.7 | 1.3 | 14.2 |
| **Processed culinary ingredients -G2 Lower** | **Denmark** | 55,860 | **0.7** | 0.5 | 0 | 0.3 | 0.5 | 0.8 | 6.6 |
| **Processed culinary ingredients -G2 Middle** | **Denmark** | 55,860 | **0.5** | 0.4 | 0 | 0.2 | 0.4 | 0.7 | 6.3 |
| **Processed culinary ingredients -G2 Upper** | **Denmark** | 55,860 | **0.4** | 0.4 | 0 | 0.1 | 0.2 | 0.5 | 6.2 |
|  |  |  |  |  |  |  |  |  |  |
| **Label** | **Country** | **N** | **Mean** | **Std** | **Min** | **P25** | **P50** | **P75** | **Max** |
| **Processed culinary ingredients -G2 Lower** | **Norway** | 36,448 | **0.9** | 0.6 | 0 | 0.5 | 0.7 | 1.1 | 10.6 |
| **Processed culinary ingredients -G2 Middle** | **Norway** | 36,448 | **0.8** | 0.6 | 0 | 0.4 | 0.6 | 1.0 | 10.4 |
| **Processed culinary ingredients -G2 Upper** | **Norway** | 36,448 | **0.6** | 0.5 | 0 | 0.2 | 0.4 | 0.7 | 10.1 |
| **Processed foods -G3 Lower** | **France** | 73,035 | **8.3** | 3.9 | 0.03 | 5.5 | 7.8 | 10.5 | 44.4 |
| **Processed foods -G3 Middle** | **France** | 73,035 | **8.5** | 4.0 | 0.02 | 5.7 | 8.0 | 10.8 | 44.8 |
| **Processed foods -G3 Upper** | **France** | 73,035 | **5.8** | 2.7 | 0 | 3.9 | 5.3 | 7.1 | 37.2 |
| **Processed foods -G3 Lower** | **Italy** | 45,908 | **14.4** | 5.9 | 0.4 | 10.3 | 13.8 | 17.9 | 58.9 |
| **Processed foods -G3 Middle** | **Italy** | 45,908 | **20.1** | 7.2 | 0.9 | 15.0 | 19.4 | 24.3 | 65.2 |
| **Processed foods -G3 Upper** | **Italy** | 45,908 | **7.4** | 3.8 | 0 | 4.8 | 6.7 | 9.2 | 52.7 |
| **Processed foods -G3 Lower** | **Spain** | 40,621 | **10.5** | 5.6 | 0 | 6.5 | 9.8 | 13.8 | 44.1 |
| **Processed foods -G3 Middle** | **Spain** | 40,621 | **12.9** | 6.1 | 0 | 8.5 | 12.2 | 16.5 | 54.8 |
| **Processed foods -G3 Upper** | **Spain** | 40,621 | **7.6** | 3.5 | 0 | 5.1 | 7.1 | 9.4 | 50.9 |
| **Processed foods -G3 Lower** | **United Kingdom** | 81,097 | **5.5** | 2.8 | 0 | 3.5 | 5.0 | 7.0 | 33.6 |
| **Processed foods -G3 Middle** | **United Kingdom** | 81,097 | **3.6** | 2.0 | 0 | 2.3 | 3.2 | 4.5 | 33.5 |
| **Processed foods -G3 Upper** | **United Kingdom** | 81,097 | **4.8** | 2.3 | 0 | 3.3 | 4.4 | 5.9 | 37.1 |
| **Processed foods -G3 Lower** | **The Netherlands** | 39,036 | **7.9** | 3.3 | 0.02 | 5.6 | 7.5 | 9.7 | 34.1 |
| **Processed foods -G3 Middle** | **The Netherlands** | 39,036 | **7.4** | 3.1 | 0.02 | 5.2 | 6.9 | 9.1 | 32.8 |
| **Processed foods -G3 Upper** | **The Netherlands** | 39,036 | **3.2** | 1.6 | 0.06 | 2.2 | 2.9 | 4.0 | 22.6 |
| **Processed foods -G3 Lower** | **Germany** | 52,013 | **9.6** | 4.2 | 0 | 6.6 | 9.1 | 12.1 | 41.4 |
| **Processed foods -G3 Middle** | **Germany** | 52,013 | **10.4** | 4.3 | 0 | 7.3 | 9.9 | 13.0 | 41.9 |
| **Processed foods -G3 Upper** | **Germany** | 52,013 | **4.6** | 2.2 | 0 | 3.0 | 4.2 | 5.7 | 32.9 |
| **Processed foods -G3 Lower** | **Sweden** | 52,750 | **7.5** | 3.3 | 0 | 5.2 | 6.9 | 9.2 | 44.0 |
| **Processed foods -G3 Middle** | **Sweden** | 52,750 | **7.3** | 3.3 | 0 | 5.0 | 6.8 | 9.0 | 44.0 |
| **Processed foods -G3 Upper** | **Sweden** | 52,750 | **2.6** | 1.8 | 0 | 1.4 | 2.2 | 3.5 | 36.5 |
| **Processed foods -G3 Lower** | **Denmark** | 55,860 | **7.3** | 2.9 | 0.08 | 5.2 | 6.9 | 8.9 | 39.5 |
| **Processed foods -G3 Middle** | **Denmark** | 55,860 | **3.9** | 2.0 | 0 | 2.6 | 3.5 | 4.7 | 29.5 |
| **Processed foods -G3 Upper** | **Denmark** | 55,860 | **3.1** | 1.7 | 0 | 2.0 | 2.8 | 3.9 | 27.6 |
|  |  |  |  |  |  |  |  |  |  |
| **Label** | **Country** | **N** | **Mean** | **Std** | **Min** | **P25** | **P50** | **P75** | **Max** |
| **Processed foods -G3 Lower** | **Norway** | 36,448 | **11.7** | 4.9 | 0.2 | 8.3 | 11.1 | 14.4 | 51.9 |
| **Processed foods -G3 Middle** | **Norway** | 36,448 | **5.5** | 2.5 | 0 | 3.7 | 5.2 | 6.9 | 30.4 |
| **Processed foods -G3 Upper** | **Norway** | 36,448 | **3.7** | 1.9 | 0 | 2.4 | 3.4 | 4.7 | 23.8 |
| **Ultra-processed foods -G4 Lower** | **France** | 73,035 | **6.3** | 4.3 | 0.01 | 3.4 | 5.3 | 8.0 | 64.0 |
| **Ultra-processed foods -G4 Middle** | **France** | 73,035 | **7.0** | 4.5 | 0.01 | 3.9 | 6.0 | 8.9 | 64.1 |
| **Ultra-processed foods -G4 Upper** | **France** | 73,035 | **15.1** | 6.7 | 0 | 10.3 | 14.4 | 19.0 | 66.7 |
| **Ultra-processed foods -G4 Lower** | **Italy** | 45,908 | **11.0** | 6.9 | 0 | 6.3 | 9.4 | 14.0 | 77.6 |
| **Ultra-processed foods -G4 Middle** | **Italy** | 45,908 | **11.0** | 6.9 | 0 | 6.3 | 9.4 | 14.0 | 77.6 |
| **Ultra-processed foods -G4 Upper** | **Italy** | 45,908 | **27.9** | 8.9 | 3.0 | 21.7 | 27.1 | 33.2 | 87.5 |
| **Ultra-processed foods -G4 Lower** | **Spain** | 40,621 | **8.3** | 6.8 | 0 | 3.7 | 6.5 | 10.9 | 68.3 |
| **Ultra-processed foods -G4 Middle** | **Spain** | 40,621 | **8.4** | 6.8 | 0 | 3.8 | 6.6 | 11.1 | 68.3 |
| **Ultra-processed foods -G4 Upper** | **Spain** | 40,621 | **20.5** | 9.0 | 0 | 14.2 | 19.4 | 25.5 | 79.6 |
| **Ultra-processed foods -G4 Lower** | **United Kingdom** | 81,097 | **14.6** | 8.9 | 0 | 8.6 | 12.3 | 18.0 | 89.8 |
| **Ultra-processed foods -G4 Middle** | **United Kingdom** | 81,097 | **19.1** | 9.4 | 0 | 12.7 | 17.0 | 23.1 | 90.7 |
| **Ultra-processed foods -G4 Upper** | **United Kingdom** | 81,097 | **21.2** | 9.6 | 0 | 14.6 | 19.2 | 25.4 | 91.3 |
| **Ultra-processed foods -G4 Lower** | **The Netherlands** | 39,036 | **13.5** | 6.8 | 1.1 | 8.7 | 12.1 | 16.6 | 68.1 |
| **Ultra-processed foods -G4 Middle** | **The Netherlands** | 39,036 | **15.0** | 7.3 | 1.1 | 9.9 | 13.6 | 18.5 | 69.8 |
| **Ultra-processed foods -G4 Upper** | **The Netherlands** | 39,036 | **21.8** | 8.6 | 1.2 | 15.7 | 20.5 | 26.4 | 75.6 |
| **Ultra-processed foods -G4 Lower** | **Germany** | 52,013 | **17.6** | 9.6 | 0 | 10.7 | 15.8 | 22.4 | 85.2 |
| **Ultra-processed foods -G4 Middle** | **Germany** | 52,013 | **18.0** | 9.7 | 0 | 11.1 | 16.2 | 22.9 | 85.8 |
| **Ultra-processed foods -G4 Upper** | **Germany** | 52,013 | **25.9** | 10.7 | 0 | 18.2 | 24.5 | 31.9 | 88.5 |
| **Ultra-processed foods -G4 Lower** | **Sweden** | 52,750 | **11.8** | 6.8 | 0 | 7.2 | 10.3 | 14.8 | 80.8 |
| **Ultra-processed foods -G4 Middle** | **Sweden** | 52,750 | **13.5** | 7.1 | 0 | 8.6 | 12.1 | 16.8 | 80.8 |
| **Ultra-processed foods -G4 Upper** | **Sweden** | 52,750 | **19.9** | 8.5 | 1.1 | 14.0 | 18.8 | 24.5 | 83.5 |
| **Ultra-processed foods -G4 Lower** | **Denmark** | 55,860 | **8.7** | 7.5 | 0 | 3.8 | 6.3 | 10.8 | 83.6 |
| **Ultra-processed foods -G4 Middle** | **Denmark** | 55,860 | **14.4** | 8.0 | 0.6 | 9.0 | 12.4 | 17.5 | 86.3 |
| **Ultra-processed foods -G4 Upper** | **Denmark** | 55,860 | **17.0** | 8.4 | 1.0 | 11.2 | 15.1 | 20.7 | 87.9 |
|  |  |  |  |  |  |  |  |  |  |
| **Label** | **Country** | **N** | **Mean** | **Std** | **Min** | **P25** | **P50** | **P75** | **Max** |
| **Ultra-processed foods -G4 Lower** | **Norway** | 36,448 | **14.2** | 7.9 | 0.2 | 9.0 | 12.4 | 17.0 | 79.5 |
| **Ultra-processed foods -G4 Middle** | **Norway** | 36,448 | **23.4** | 9.0 | 1.0 | 17.4 | 22.0 | 27.7 | 98.1 |
| **Ultra-processed foods -G4 Upper** | **Norway** | 36,448 | **28.5** | 9.6 | 1.8 | 22.0 | 27.2 | 33.5 | 98.1 |
| **%kcal/day excluding alcohol** |  |  |  |  |  |  |  |  |  |
| **Energy: Unprocessed or minimally processed foods -G1 Lower** | **France** | 73,035 | **42.8** | 9.8 | 4.8 | 36.1 | 42.2 | 49.0 | 89.7 |
| **Energy: Unprocessed or minimally processed foods -G1 Middle** | **France** | 73,035 | **41.8** | 9.9 | 4.8 | 34.9 | 41.2 | 48.0 | 88.9 |
| **Energy: Unprocessed or minimally processed foods -G1 Upper** | **France** | 73,035 | **36.9** | 9.7 | 1.4 | 30.1 | 36.1 | 42.9 | 85.4 |
| **Energy: Unprocessed or minimally processed foods -G1 Lower** | **Italy** | 45,908 | **39.2** | 8.1 | 8.0 | 33.8 | 38.9 | 44.2 | 81.5 |
| **Energy: Unprocessed or minimally processed foods -G1 Middle** | **Italy** | 45,908 | **36.5** | 7.7 | 6.9 | 31.3 | 36.4 | 41.5 | 76.5 |
| **Energy: Unprocessed or minimally processed foods -G1 Upper** | **Italy** | 45,908 | **33.3** | 7.6 | 5.4 | 28.2 | 33.1 | 38.2 | 75.4 |
| **Energy: Unprocessed or minimally processed foods -G1 Lower** | **Spain** | 40,621 | **46.6** | 9.7 | 6.7 | 40.0 | 46.1 | 52.7 | 99.8 |
| **Energy: Unprocessed or minimally processed foods -G1 Middle** | **Spain** | 40,621 | **44.7** | 9.7 | 6.6 | 38.1 | 44.1 | 50.7 | 99.5 |
| **Energy: Unprocessed or minimally processed foods -G1 Upper** | **Spain** | 40,621 | **38.0** | 9.3 | 0.7 | 31.6 | 37.3 | 43.6 | 99.5 |
| **Energy: Unprocessed or minimally processed foods -G1 Lower** | **United Kingdom** | 81,097 | **43.7** | 10.9 | 6.8 | 36.2 | 43.2 | 50.7 | 99.8 |
| **Energy: Unprocessed or minimally processed foods -G1 Middle** | **United Kingdom** | 81,097 | **39.5** | 11.1 | 5.4 | 31.6 | 38.8 | 46.6 | 99.8 |
| **Energy: Unprocessed or minimally processed foods -G1 Upper** | **United Kingdom** | 81,097 | **35.9** | 10.5 | 3.6 | 28.5 | 35.2 | 42.6 | 99.8 |
| **Energy: Unprocessed or minimally processed foods -G1 Lower** | **The Netherlands** | 39,036 | **36.8** | 8.6 | 5.0 | 30.8 | 36.4 | 42.3 | 85.6 |
| **Energy: Unprocessed or minimally processed foods -G1 Middle** | **The Netherlands** | 39,036 | **35.8** | 8.8 | 3.4 | 29.7 | 35.3 | 41.5 | 85.6 |
| **Energy: Unprocessed or minimally processed foods -G1 Upper** | **The Netherlands** | 39,036 | **33.5** | 8.6 | 3.2 | 27.5 | 33.1 | 39.0 | 82.1 |
| **Energy: Unprocessed or minimally processed foods -G1 Lower** | **Germany** | 52,013 | **28.2** | 8.2 | 4.8 | 22.6 | 27.3 | 32.8 | 82.9 |
| **Energy: Unprocessed or minimally processed foods -G1 Middle** | **Germany** | 52,013 | **26.5** | 8.2 | 3.6 | 20.8 | 25.4 | 31.0 | 82.1 |
| **Energy: Unprocessed or minimally processed foods -G1 Upper** | **Germany** | 52,013 | **23.9** | 7.9 | 2.6 | 18.4 | 22.8 | 28.2 | 81.9 |
| **Energy: Unprocessed or minimally processed foods -G1 Lower** | **Sweden** | 52,750 | **40.7** | 10.5 | 1.2 | 33.3 | 40.4 | 47.8 | 92.3 |
| **Energy: Unprocessed or minimally processed foods -G1 Middle** | **Sweden** | 52,750 | **38.3** | 10.4 | 1.2 | 30.9 | 37.8 | 45.1 | 90.8 |
| **Energy: Unprocessed or minimally processed foods -G1 Upper** | **Sweden** | 52,750 | **35.9** | 10.1 | 1.2 | 28.7 | 35.2 | 42.3 | 89.2 |
| **Energy: Unprocessed or minimally processed foods -G1 Lower** | **Denmark** | 55,860 | **40.9** | 9.4 | 5.2 | 34.5 | 40.5 | 47.0 | 97.3 |
| **Energy: Unprocessed or minimally processed foods -G1 Middle** | **Denmark** | 55,860 | **38.2** | 9.2 | 4.2 | 31.9 | 37.7 | 44.1 | 84.6 |
| **Energy: Unprocessed or minimally processed foods -G1 Upper** | **Denmark** | 55,860 | **34.7** | 9.0 | 3.8 | 28.5 | 34.2 | 40.4 | 81.9 |
|  |  |  |  |  |  |  |  |  |  |
| **Label** | **Country** | **N** | **Mean** | **Std** | **Min** | **P25** | **P50** | **P75** | **Max** |
| **Energy: Unprocessed or minimally processed foods -G1 Lower** | **Norway** | 36,448 | **41.5** | 9.1 | 0.8 | 35.4 | 41.2 | 47.3 | 89.3 |
| **Energy: Unprocessed or minimally processed foods -G1 Middle** | **Norway** | 36,448 | **38.4** | 9.0 | 0.8 | 32.3 | 38.0 | 44.0 | 89.3 |
| **Energy: Unprocessed or minimally processed foods -G1 Upper** | **Norway** | 36,448 | **34.4** | 9.0 | 0.8 | 28.2 | 33.7 | 40.0 | 89.3 |
| **Energy: Processed culinary ingredients -G2 Lower** | **France** | 73,035 | **11.4** | 4.5 | 0 | 8.3 | 11.0 | 14.0 | 48.9 |
| **Energy: Processed culinary ingredients -G2 Middle** | **France** | 73,035 | **11.0** | 4.5 | 0 | 7.9 | 10.6 | 13.6 | 48.9 |
| **Energy: Processed culinary ingredients -G2 Upper** | **France** | 73,035 | **10.5** | 4.6 | 0 | 7.3 | 10.0 | 13.1 | 45.5 |
| **Energy: Processed culinary ingredients -G2 Lower** | **Italy** | 45,908 | **16.7** | 4.7 | 0 | 13.5 | 16.3 | 19.5 | 48.1 |
| **Energy: Processed culinary ingredients -G2 Middle** | **Italy** | 45,908 | **16.3** | 4.8 | 0 | 13.1 | 15.9 | 19.1 | 48.1 |
| **Energy: Processed culinary ingredients -G2 Upper** | **Italy** | 45,908 | **15.9** | 4.8 | 0 | 12.6 | 15.4 | 18.6 | 48.1 |
| **Energy: Processed culinary ingredients -G2 Lower** | **Spain** | 40,621 | **14.7** | 5.3 | 0 | 10.9 | 14.2 | 17.9 | 57.5 |
| **Energy: Processed culinary ingredients -G2 Middle** | **Spain** | 40,621 | **14.6** | 5.3 | 0 | 10.8 | 14.1 | 17.8 | 57.5 |
| **Energy: Processed culinary ingredients -G2 Upper** | **Spain** | 40,621 | **14.6** | 5.3 | 0 | 10.8 | 14.1 | 17.8 | 57.5 |
| **Energy: Processed culinary ingredients -G2 Lower** | **United Kingdom** | 81,097 | **5.4** | 4.4 | 0 | 2.4 | 4.0 | 7.1 | 40.5 |
| **Energy: Processed culinary ingredients -G2 Middle** | **United Kingdom** | 81,097 | **3.3** | 4.0 | 0 | 0.6 | 1.5 | 4.6 | 38.9 |
| **Energy: Processed culinary ingredients -G2 Upper** | **United Kingdom** | 81,097 | **3.2** | 4.0 | 0 | 0.5 | 1.4 | 4.5 | 38.9 |
| **Energy: Processed culinary ingredients -G2 Lower** | **The Netherlands** | 39,036 | **6.9** | 4.7 | 0.01 | 3.5 | 5.7 | 9.2 | 52.0 |
| **Energy: Processed culinary ingredients -G2 Middle** | **The Netherlands** | 39,036 | **5.8** | 4.4 | 0.01 | 2.4 | 4.6 | 7.9 | 51.9 |
| **Energy: Processed culinary ingredients -G2 Upper** | **The Netherlands** | 39,036 | **5.7** | 4.4 | 0.01 | 2.4 | 4.6 | 7.8 | 51.9 |
| **Energy: Processed culinary ingredients -G2 Lower** | **Germany** | 52,013 | **8.6** | 5.3 | 0.01 | 4.6 | 7.5 | 11.4 | 54.7 |
| **Energy: Processed culinary ingredients -G2 Middle** | **Germany** | 52,013 | **8.0** | 5.3 | 0.01 | 4.0 | 6.9 | 10.8 | 54.6 |
| **Energy: Processed culinary ingredients -G2 Upper** | **Germany** | 52,013 | **7.5** | 5.2 | 0 | 3.6 | 6.4 | 10.3 | 54.6 |
| **Energy: Processed culinary ingredients -G2 Lower** | **Sweden** | 52,750 | **5.7** | 5.1 | 0 | 2.2 | 4.2 | 7.3 | 51.9 |
| **Energy: Processed culinary ingredients -G2 Middle** | **Sweden** | 52,750 | **5.4** | 5.1 | 0 | 2.0 | 4.0 | 7.0 | 51.5 |
| **Energy: Processed culinary ingredients -G2 Upper** | **Sweden** | 52,750 | **5.0** | 4.9 | 0 | 1.7 | 3.5 | 6.5 | 51.5 |
| **Energy: Processed culinary ingredients -G2 Lower** | **Denmark** | 55,860 | **4.1** | 3.5 | 0 | 1.5 | 3.1 | 5.5 | 27.8 |
| **Energy: Processed culinary ingredients -G2 Middle** | **Denmark** | 55,860 | **3.5** | 3.5 | 0 | 0.8 | 2.4 | 4.8 | 27.5 |
| **Energy: Processed culinary ingredients -G2 Upper** | **Denmark** | 55,860 | **2.9** | 3.4 | 0 | 0.4 | 1.7 | 4.1 | 27.3 |
|  |  |  |  |  |  |  |  |  |  |
| **Label** | **Country** | **N** | **Mean** | **Std** | **Min** | **P25** | **P50** | **P75** | **Max** |
| **Energy: Processed culinary ingredients -G2 Lower** | **Norway** | 36,448 | **4.5** | 3.1 | 0 | 2.5 | 3.6 | 5.6 | 36.3 |
| **Energy: Processed culinary ingredients -G2 Middle** | **Norway** | 36,448 | **4.1** | 3.1 | 0 | 2.1 | 3.2 | 5.1 | 36.0 |
| **Energy: Processed culinary ingredients -G2 Upper** | **Norway** | 36,448 | **3.2** | 3.0 | 0 | 1.3 | 2.1 | 4.0 | 35.6 |
| **Energy: Processed foods -G3 Lower** | **France** | 73,035 | **27.5** | 10.6 | 0.03 | 20.1 | 27.1 | 34.3 | 85.1 |
| **Energy: Processed foods -G3 Middle** | **France** | 73,035 | **27.7** | 10.6 | 0.01 | 20.3 | 27.3 | 34.5 | 85.1 |
| **Energy: Processed foods -G3 Upper** | **France** | 73,035 | **16.3** | 7.7 | 0 | 11.1 | 15.1 | 20.2 | 81.0 |
| **Energy: Processed foods -G3 Lower** | **Italy** | 45,908 | **28.3** | 9.1 | 0.7 | 22.0 | 28.0 | 34.2 | 72.6 |
| **Energy: Processed foods -G3 Middle** | **Italy** | 45,908 | **31.4** | 9.0 | 2.1 | 25.3 | 31.1 | 37.2 | 74.6 |
| **Energy: Processed foods -G3 Upper** | **Italy** | 45,908 | **12.6** | 6.0 | 0 | 8.4 | 11.7 | 15.6 | 59.2 |
| **Energy: Processed foods -G3 Lower** | **Spain** | 40,621 | **23.1** | 10.0 | 0 | 16.1 | 22.8 | 29.8 | 72.7 |
| **Energy: Processed foods -G3 Middle** | **Spain** | 40,621 | **24.9** | 10.2 | 0 | 17.8 | 24.7 | 31.7 | 73.1 |
| **Energy: Processed foods -G3 Upper** | **Spain** | 40,621 | **11.5** | 6.3 | 0 | 7.0 | 10.2 | 14.6 | 63.8 |
| **Energy: Processed foods -G3 Lower** | **United Kingdom** | 81,097 | **17.7** | 7.5 | 0 | 12.2 | 16.9 | 22.3 | 67.7 |
| **Energy: Processed foods -G3 Middle** | **United Kingdom** | 81,097 | **11.2** | 5.4 | 0 | 7.4 | 10.3 | 13.9 | 59.3 |
| **Energy: Processed foods -G3 Upper** | **United Kingdom** | 81,097 | **10.3** | 4.8 | 0 | 7.1 | 9.5 | 12.5 | 73.0 |
| **Energy: Processed foods -G3 Lower** | **The Netherlands** | 39,036 | **26.0** | 7.4 | 0.04 | 21.1 | 25.8 | 30.7 | 69.4 |
| **Energy: Processed foods -G3 Middle** | **The Netherlands** | 39,036 | **25.3** | 7.4 | 0.04 | 20.4 | 25.0 | 29.9 | 69.3 |
| **Energy: Processed foods -G3 Upper** | **The Netherlands** | 39,036 | **10.0** | 4.3 | 0.08 | 7.0 | 9.4 | 12.4 | 56.4 |
| **Energy: Processed foods -G3 Lower** | **Germany** | 52,013 | **29.4** | 9.5 | 0 | 22.8 | 29.0 | 35.5 | 81.9 |
| **Energy: Processed foods -G3 Middle** | **Germany** | 52,013 | **30.9** | 9.4 | 0 | 24.5 | 30.6 | 37.0 | 82.9 |
| **Energy: Processed foods -G3 Upper** | **Germany** | 52,013 | **10.8** | 4.9 | 0 | 7.5 | 10.1 | 13.3 | 67.6 |
| **Energy: Processed foods -G3 Lower** | **Sweden** | 52,750 | **23.2** | 7.9 | 0 | 17.6 | 22.4 | 28.0 | 72.8 |
| **Energy: Processed foods -G3 Middle** | **Sweden** | 52,750 | **22.4** | 7.8 | 0 | 17.0 | 21.7 | 27.2 | 72.7 |
| **Energy: Processed foods -G3 Upper** | **Sweden** | 52,750 | **7.6** | 4.7 | 0 | 4.2 | 6.7 | 10.2 | 59.3 |
| **Energy: Processed foods -G3 Lower** | **Denmark** | 55,860 | **28.5** | 8.6 | 0.4 | 22.5 | 28.1 | 34.0 | 76.3 |
| **Energy: Processed foods -G3 Middle** | **Denmark** | 55,860 | **11.9** | 4.7 | 0 | 8.5 | 11.4 | 14.7 | 45.4 |
| **Energy: Processed foods -G3 Upper** | **Denmark** | 55,860 | **10.0** | 4.7 | 0 | 6.5 | 9.3 | 12.7 | 46.2 |
|  |  |  |  |  |  |  |  |  |  |
| **Label** | **Country** | **N** | **Mean** | **Std** | **Min** | **P25** | **P50** | **P75** | **Max** |
| **Energy: Processed foods -G3 Lower** | **Norway** | 36,448 | **28.4** | 9.3 | 0.1 | 22.5 | 28.4 | 34.5 | 77.3 |
| **Energy: Processed foods -G3 Middle** | **Norway** | 36,448 | **10.7** | 4.7 | 0 | 7.3 | 10.2 | 13.6 | 42.4 |
| **Energy: Processed foods -G3 Upper** | **Norway** | 36,448 | **9.4** | 4.6 | 0 | 6.1 | 8.7 | 12.0 | 42.4 |
| **Energy: Ultra-processed foods -G4 Lower** | **France** | 73,035 | **18.2** | 8.7 | 0.1 | 12.0 | 17.1 | 23.2 | 77.0 |
| **Energy: Ultra-processed foods -G4 Middle** | **France** | 73,035 | **19.4** | 9.1 | 0.1 | 12.9 | 18.3 | 24.7 | 77.0 |
| **Energy: Ultra-processed foods -G4 Upper** | **France** | 73,035 | **36.3** | 10.7 | 0 | 29.2 | 36.3 | 43.3 | 85.4 |
| **Energy: Ultra-processed foods -G4 Lower** | **Italy** | 45,908 | **15.8** | 7.7 | 0 | 10.3 | 14.8 | 20.1 | 68.3 |
| **Energy: Ultra-processed foods -G4 Middle** | **Italy** | 45,908 | **15.8** | 7.7 | 0 | 10.3 | 14.7 | 20.1 | 68.3 |
| **Energy: Ultra-processed foods -G4 Upper** | **Italy** | 45,908 | **38.3** | 9.9 | 3.6 | 31.6 | 38.1 | 44.8 | 84.6 |
| **Energy: Ultra-processed foods -G4 Lower** | **Spain** | 40,621 | **15.6** | 9.5 | 0 | 8.6 | 14.2 | 21.0 | 80.7 |
| **Energy: Ultra-processed foods -G4 Middle** | **Spain** | 40,621 | **15.8** | 9.5 | 0 | 8.8 | 14.4 | 21.2 | 80.7 |
| **Energy: Ultra-processed foods -G4 Upper** | **Spain** | 40,621 | **35.9** | 10.7 | 0 | 28.8 | 35.9 | 43.0 | 83.1 |
| **Energy: Ultra-processed foods -G4 Lower** | **United Kingdom** | 81,097 | **33.2** | 10.6 | 0 | 25.7 | 32.7 | 40.0 | 86.3 |
| **Energy: Ultra-processed foods -G4 Middle** | **United Kingdom** | 81,097 | **46.0** | 11.5 | 0 | 38.2 | 46.0 | 53.8 | 89.8 |
| **Energy: Ultra-processed foods -G4 Upper** | **United Kingdom** | 81,097 | **50.7** | 11.4 | 0 | 43.1 | 51.0 | 58.6 | 89.8 |
| **Energy: Ultra-processed foods -G4 Lower** | **The Netherlands** | 39,036 | **30.2** | 7.5 | 4.1 | 25.2 | 30.0 | 35.0 | 69.5 |
| **Energy: Ultra-processed foods -G4 Middle** | **The Netherlands** | 39,036 | **33.1** | 8.1 | 4.7 | 27.7 | 32.9 | 38.3 | 70.7 |
| **Energy: Ultra-processed foods -G4 Upper** | **The Netherlands** | 39,036 | **50.7** | 8.9 | 7.1 | 44.9 | 50.9 | 56.7 | 89.7 |
| **Energy: Ultra-processed foods -G4 Lower** | **Germany** | 52,013 | **33.8** | 10.3 | 0 | 26.7 | 33.5 | 40.5 | 80.2 |
| **Energy: Ultra-processed foods -G4 Middle** | **Germany** | 52,013 | **34.6** | 10.3 | 0 | 27.5 | 34.3 | 41.4 | 80.8 |
| **Energy: Ultra-processed foods -G4 Upper** | **Germany** | 52,013 | **57.8** | 9.9 | 0 | 51.7 | 58.3 | 64.6 | 93.3 |
| **Energy: Ultra-processed foods -G4 Lower** | **Sweden** | 52,750 | **30.5** | 9.3 | 0 | 23.9 | 29.9 | 36.4 | 76.9 |
| **Energy: Ultra-processed foods -G4 Middle** | **Sweden** | 52,750 | **33.8** | 9.6 | 0 | 27.1 | 33.5 | 40.2 | 77.7 |
| **Energy: Ultra-processed foods -G4 Upper** | **Sweden** | 52,750 | **51.6** | 10.1 | 7.7 | 45.0 | 51.7 | 58.4 | 93.0 |
| **Energy: Ultra-processed foods -G4 Lower** | **Denmark** | 55,860 | **26.5** | 9.6 | 0 | 19.8 | 25.9 | 32.3 | 81.5 |
| **Energy: Ultra-processed foods -G4 Middle** | **Denmark** | 55,860 | **46.4** | 9.9 | 2.5 | 39.8 | 46.3 | 53.1 | 91.9 |
| **Energy: Ultra-processed foods -G4 Upper** | **Denmark** | 55,860 | **52.4** | 10.0 | 3.1 | 45.7 | 52.4 | 59.2 | 93.3 |
|  |  |  |  |  |  |  |  |  |  |
| **Label** | **Country** | **N** | **Mean** | **Std** | **Min** | **P25** | **P50** | **P75** | **Max** |
| **Energy: Ultra-processed foods -G4 Lower** | **Norway** | 36,448 | **25.6** | 7.4 | 0.5 | 20.6 | 25.2 | 30.1 | 68.3 |
| **Energy: Ultra-processed foods -G4 Middle** | **Norway** | 36,448 | **46.8** | 9.0 | 3.2 | 41.0 | 46.9 | 52.8 | 99.1 |
| **Energy: Ultra-processed foods -G4 Upper** | **Norway** | 36,448 | **53.0** | 9.2 | 6.0 | 47.3 | 53.4 | 59.2 | 99.1 |

**Supplementary table 6a:** Characteristics of the study population by sex-specific quartiles of relative intake of NOVA group 1 - Unprocessed or minimally processed foods (% g/day and % kcal/day including alcohol)

| **Characteristics** | **NOVA group 1 quartiles in %g/d** | | | | | | | | **NOVA group 1 quartiles in %kcal/d** | | | | | | | |
| --- | --- | --- | --- | --- | --- | --- | --- | --- | --- | --- | --- | --- | --- | --- | --- | --- |
|  | **1st** | | **2nd** | | **3rd** | | **4th** | | **1st** | | **2nd** | | **3rd** | | **4th** | |
|  | **Mean or N** | **SD %** | **Mean or N** | **SD %** | **Mean or N** | **SD %** | **Mean or N** | **SD or %** | **Mean or N** | **SD %** | **Mean or N** | **SD %** | **Mean or N** | **SD %** | **Mean or N** | **SD or %** |
| **Sex, n %** |  |  |  |  |  |  |  |  |  |  |  |  |  |  |  |  |
| Male | **33,982** | 28.5 | **33,982** | 28.5 | **33,983** | 28.5 | **33,982** | 28.5 | **33,982** | 28.5 | **33,982** | 28.5 | **33,983** | 28.5 | **33,982** | 28.5 |
| Female | **85,209** | 71.5 | **85,210** | 71.5 | **85,210** | 71.5 | **85,210** | 71.5 | **85,209** | 71.5 | **85,210** | 71.5 | **85,210** | 71.5 | **85,210** | 71.5 |
| **Age, years** | **48.7** | 9.9 | **50.5** | 9.9 | **52.2** | 9.7 | **53.9** | 8.9 | **50.6** | 10.0 | **51.3** | 9.8 | **51.5** | 9.6 | **51.8** | 9.7 |
| **Height, cm** | **166.0** | 8.7 | **166.5** | 8.8 | **166.3** | 8.9 | **165.8** | 8.9 | **166.7** | 8.7 | **166.4** | 8.8 | **166.0** | 8.8 | **165.3** | 8.9 |
| **BMI, kg/m^2^** | **25.4** | 4.3 | **25.2** | 4.2 | **25.1** | 4.1 | **25.3** | 4.2 | **25.1** | 4.2 | **25.1** | 4.1 | **25.3** | 4.1 | **25.5** | 4.3 |
| **Education, n %** |  |  |  |  |  |  |  |  |  |  |  |  |  |  |  |  |
| None | **4,301** | 3.6 | **4,108** | 3.4 | **3,715** | 3.1 | **3,820** | 3.2 | **1,389** | 1.2 | **2,634** | 2.2 | **4,320** | 3.6 | **7,601** | 6.4 |
| Primary school completed | **33,447** | 28.1 | **27,997** | 23.5 | **27,067** | 22.7 | **28,894** | 24.2 | **29,318** | 24.6 | **29,702** | 24.9 | **29,612** | 24.8 | **28,773** | 24.1 |
| Technical/professional school | **29,044** | 24.4 | **28,592** | 24.0 | **27,049** | 22.7 | **24,945** | 20.9 | **33,912** | 28.5 | **28,174** | 23.6 | **25,363** | 21.3 | **22,181** | 18.6 |
| Secondary school | **23,211** | 19.5 | **24,722** | 20.7 | **25,596** | 21.5 | **26,248** | 22.0 | **20,735** | 17.4 | **26,297** | 22.1 | **27,167** | 22.8 | **25,578** | 21.5 |
| Longer education | **26,373** | 22.1 | **29,477** | 24.7 | **30,235** | 25.4 | **29,366** | 24.6 | **30,313** | 25.4 | **28,145** | 23.6 | **28,016** | 23.5 | **28,977** | 24.3 |
| Not specified | **2,815** | 2.4 | **4,296** | 3.6 | **5,531** | 4.6 | **5,919** | 5.0 | **3,524** | 3.0 | **4,240** | 3.6 | **4,715** | 4.0 | **6,082** | 5.1 |
| **Smoking status, n %** |  |  |  |  |  |  |  |  |  |  |  |  |  |  |  |  |
| Never | **53,572** | 44.9 | **56,811** | 47.7 | **60,136** | 50.5 | **61,119** | 51.3 | **53,301** | 44.7 | **55,451** | 46.5 | **58,781** | 49.3 | **64,105** | 53.8 |
| Former | **32,031** | 26.9 | **33,177** | 27.8 | **32,944** | 27.6 | **32,472** | 27.2 | **33,488** | 28.1 | **33,588** | 28.2 | **32,683** | 27.4 | **30,865** | 25.9 |
| Current | **31,810** | 26.7 | **26,944** | 22.6 | **23,678** | 19.9 | **22,926** | 19.2 | **30,730** | 25.8 | **27,780** | 23.3 | **25,214** | 21.2 | **21,634** | 18.2 |
| Unknown | **1,778** | 1.5 | **2,260** | 1.9 | **2,435** | 2.0 | **2,675** | 2.2 | **1,672** | 1.4 | **2,373** | 2.0 | **2,515** | 2.1 | **2,588** | 2.2 |
| **Smoking intensity, n %** |  |  |  |  |  |  |  |  |  |  |  |  |  |  |  |  |
| Never | **48,073** | 40.3 | **50,491** | 42.4 | **52,036** | 43.7 | **51,330** | 43.1 | **48,346** | 40.6 | **47,885** | 40.2 | **50,401** | 42.3 | **55,298** | 46.4 |
| Current, 1-15 cig/day | **16,171** | 13.6 | **14,749** | 12.4 | **12,810** | 10.7 | **11,913** | 10.0 | **16,226** | 13.6 | **14,571** | 12.2 | **13,424** | 11.3 | **11,422** | 9.6 |
| Current, 16-25 cig/day | **9,657** | 8.1 | **7,242** | 6.1 | **6,253** | 5.2 | **6,115** | 5.1 | **9,227** | 7.7 | **7,778** | 6.5 | **6,763** | 5.7 | **5,499** | 4.6 |
| Current, 26+ cig/day | **2,811** | 2.4 | **1,553** | 1.3 | **1,202** | 1.0 | **1,306** | 1.1 | **2,396** | 2.0 | **1,798** | 1.5 | **1,427** | 1.2 | **1,251** | 1.0 |
| Former, quit <= 10 years | **12,774** | 10.7 | **11,984** | 10.1 | **10,926** | 9.2 | **10,386** | 8.7 | **11,853** | 9.9 | **11,877** | 10.0 | **11,514** | 9.7 | **10,826** | 9.1 |
| Former, quit 11-20 years | **10,215** | 8.6 | **10,324** | 8.7 | **10,011** | 8.4 | **9,405** | 7.9 | **10,549** | 8.9 | **10,237** | 8.6 | **9,930** | 8.3 | **9,239** | 7.8 |
| Former, quit 20+ years | **8,080** | 6.8 | **9,622** | 8.1 | **10,639** | 8.9 | **11,090** | 9.3 | **9,880** | 8.3 | **10,172** | 8.5 | **9,914** | 8.3 | **9,465** | 7.9 |
| Current, pipe/cigar/occas | **8,213** | 6.9 | **9,248** | 7.8 | **11,226** | 9.4 | **13,459** | 11.3 | **7,378** | 6.2 | **10,787** | 9.1 | **11,776** | 9.9 | **12,205** | 10.2 |
| Current/Former, missing | **3,197** | 2.7 | **3,979** | 3.3 | **4,090** | 3.4 | **4,188** | 3.5 | **3,336** | 2.8 | **4,087** | 3.4 | **4,044** | 3.4 | **3,987** | 3.3 |
| **Physical Activity, n%** |  |  |  |  |  |  |  |  |  |  |  |  |  |  |  |  |
| Inactive | **23,861** | 20.0 | **22,210** | 18.6 | **23,137** | 19.4 | **25,015** | 21.0 | **22,634** | 19.0 | **22,151** | 18.6 | **22,984** | 19.3 | **26,454** | 22.2 |
| Moderately inactive | **40,061** | 33.6 | **39,130** | 32.8 | **39,653** | 33.3 | **40,416** | 33.9 | **41,048** | 34.4 | **39,639** | 33.3 | **39,508** | 33.1 | **39,065** | 32.8 |
| Moderately active | **32,314** | 27.1 | **33,120** | 27.8 | **31,594** | 26.5 | **29,867** | 25.1 | **30,921** | 25.9 | **32,364** | 27.2 | **32,720** | 27.5 | **30,890** | 25.9 |
| Active | **20,638** | 17.3 | **21,817** | 18.3 | **22,261** | 18.7 | **22,264** | 18.7 | **21,981** | 18.4 | **22,172** | 18.6 | **21,735** | 18.2 | **21,092** | 17.7 |
| Missing | **2,317** | 1.9 | **2,915** | 2.4 | **2,548** | 2.1 | **1,630** | 1.4 | **2,607** | 2.2 | **2,866** | 2.4 | **2,246** | 1.9 | **1,691** | 1.4 |
| **Hypertension, n %** |  |  |  |  |  |  |  |  |  |  |  |  |  |  |  |  |
| No | **85,397** | 71.6 | **79,001** | 66.3 | **75,087** | 63.0 | **73,585** | 61.7 | **75,695** | 63.5 | **76,396** | 64.1 | **79,122** | 66.4 | **81,857** | 68.7 |
| Yes | **22,074** | 18.5 | **21,073** | 17.7 | **20,780** | 17.4 | **21,742** | 18.2 | **23,618** | 19.8 | **20,442** | 17.2 | **20,205** | 17.0 | **21,404** | 18.0 |
| Do not know | **11,720** | 9.8 | **19,118** | 16.0 | **23,326** | 19.6 | **23,865** | 20.0 | **19,878** | 16.7 | **22,354** | 18.8 | **19,866** | 16.7 | **15,931** | 13.4 |
| **Hyperlipidaemia, n %** |  |  |  |  |  |  |  |  |  |  |  |  |  |  |  |  |
| No | **70,724** | 59.3 | **66,123** | 55.5 | **66,187** | 55.5 | **67,225** | 56.4 | **69,891** | 58.6 | **66,631** | 55.9 | **66,559** | 55.8 | **67,178** | 56.4 |
| Yes | **17,384** | 14.6 | **14,422** | 12.1 | **12,864** | 10.8 | **13,854** | 11.6 | **17,531** | 14.7 | **13,563** | 11.4 | **13,295** | 11.2 | **14,135** | 11.9 |
| Do not know | **31,083** | 26.1 | **38,647** | 32.4 | **40,142** | 33.7 | **38,113** | 32.0 | **31,769** | 26.7 | **38,998** | 32.7 | **39,339** | 33.0 | **37,879** | 31.8 |
| **Relative Mediteranean diet (**[**21**](#_ENREF_21)**)** |  |  |  |  |  |  |  |  |  |  |  |  |  |  |  |  |
| Low | **32,457** | 27 | **33,769** | 28 | **35,226** | 30 | **33,666** | 28 | **43,220** | 36.3 | **34,778** | 29.2 | **30,674** | 25.7 | **26,446** | 22.2 |
| Medium | **55,604** | 47 | **53,986** | 45 | **53,505** | 45 | **56,069** | 47 | **58,846** | 49.4 | **55,659** | 46.7 | **52,997** | 44.5 | **51,662** | 43.3 |
| High | **31,130** | 26 | **31,437** | 26 | **30,462** | 26 | **29,457** | 25 | **17,125** | 14.4 | **28,755** | 24.1 | **35,522** | 29.8 | **41,084** | 34.5 |
| **FSA-NPS DI Score* (**[**22**](#_ENREF_22)**)** | **6.4** | 2.1 | **6.1** | 2.1 | **6.0** | 2.1 | **5.3** | 2.2 | **7.4** | 1.9 | **6.4** | 1.7 | **5.6** | 1.7 | **4.3** | 1.9 |
| **Energy intake, kcal/d** | **2,230** | 668 | **2,146** | 620 | **2,054** | 579 | **1,855** | 529 | **2197** | 645 | **2143** | 613 | **2060** | 595 | **1885** | 568 |
| **Alcohol intake, g/d** | **20** | 25 | **13** | 15 | **9** | 11 | **6** | 8 | **16** | 22 | **13** | 18 | **11** | 15 | **7** | 11 |
| **Fiber intake, g/d** | **22** | 7 | **23** | 8 | **23** | 8 | **23** | 8 | **22** | 7 | **23** | 7 | **23** | 8 | **24** | 9 |
| **Total fat intake, g/d** | **84** | 31 | **84** | 30 | **80** | 28 | **72** | 26 | **88** | 31 | **84** | 29 | **79** | 28 | **70** | 26 |
| **Carbohydrate intake, g/d** | **244** | 81 | **237** | 75 | **229** | 71 | **208** | 66 | **241** | 80 | **235** | 74 | **228** | 71 | **214** | 70 |
| **Total proteins , g/d** | **88** | 28 | **89** | 28 | **87** | 27 | **84** | 26 | **82** | 26 | **88** | 27 | **90** | 27 | **87** | 29 |
| **Calcium intake, g/d** | **918** | 387 | **985** | 399 | **1,028** | 406 | **1,050** | 438 | **941** | 376 | **998** | 386 | **1016** | 399 | **1026** | 472 |
| **Sodium intake, g/d** | **2,768** | 1,041 | **2,759** | 1,038 | **2,740** | 1,043 | **2,563** | 1,027 | **2887** | 1054 | **2862** | 1035 | **2710** | 1015 | **2370** | 976 |

* a higher FSAm-NPS Dietary Index score reflects an overall lower nutritional quality of consumed foods.

**Supplementary table 6b: Characteristics of the study population by sex-specific quartiles of relative intake of NOVA group 2 - Processed culinary ingredients (% g/day and % kcal/day including alcohol)**

| **Characteristics** | **NOVA group 2 quartiles in %g/d** | | | | | | | | **NOVA group 2 quartiles in %kcal/d** | | | | | | | |
| --- | --- | --- | --- | --- | --- | --- | --- | --- | --- | --- | --- | --- | --- | --- | --- | --- |
|  | **1st** | | **2nd** | | **3rd** | | **4th** | | **1st** | | **2nd** | | **3rd** | | **4th** | |
|  | **Mean or N** | **SD %** | **Mean or N** | **SD %** | **Mean or N** | **SD %** | **Mean or N** | **SD or %** | **Mean or N** | **SD %** | **Mean or N** | **SD %** | **Mean or N** | **SD %** | **Mean or N** | **SD or %** |
| **Sex, n %** |  |  |  |  |  |  |  |  |  |  |  |  |  |  |  |  |
| Male | **33,982** | 28.5 | **33,982** | 28.5 | **33,983** | 28.5 | **33,982** | 28.5 | **33,982** | 28.5 | **33,982** | 28.5 | **33,983** | 28.5 | **33,982** | 28.5 |
| Female | **85,209** | 71.5 | **85,210** | 71.5 | **85,210** | 71.5 | **85,210** | 71.5 | **85,209** | 71.5 | **85,210** | 71.5 | **85,210** | 71.5 | **85,210** | 71.5 |
| **Age, years** | **51.5** | 11.1 | **51.6** | 9.8 | **51.6** | 9.4 | **50.6** | 8.6 | **51.2** | 11.4 | **51.6** | 10.0 | **51.6** | 9.1 | **50.9** | 8.3 |
| **Height, cm** | **167.3** | 8.4 | **167.5** | 8.6 | **166.0** | 8.9 | **163.7** | 8.9 | **167.4** | 8.3 | **167.7** | 8.6 | **165.8** | 9.0 | **163.6** | 8.8 |
| **BMI, kg/m^2^** | **25.6** | 4.3 | **25.2** | 4.1 | **24.8** | 4.1 | **25.4** | 4.3 | **25.4** | 4.2 | **25.2** | 4.1 | **24.9** | 4.1 | **25.5** | 4.3 |
| **Education, n %** |  |  |  |  |  |  |  |  |  |  |  |  |  |  |  |  |
| None | **310** | 0.3 | **1,300** | 1.1 | **4,664** | 3.9 | **9,670** | 8.1 | **290** | 0.2 | **998** | 0.8 | **5,065** | 4.2 | **9,591** | 8.0 |
| Primary school completed | **27,684** | 23.2 | **24,958** | 20.9 | **25,119** | 21.1 | **39,644** | 33.3 | **27,635** | 23.2 | **25,648** | 21.5 | **25,749** | 21.6 | **38,373** | 32.2 |
| Technical/professional school | **39,081** | 32.8 | **32,906** | 27.6 | **21,857** | 18.3 | **15,786** | 13.2 | **37,893** | 31.8 | **35,185** | 29.5 | **21,610** | 18.1 | **14,942** | 12.5 |
| Secondary school | **16,969** | 14.2 | **24,162** | 20.3 | **30,904** | 25.9 | **27,742** | 23.3 | **17,230** | 14.5 | **23,704** | 19.9 | **30,425** | 25.5 | **28,418** | 23.8 |
| Longer education | **27,337** | 22.9 | **31,602** | 26.5 | **32,416** | 27.2 | **24,096** | 20.2 | **27,576** | 23.1 | **29,642** | 24.9 | **32,669** | 27.4 | **25,564** | 21.4 |
| Not specified | **7,810** | 6.6 | **4,264** | 3.6 | **4,233** | 3.6 | **2,254** | 1.9 | **8,567** | 7.2 | **4,015** | 3.4 | **3,675** | 3.1 | **2,304** | 1.9 |
| **Smoking status, n %** |  |  |  |  |  |  |  |  |  |  |  |  |  |  |  |  |
| Never | **52,445** | 44.0 | **55,418** | 46.5 | **62,285** | 52.3 | **61,490** | 51.6 | **55,227** | 46.3 | **54,693** | 45.9 | **60,851** | 51.1 | **60,867** | 51.1 |
| Former | **37,186** | 31.2 | **35,846** | 30.1 | **30,961** | 26.0 | **26,631** | 22.3 | **35,886** | 30.1 | **36,419** | 30.6 | **30,985** | 26.0 | **27,334** | 22.9 |
| Current | **27,894** | 23.4 | **25,458** | 21.4 | **22,796** | 19.1 | **29,210** | 24.5 | **26,126** | 21.9 | **25,833** | 21.7 | **24,541** | 20.6 | **28,858** | 24.2 |
| Unknown | **1,666** | 1.4 | **2,470** | 2.1 | **3,151** | 2.6 | **1,861** | 1.6 | **1,952** | 1.6 | **2,247** | 1.9 | **2,816** | 2.4 | **2,133** | 1.8 |
| **Smoking intensity, n %** |  |  |  |  |  |  |  |  |  |  |  |  |  |  |  |  |
| Never | **51,618** | 43.3 | **50,842** | 42.7 | **50,142** | 42.1 | **49,328** | 41.4 | **54,354** | 45.6 | **50,930** | 42.7 | **48,515** | 40.7 | **48,131** | 40.4 |
| Current, 1-15 cig/day | **14,900** | 12.5 | **14,444** | 12.1 | **12,080** | 10.1 | **14,219** | 11.9 | **14,340** | 12.0 | **14,987** | 12.6 | **12,692** | 10.6 | **13,624** | 11.4 |
| Current, 16-25 cig/day | **7,844** | 6.6 | **6,565** | 5.5 | **6,006** | 5.0 | **8,852** | 7.4 | **7,056** | 5.9 | **6,626** | 5.6 | **6,824** | 5.7 | **8,761** | 7.4 |
| Current, 26+ cig/day | **1,509** | 1.3 | **1,238** | 1.0 | **1,470** | 1.2 | **2,655** | 2.2 | **1,238** | 1.0 | **1,137** | 1.0 | **1,781** | 1.5 | **2,716** | 2.3 |
| Former, quit <= 10 years | **12,572** | 10.5 | **11,797** | 9.9 | **10,707** | 9.0 | **10,994** | 9.2 | **11,957** | 10.0 | **12,148** | 10.2 | **10,873** | 9.1 | **11,092** | 9.3 |
| Former, quit 11-20 years | **10,971** | 9.2 | **10,575** | 8.9 | **9,468** | 7.9 | **8,941** | 7.5 | **10,422** | 8.7 | **10,710** | 9.0 | **9,561** | 8.0 | **9,262** | 7.8 |
| Former, quit 20+ years | **11,924** | 10.0 | **11,940** | 10.0 | **9,514** | 8.0 | **6,053** | 5.1 | **11,788** | 9.9 | **12,100** | 10.2 | **9,301** | 7.8 | **6,242** | 5.2 |
| Current, pipe/cigar/occas | **3,689** | 3.1 | **7,268** | 6.1 | **15,439** | 13.0 | **15,750** | 13.2 | **3,674** | 3.1 | **6,165** | 5.2 | **15,599** | 13.1 | **16,708** | 14.0 |
| Current/Former, missing | **4,164** | 3.5 | **4,523** | 3.8 | **4,367** | 3.7 | **2,400** | 2.0 | **4,362** | 3.7 | **4,389** | 3.7 | **4,047** | 3.4 | **2,656** | 2.2 |
| **Physical Activity, n%** |  |  |  |  |  |  |  |  |  |  |  |  |  |  |  |  |
| Inactive | **19,793** | 16.6 | **18,541** | 15.6 | **23,376** | 19.6 | **32,513** | 27.3 | **20,368** | 17.1 | **18,019** | 15.1 | **23,966** | 20.1 | **31,870** | 26.7 |
| Moderately inactive | **37,380** | 31.4 | **38,091** | 32.0 | **40,943** | 34.4 | **42,846** | 35.9 | **37,472** | 31.4 | **37,680** | 31.6 | **40,933** | 34.3 | **43,175** | 36.2 |
| Moderately active | **30,767** | 25.8 | **35,739** | 30.0 | **33,510** | 28.1 | **26,879** | 22.6 | **32,222** | 27.0 | **35,139** | 29.5 | **32,430** | 27.2 | **27,104** | 22.7 |
| Active | **28,212** | 23.7 | **23,866** | 20.0 | **19,124** | 16.0 | **15,778** | 13.2 | **26,259** | 22.0 | **24,988** | 21.0 | **19,555** | 16.4 | **16,178** | 13.6 |
| Missing | **3,039** | 2.5 | **2,955** | 2.5 | **2,240** | 1.9 | **1,176** | 1.0 | **2,870** | 2.4 | **3,366** | 2.8 | **2,309** | 1.9 | **865** | 0.7 |
| **Hypertension, n %** |  |  |  |  |  |  |  |  |  |  |  |  |  |  |  |  |
| No | **66,613** | 55.9 | **73,944** | 62.0 | **81,530** | 68.4 | **90,983** | 76.3 | **68,246** | 57.3 | **69,916** | 58.7 | **82,874** | 69.5 | **92,034** | 77.2 |
| Yes | **21,691** | 18.2 | **21,619** | 18.1 | **21,138** | 17.7 | **21,221** | 17.8 | **19,489** | 16.4 | **22,196** | 18.6 | **21,867** | 18.3 | **22,117** | 18.6 |
| Do not know | **30,887** | 25.9 | **23,629** | 19.8 | **16,525** | 13.9 | **6,988** | 5.9 | **31,456** | 26.4 | **27,080** | 22.7 | **14,452** | 12.1 | **5,041** | 4.2 |
| **Hyperlipidaemia, n %** |  |  |  |  |  |  |  |  |  |  |  |  |  |  |  |  |
| No | **61,118** | 51.3 | **54,598** | 45.8 | **69,771** | 58.5 | **84,772** | 71.1 | **55,580** | 46.6 | **52,090** | 43.7 | **75,045** | 63.0 | **87,544** | 73.4 |
| Yes | **10,862** | 9.1 | **12,364** | 10.4 | **15,745** | 13.2 | **19,553** | 16.4 | **8,263** | 6.9 | **11,958** | 10.0 | **17,297** | 14.5 | **21,006** | 17.6 |
| Do not know | **47,211** | 39.6 | **52,230** | 43.8 | **33,677** | 28.3 | **14,867** | 12.5 | **55,348** | 46.4 | **55,144** | 46.3 | **26,851** | 22.5 | **10,642** | 8.9 |
| **Relative Mediteranean diet (**[**21**](#_ENREF_21)**)** |  |  |  |  |  |  |  |  |  |  |  |  |  |  |  |  |
| Low | **41,391** | 35 | **39,798** | 33 | **33,355** | 28 | **20,574** | 17 | **41,799** | 35.1 | **42,740** | 35.9 | **33,362** | 28.0 | **17,217** | 14.4 |
| Medium | **52,211** | 44 | **58,886** | 49 | **56,959** | 48 | **51,108** | 43 | **51,235** | 43.0 | **58,755** | 49.3 | **57,934** | 48.6 | **51,240** | 43.0 |
| High | **25,589** | 21 | **20,508** | 17 | **28,879** | 24 | **47,510** | 40 | **26,157** | 21.9 | **17,697** | 14.8 | **27,897** | 23.4 | **50,735** | 42.6 |
| **FSA-NPS DI Score* (**[**22**](#_ENREF_22)**)** | **5.4** | 2.2 | **5.9** | 2.0 | **6.3** | 2.1 | **6.1** | 2.1 | **5.6** | 2.3 | **5.9** | 2.0 | **6.4** | 2.0 | **5.9** | 2.1 |
| **Energy intake, kcal/d** | **1,912** | 563 | **1,997** | 592 | **2,133** | 619 | **2,244** | 639 | **1951** | 575 | **2022** | 603 | **2150** | 628 | **2163** | 636 |
| **Alcohol intake, g/d** | **13** | 18 | **12** | 17 | **12** | 17 | **11** | 16 | **11** | 17 | **12** | 17 | **13** | 18 | **12** | 17 |
| **Fiber intake, g/d** | **23** | 8 | **23** | 8 | **23** | 8 | **23** | 8 | **24** | 8 | **23** | 8 | **22** | 7 | **23** | 8 |
| **Total fat intake, g/d** | **70** | 26 | **76** | 27 | **84** | 29 | **90** | 30 | **72** | 28 | **77** | 28 | **84** | 29 | **88** | 29 |
| **Carbohydrate intake, g/d** | **218** | 68 | **223** | 71 | **233** | 76 | **245** | 80 | **224** | 69 | **228** | 73 | **235** | 77 | **232** | 79 |
| **Total proteins , g/d** | **82** | 25 | **84** | 26 | **89** | 28 | **93** | 29 | **83** | 26 | **85** | 26 | **90** | 28 | **91** | 29 |
| **Calcium intake, g/d** | **1,037** | 415 | **987** | 416 | **1,001** | 417 | **957** | 391 | **1027** | 411 | **1002** | 428 | **1017** | 427 | **935** | 369 |
| **Sodium intake, g/d** | **2,726** | 1,061 | **2,701** | 1,063 | **2,765** | 1,040 | **2,636** | 994 | **2780** | 1084 | **2730** | 1053 | **2791** | 1047 | **2529** | 953 |

* a higher FSAm-NPS Dietary Index score reflects an overall lower nutritional quality of consumed foods.

**Supplementary table 6c: Characteristics of the study population by sex-specific quartiles of relative intake of NOVA group 3 - Processed foods (% g/day and % kcal/day including alcohol)**

| **Characteristics** | **NOVA group 3 quartiles in %g/d** | | | | | | | | **NOVA group 3 quartiles in %kcal/d** | | | | | | | |
| --- | --- | --- | --- | --- | --- | --- | --- | --- | --- | --- | --- | --- | --- | --- | --- | --- |
|  | **1st** | | **2nd** | | **3rd** | | **4th** | | **1st** | | **2nd** | | **3rd** | | **4th** | |
|  | **Mean or N** | **SD %** | **Mean or N** | **SD %** | **Mean or N** | **SD %** | **Mean or N** | **SD or %** | **Mean or N** | **SD %** | **Mean or N** | **SD %** | **Mean or N** | **SD %** | **Mean or N** | **SD or %** |
| **Sex, n %** |  |  |  |  |  |  |  |  |  |  |  |  |  |  |  |  |
| Male | **33,982** | 28.5 | **33,982** | 28.5 | **33,983** | 28.5 | **33,982** | 28.5 | **33,982** | 28.5 | **33,982** | 28.5 | **33,983** | 28.5 | **33,982** | 28.5 |
| Female | **85,209** | 71.5 | **85,210** | 71.5 | **85,210** | 71.5 | **85,210** | 71.5 | **85,209** | 71.5 | **85,210** | 71.5 | **85,210** | 71.5 | **85,210** | 71.5 |
| **Age, years** | **52.3** | 11.0 | **51.2** | 10.0 | **50.9** | 9.4 | **50.9** | 8.6 | **50.8** | 11.0 | **51.4** | 10.3 | **51.4** | 9.3 | **51.5** | 8.3 |
| **Height, cm** | **166.6** | 8.6 | **167.1** | 8.8 | **166.3** | 8.8 | **164.6** | 8.8 | **167.2** | 8.5 | **166.7** | 8.9 | **165.8** | 9.0 | **164.9** | 8.7 |
| **BMI, kg/m^2^** | **25.5** | 4.4 | **25.1** | 4.1 | **25.0** | 4.1 | **25.4** | 4.2 | **25.3** | 4.3 | **25.2** | 4.1 | **25.3** | 4.2 | **25.2** | 4.2 |
| **Education, n %** |  |  |  |  |  |  |  |  |  |  |  |  |  |  |  |  |
| None | **1,684** | 1.4 | **2,519** | 2.1 | **4,613** | 3.9 | **7,128** | 6.0 | **2,199** | 1.8 | **3,428** | 2.9 | **4,965** | 4.2 | **5,352** | 4.5 |
| Primary school completed | **28,832** | 24.2 | **26,798** | 22.5 | **26,339** | 22.1 | **35,436** | 29.7 | **26,862** | 22.5 | **29,114** | 24.4 | **30,512** | 25.6 | **30,917** | 25.9 |
| Technical/professional school | **33,322** | 28.0 | **30,406** | 25.5 | **25,376** | 21.3 | **20,526** | 17.2 | **35,151** | 29.5 | **29,343** | 24.6 | **24,879** | 20.9 | **20,257** | 17.0 |
| Secondary school | **20,348** | 17.1 | **26,402** | 22.2 | **27,753** | 23.3 | **25,274** | 21.2 | **19,736** | 16.6 | **24,116** | 20.2 | **27,315** | 22.9 | **28,610** | 24.0 |
| Longer education | **24,695** | 20.7 | **29,012** | 24.3 | **32,585** | 27.3 | **29,159** | 24.5 | **25,061** | 21.0 | **28,637** | 24.0 | **29,363** | 24.6 | **32,390** | 27.2 |
| Not specified | **10,310** | 8.6 | **4,055** | 3.4 | **2,527** | 2.1 | **1,669** | 1.4 | **10,182** | 8.5 | **4,554** | 3.8 | **2,159** | 1.8 | **1,666** | 1.4 |
| **Smoking status, n %** |  |  |  |  |  |  |  |  |  |  |  |  |  |  |  |  |
| Never | **58,123** | 48.8 | **58,956** | 49.5 | **59,486** | 49.9 | **55,073** | 46.2 | **55,867** | 46.9 | **58,119** | 48.8 | **59,000** | 49.5 | **58,652** | 49.2 |
| Former | **32,837** | 27.5 | **33,480** | 28.1 | **32,873** | 27.6 | **31,434** | 26.4 | **33,790** | 28.3 | **32,783** | 27.5 | **31,986** | 26.8 | **32,065** | 26.9 |
| Current | **25,592** | 21.5 | **24,260** | 20.4 | **24,485** | 20.5 | **31,021** | 26.0 | **26,415** | 22.2 | **26,131** | 21.9 | **26,373** | 22.1 | **26,439** | 22.2 |
| Unknown | **2,639** | 2.2 | **2,496** | 2.1 | **2,349** | 2.0 | **1,664** | 1.4 | **3,119** | 2.6 | **2,159** | 1.8 | **1,834** | 1.5 | **2,036** | 1.7 |
| **Smoking intensity, n %** |  |  |  |  |  |  |  |  |  |  |  |  |  |  |  |  |
| Never | **54,219** | 45.5 | **52,286** | 43.9 | **50,418** | 42.3 | **45,007** | 37.8 | **53,840** | 45.2 | **52,225** | 43.8 | **49,534** | 41.6 | **46,331** | 38.9 |
| Current, 1-15 cig/day | **13,709** | 11.5 | **13,771** | 11.6 | **13,242** | 11.1 | **14,921** | 12.5 | **14,495** | 12.2 | **14,296** | 12.0 | **13,844** | 11.6 | **13,008** | 10.9 |
| Current, 16-25 cig/day | **7,316** | 6.1 | **6,255** | 5.2 | **6,376** | 5.3 | **9,320** | 7.8 | **7,177** | 6.0 | **7,028** | 5.9 | **7,323** | 6.1 | **7,739** | 6.5 |
| Current, 26+ cig/day | **1,355** | 1.1 | **1,115** | 0.9 | **1,462** | 1.2 | **2,940** | 2.5 | **1,180** | 1.0 | **1,377** | 1.2 | **1,829** | 1.5 | **2,486** | 2.1 |
| Former, quit <= 10 years | **10,921** | 9.2 | **11,459** | 9.6 | **11,433** | 9.6 | **12,257** | 10.3 | **11,789** | 9.9 | **11,363** | 9.5 | **11,413** | 9.6 | **11,505** | 9.7 |
| Former, quit 11-20 years | **9,111** | 7.6 | **9,819** | 8.2 | **10,376** | 8.7 | **10,649** | 8.9 | **9,148** | 7.7 | **9,519** | 8.0 | **10,444** | 8.8 | **10,844** | 9.1 |
| Former, quit 20+ years | **11,131** | 9.3 | **10,888** | 9.1 | **9,826** | 8.2 | **7,586** | 6.4 | **11,143** | 9.3 | **10,398** | 8.7 | **9,152** | 7.7 | **8,738** | 7.3 |
| Current, pipe/cigar/occas | **6,593** | 5.5 | **9,421** | 7.9 | **12,262** | 10.3 | **13,870** | 11.6 | **4,972** | 4.2 | **8,931** | 7.5 | **12,581** | 10.6 | **15,662** | 13.1 |
| Current/Former, missing | **4,836** | 4.1 | **4,178** | 3.5 | **3,798** | 3.2 | **2,642** | 2.2 | **5,447** | 4.6 | **4,055** | 3.4 | **3,073** | 2.6 | **2,879** | 2.4 |
| **Physical Activity, n%** |  |  |  |  |  |  |  |  |  |  |  |  |  |  |  |  |
| Inactive | **24,998** | 21.0 | **19,245** | 16.1 | **21,324** | 17.9 | **28,656** | 24.0 | **22,456** | 18.8 | **21,400** | 18.0 | **23,742** | 19.9 | **26,625** | 22.3 |
| Moderately inactive | **37,302** | 31.3 | **38,183** | 32.0 | **40,638** | 34.1 | **43,137** | 36.2 | **35,946** | 30.2 | **39,232** | 32.9 | **40,751** | 34.2 | **43,331** | 36.4 |
| Moderately active | **30,613** | 25.7 | **34,954** | 29.3 | **33,577** | 28.2 | **27,751** | 23.3 | **35,515** | 29.8 | **32,669** | 27.4 | **29,690** | 24.9 | **29,021** | 24.3 |
| Active | **23,245** | 19.5 | **23,739** | 19.9 | **21,344** | 17.9 | **18,652** | 15.6 | **22,248** | 18.7 | **23,590** | 19.8 | **22,379** | 18.8 | **18,763** | 15.7 |
| Missing | **3,033** | 2.5 | **3,071** | 2.6 | **2,310** | 1.9 | **996** | 0.8 | **3,026** | 2.5 | **2,301** | 1.9 | **2,631** | 2.2 | **1,452** | 1.2 |
| **Hypertension, n %** |  |  |  |  |  |  |  |  |  |  |  |  |  |  |  |  |
| No | **69,278** | 58.1 | **75,379** | 63.2 | **80,569** | 67.6 | **87,844** | 73.7 | **76,256** | 64.0 | **76,135** | 63.9 | **75,409** | 63.3 | **85,270** | 71.5 |
| Yes | **19,950** | 16.7 | **20,654** | 17.3 | **21,806** | 18.3 | **23,259** | 19.5 | **16,769** | 14.1 | **19,802** | 16.6 | **23,555** | 19.8 | **25,543** | 21.4 |
| Do not know | **29,963** | 25.1 | **23,159** | 19.4 | **16,818** | 14.1 | **8,089** | 6.8 | **26,166** | 22.0 | **23,255** | 19.5 | **20,229** | 17.0 | **8,379** | 7.0 |
| **Hyperlipidaemia, n %** |  |  |  |  |  |  |  |  |  |  |  |  |  |  |  |  |
| No | **57,720** | 48.4 | **59,985** | 50.3 | **69,836** | 58.6 | **82,718** | 69.4 | **51,148** | 42.9 | **60,095** | 50.4 | **74,211** | 62.3 | **84,805** | 71.1 |
| Yes | **9,797** | 8.2 | **11,398** | 9.6 | **15,582** | 13.1 | **21,747** | 18.2 | **7,698** | 6.5 | **10,445** | 8.8 | **16,681** | 14.0 | **23,700** | 19.9 |
| Do not know | **51,674** | 43.4 | **47,809** | 40.1 | **33,775** | 28.3 | **14,727** | 12.4 | **60,345** | 50.6 | **48,652** | 40.8 | **28,301** | 23.7 | **10,687** | 9.0 |
| **Relative Mediteranean diet (**[**21**](#_ENREF_21)**)** |  |  |  |  |  |  |  |  |  |  |  |  |  |  |  |  |
| Low | **42,014** | 35 | **39,907** | 33 | **30,544** | 26 | **22,653** | 19 | **34,240** | 28.7 | **39,634** | 33.3 | **37,544** | 31.5 | **23,700** | 19.9 |
| Medium | **53,829** | 45 | **54,310** | 46 | **56,973** | 48 | **54,052** | 45 | **56,504** | 47.4 | **52,120** | 43.7 | **52,845** | 44.3 | **57,695** | 48.4 |
| High | **23,348** | 20 | **24,975** | 21 | **31,676** | 27 | **42,487** | 36 | **28,447** | 23.9 | **27,438** | 23.0 | **28,804** | 24.2 | **37,797** | 31.7 |
| **FSA-NPS DI Score* (**[**22**](#_ENREF_22)**)** | **5.6** | 2.3 | **6.0** | 2.1 | **6.1** | 2.0 | **6.0** | 2.0 | **5.5** | 2.4 | **6.0** | 2.1 | **6.1** | 2.0 | **6.1** | 1.9 |
| **Energy intake, kcal/d** | **1,895** | 571 | **2,014** | 584 | **2,116** | 609 | **2,260** | 643 | **1929** | 583 | **2031** | 598 | **2123** | 613 | **2202** | 640 |
| **Alcohol intake, g/d** | **4** | 6 | **7** | 9 | **12** | 13 | **25** | 25 | **5** | 7 | **10** | 12 | **13** | 16 | **20** | 24 |
| **Fiber intake, g/d** | **23** | 9 | **23** | 8 | **23** | 7 | **22** | 7 | **23** | 9 | **23** | 8 | **22** | 7 | **23** | 7 |
| **Total fat intake, g/d** | **72** | 28 | **79** | 29 | **84** | 30 | **85** | 29 | **73** | 28 | **80** | 29 | **84** | 29 | **83** | 29 |
| **Carbohydrate intake, g/d** | **222** | 74 | **227** | 72 | **232** | 73 | **237** | 79 | **225** | 73 | **225** | 72 | **230** | 73 | **238** | 79 |
| **Total proteins , g/d** | **82** | 26 | **85** | 26 | **88** | 27 | **93** | 29 | **83** | 26 | **85** | 26 | **88** | 27 | **91** | 29 |
| **Calcium intake, g/d** | **1,031** | 429 | **1,011** | 418 | **987** | 398 | **953** | 394 | **961** | 427 | **1016** | 413 | **1007** | 398 | **996** | 403 |
| **Sodium intake, g/d** | **2,565** | 1,048 | **2,669** | 1,019 | **2,759** | 1,016 | **2,837** | 1,059 | **2613** | 1088 | **2639** | 1044 | **2667** | 960 | **2911** | 1040 |

* a higher FSAm-NPS Dietary Index score reflects an overall lower nutritional quality of consumed foods.

**Supplementary table 7a:** The contributions of the different EPIC food groups to the four NOVA categories expressed in kcal/day for the middle bound (MB) scenario.

|  | **N** | **NOVA G1 (kcal)** | | **NOVA G2 (kcal)** | | **NOVA G3 (kcal)** | | **NOVA G3  excl. ALC (kcal)** | | **NOVA G4 (kcal)** | | **NOVA G4  excl. ALC (kcal)** | |
| --- | --- | --- | --- | --- | --- | --- | --- | --- | --- | --- | --- | --- | --- |
| **Epic Food Classification - MB** |  | **%** | **Order** | **%** | **Order** | **%** | **Order** | **%** | **Order** | **%** | **Order** | **%** | **Order** |
| **Potatoes and other tubers** | 472,088 | 9.26 | . | 0.01 | . | 0.36 | . | 0.44 | . | 1.07 | . | 1.10 | . |
| **Potatoes** | 472,086 | 9.26 | 4 | 0.01 | . | 0.36 | . | 0.44 | 15 | 1.07 | . | 1.10 | . |
| **Other tubers** | 42 | 0.00 | . | 0.00 | . | 0.00 | . | 0.00 | . | 0.00 | . | 0.00 | . |
| **Vegetables** | 476,679 | 8.66 | . | 0.00 | . | 0.60 | . | 0.73 | . | 0.19 | . | 0.19 | . |
| **Vegetables n.s.** | 87,359 | 0.70 | . | 0.00 | . | 0.10 | . | 0.12 | . | 0.00 | . | 0.00 | . |
| **Leafy vegetables** | 422,682 | 0.63 | . | 0.00 | . | 0.00 | . | 0.00 | . | 0.00 | . | 0.00 | . |
| **Fruiting vegetables** | 472,318 | 2.67 | 12 | 0.00 | . | 0.12 | . | 0.15 | . | 0.06 | . | 0.06 | . |
| **Root vegetables** | 446,546 | 1.27 | . | 0.00 | . | 0.21 | . | 0.26 | . | 0.00 | . | 0.01 | . |
| **Cabbages** | 420,040 | 0.98 | . | 0.00 | . | 0.02 | . | 0.02 | . | 0.00 | . | 0.00 | . |
| **Mushrooms** | 416,387 | 0.18 | . | 0.00 | . | 0.01 | . | 0.01 | . | 0.02 | . | 0.02 | . |
| **Grain and pod vegetables** | 383,627 | 0.95 | . | 0.00 | . | 0.07 | . | 0.09 | . | 0.00 | . | 0.00 | . |
| **Onion, garlic** | 459,500 | 0.62 | . | 0.00 | . | 0.02 | . | 0.02 | . | 0.07 | . | 0.07 | . |
| **Stalk vegetables, sprouts** | 409,620 | 0.33 | . | 0.00 | . | 0.00 | . | 0.00 | . | 0.03 | . | 0.03 | . |
| **Mixed salad, mixed vegetables** | 208,468 | 0.33 | . | 0.00 | . | 0.05 | . | 0.06 | . | 0.00 | . | 0.00 | . |
| **Legumes** | 367,815 | 1.66 | . | 0.00 | . | 0.83 | . | 1.01 | . | 0.47 | . | 0.48 | . |
| **Legumes clas.** | 367,815 | 1.66 | 15 | 0.00 | . | 0.83 | 12 | 1.01 | 10 | 0.47 | . | 0.48 | . |
| **Fruits, nuts and seeds** | 474,836 | 18.02 | **2** | 0.07 | . | 2.98 | . | 3.61 | . | 0.14 | . | 0.14 | . |
| **Fruit peel** | 7 | 0.00 | . | 0.00 | . | 0.00 | . | 0.00 | . | 0.00 | . | 0.00 | . |
| **Fruit** | 472,958 | 16.17 | **1** | 0.02 | . | 0.80 | 13 | 0.97 | 11 | 0.02 | . | 0.02 | . |
| **Nuts (-spread) and seeds** | 363,751 | 1.65 | . | 0.00 | . | 1.86 | 8 | 2.26 | 6 | 0.07 | . | 0.08 | . |
| **Mixed fruits** | 102,935 | 0.20 | . | 0.05 | 15 | 0.15 | . | 0.19 | . | 0.02 | . | 0.02 | . |
| **Olives** | 86,723 | 0.00 | . | 0.00 | . | 0.17 | . | 0.20 | . | 0.02 | . | 0.02 | . |

|  | **N** | **NOVA G1 (kcal)** | | **NOVA G2 (kcal)** | | **NOVA G3 (kcal)** | | **NOVA G3  excl. ALC (kcal)** | | **NOVA G4 (kcal)** | | **NOVA G4  excl. ALC (kcal)** | |
| --- | --- | --- | --- | --- | --- | --- | --- | --- | --- | --- | --- | --- | --- |
| **Epic Food Classification - MB** |  | **%** | **Order** | **%** | **Order** | **%** | **Order** | **%** | **Order** | **%** | **Order** | **%** | **Order** |
| **Dairy products** | 476,067 | 18.67 | **1** | 3.38 | . | 23.59 | **2** | 28.64 | **2** | 8.51 | . | 8.73 | . |
| **Dairy product n.s. or combined** | 107,002 | 0.87 | . | 0.00 | . | 0.00 | . | 0.00 | . | 0.59 | . | 0.61 | . |
| **Milk** | 424,317 | 12.48 | **2** | 0.00 | . | 0.18 | . | 0.21 | . | 0.07 | . | 0.07 | . |
| **Milk beverages** | 117,061 | 0.02 | . | 0.00 | . | 0.14 | . | 0.17 | . | 0.83 | . | 0.85 | . |
| **Yoghurt, thick fermented milk** | 386,718 | 2.95 | 11 | 0.00 | . | 0.08 | . | 0.10 | . | 2.88 | 10 | 2.96 | 10 |
| **Curd** | 250,834 | 2.03 | 13 | 0.00 | . | 1.12 | 10 | 1.36 | 8 | 0.28 | . | 0.29 | . |
| **Cheese** | 464,655 | 0.00 | . | 0.00 | . | 21.35 | **2** | 25.92 | **2** | 1.87 | 14 | 1.92 | 14 |
| **Cream desserts, puddings (milk based)** | 289,668 | 0.04 | . | 0.10 | 13 | 0.51 | 15 | 0.62 | 13 | 1.74 | 15 | 1.79 | 15 |
| **Dairy creams** | 372,196 | 0.00 | . | 3.28 | 5 | 0.14 | . | 0.17 | . | 0.22 | . | 0.23 | . |
| **Milk for coffee and creamers (dairy)** | 41,916 | 0.28 | . | 0.00 | . | 0.07 | . | 0.09 | . | 0.02 | . | 0.02 | . |
| **Cereal and cereal products** | 476,652 | 15.04 | **3** | 0.11 | . | 39.88 | **1** | 48.41 | **1** | 26.60 | **1** | 27.29 | **1** |
| **Flour, flakes, starches, semolina** | 201,868 | 0.35 | . | 0.06 | 14 | 0.02 | . | 0.02 | . | 0.17 | . | 0.17 | . |
| **Pasta, rice, other grains** | 461,031 | 11.66 | **3** | 0.02 | . | 0.00 | . | 0.00 | . | 0.38 | . | 0.39 | . |
| **Bread, crispbread, rusks** | 475,509 | 0.00 | . | 0.00 | . | 39.27 | **1** | 47.67 | **1** | 19.83 | **1** | 20.34 | **1** |
| **Breakfast cereals** | 226,520 | 3.01 | 10 | 0.00 | . | 0.00 | . | 0.00 | . | 2.89 | 9 | 2.97 | 9 |
| **Salty biscuits, aperitif biscuits, crackers** | 340,308 | 0.01 | . | 0.00 | . | 0.00 | . | 0.00 | . | 2.40 | 11 | 2.46 | 11 |
| **Dough and pastry (puff, short-crust, pizza)** | 311,065 | 0.02 | . | 0.03 | . | 0.59 | 14 | 0.71 | 12 | 0.93 | . | 0.95 | . |
| **Meat and meat products** | 475,705 | 13.72 | . | 0.00 | . | 2.28 | . | 2.77 | . | 11.61 | . | 11.91 | . |
| **Meat and meat product n.s. or combined** | 58,477 | 0.41 | . | 0.00 | . | 0.02 | . | 0.03 | . | 0.01 | . | 0.01 | . |
| **Red meat** | 466,661 | 9.00 | 5 | 0.00 | . | 0.01 | . | 0.02 | . | 0.78 | . | 0.80 | . |
| **Poultry** | 412,329 | 3.62 | 6 | 0.00 | . | 0.00 | . | 0.00 | . | 0.01 | . | 0.01 | . |
| **Game** | 25,595 | 0.03 | . | 0.00 | . | 0.00 | . | 0.00 | . | 0.00 | . | 0.00 | . |
| **Processed meat** | 469,869 | 0.17 | . | 0.00 | . | 2.24 | 7 | 2.72 | 5 | 10.79 | **2** | 11.07 | **2** |
| **Offals** | 252,743 | 0.49 | . | 0.00 | . | 0.00 | . | 0.00 | . | 0.01 | . | 0.01 | . |
| **Fish and shellfish** | 451,668 | 3.88 | . | 0.00 | . | 2.06 | . | 2.50 | . | 1.22 | . | 1.25 | . |
| **Fish and shellfish n.s. or combined** | 7,620 | 0.08 | . | 0.00 | . | 0.00 | . | 0.00 | . | 0.00 | . | 0.00 | . |
| **Fish** | 445,215 | 3.24 | 8 | 0.00 | . | 1.54 | 9 | 1.87 | 7 | 0.43 | . | 0.44 | . |
| **Crustaceans, molluscs** | 326,942 | 0.46 | . | 0.00 | . | 0.02 | . | 0.02 | . | 0.02 | . | 0.02 | . |
|  | **N** | **NOVA G1 (kcal)** | | **NOVA G2 (kcal)** | | **NOVA G3 (kcal)** | | **NOVA G3  excl. ALC (kcal)** | | **NOVA G4 (kcal)** | | **NOVA G4  excl. ALC (kcal)** | |
| **Epic Food Classification - MB** |  | **%** | **Order** | **%** | **Order** | **%** | **Order** | **%** | **Order** | **%** | **Order** | **%** | **Order** |
| **Fish products, fish in crumbs** | 287,627 | 0.09 | . | 0.00 | . | 0.50 | . | 0.61 | 14 | 0.78 | . | 0.80 | . |
| **Egg and egg products** | 468,194 | 3.55 | . | 0.00 | . | 0.00 | . | 0.00 | . | 0.12 | . | 0.13 | . |
| **Eggs** | 468,194 | 3.55 | 7 | 0.00 | . | 0.00 | . | 0.00 | . | 0.12 | . | 0.13 | . |
| **Fat** | 476,714 | 0.00 | . | 64.80 | **1** | 0.05 | . | 0.06 | . | 12.29 | **3** | 12.61 | **3** |
| **Fat n.s. or combined** | 203,568 | 0.00 | . | 2.15 | 6 | 0.00 | . | 0.00 | . | 0.92 | . | 0.94 | . |
| **Vegetable oils** | 435,880 | 0.00 | . | 41.35 | **1** | 0.02 | . | 0.02 | . | 0.29 | . | 0.29 | . |
| **Butter** | 333,189 | 0.00 | . | 19.20 | **3** | 0.00 | . | 0.00 | . | 0.01 | . | 0.01 | . |
| **Margarine** | 393,553 | 0.00 | . | 0.00 | . | 0.02 | . | 0.02 | . | 10.61 | **3** | 10.88 | **3** |
| **Deep frying fats** | 84,134 | 0.00 | . | 0.17 | 11 | 0.01 | . | 0.01 | . | 0.43 | . | 0.44 | . |
| **Marine oils** | 10,906 | 0.00 | . | 0.72 | 9 | 0.00 | . | 0.00 | . | 0.00 | . | 0.00 | . |
| **Other animal fat** | 95,619 | 0.00 | . | 1.21 | 8 | 0.00 | . | 0.00 | . | 0.03 | . | 0.03 | . |
| **Sugar and confectionary** | 465,456 | 0.00 | . | 20.24 | **2** | 4.39 | . | 5.33 | **3** | 10.57 | . | 10.84 | . |
| **Sugar, honey, jam and syrup** | 435,145 | 0.00 | . | 20.23 | **2** | 4.35 | 5 | 5.28 | **3** | 0.15 | . | 0.15 | . |
| **Chocolate, candy bars, paste, confetti** | 381,589 | 0.00 | . | 0.00 | . | 0.00 | . | 0.00 | . | 5.94 | 5 | 6.09 | 5 |
| **Confectionery non-chocolate, candied fruits** | 287,066 | 0.00 | . | 0.00 | . | 0.04 | . | 0.04 | . | 2.14 | 13 | 2.20 | 13 |
| **Ice cream, water ice** | 397,842 | 0.00 | . | 0.01 | . | 0.00 | . | 0.00 | . | 2.34 | 12 | 2.40 | 12 |
| **Cakes and biscuits** | 453,549 | 0.95 | . | 1.39 | . | 3.96 | . | 4.81 | . | 13.19 | **2** | 13.53 | **2** |
| **Cakes, sweet pies, pastries, puddings** | 440,595 | 0.95 | . | 1.38 | 7 | 3.95 | 6 | 4.80 | 4 | 9.30 | 4 | 9.54 | 4 |
| **Dry cakes, biscuits** | 335,297 | 0.00 | . | 0.01 | . | 0.01 | . | 0.01 | . | 3.89 | 7 | 3.99 | 7 |
| **Non alcoholic beverages** | 474,961 | 4.24 | . | 0.01 | . | 0.05 | . | 0.05 | . | 5.55 | . | 5.70 | . |
| **Non alcoholic beverages n.s.** | 145,175 | 0.00 | . | 0.01 | . | 0.01 | . | 0.01 | . | 0.16 | . | 0.16 | . |
| **Fruit and vegetable juices** | 375,487 | 3.12 | 9 | 0.00 | . | 0.04 | . | 0.04 | . | 0.96 | . | 0.99 | . |
| **Carbonated/soft/isotonic drinks, diluted syrups** | 298,340 | 0.00 | . | 0.00 | . | 0.00 | . | 0.00 | . | 4.43 | 6 | 4.55 | 6 |
| **Coffee, tea, herbal teas** | 466,677 | 1.07 | . | 0.00 | . | 0.00 | . | 0.00 | . | 0.00 | . | 0.00 | . |
| **Waters** | 303,997 | 0.05 | . | 0.00 | . | 0.00 | . | 0.00 | . | 0.00 | . | 0.00 | . |

|  | **N** | **NOVA G1 (kcal)** | | **NOVA G2 (kcal)** | | **NOVA G3 (kcal)** | | **NOVA G3  excl. ALC (kcal)** | | **NOVA G4 (kcal)** | | **NOVA G4  excl. ALC (kcal)** | |
| --- | --- | --- | --- | --- | --- | --- | --- | --- | --- | --- | --- | --- | --- |
| **Epic Food Classification - MB** |  | **%** | **Order** | **%** | **Order** | **%** | **Order** | **%** | **Order** | **%** | **Order** | **%** | **Order** |
| **Alcoholic beverages** | 422,090 | 0.00 | . | 0.00 | . | 17.61 | **3** | 0.00 | . | 2.54 | . | 0.03 | . |
| **Alcoholic beverages n.s. or combined** | 13,067 | 0.00 | . | 0.00 | . | 0.00 | . | 0.00 | . | 0.02 | . | 0.00 | . |
| **Wine** | 381,570 | 0.00 | . | 0.00 | . | 11.04 | **3** | 0.00 | . | 0.00 | . | 0.00 | . |
| **Fortified wines** | 238,590 | 0.00 | . | 0.00 | . | 0.00 | . | 0.00 | . | 1.01 | . | 0.00 | . |
| **Beer, cider** | 311,346 | 0.00 | . | 0.00 | . | 6.57 | 4 | 0.00 | . | 0.03 | . | 0.03 | . |
| **Spirits, brandy** | 247,318 | 0.00 | . | 0.00 | . | 0.00 | . | 0.00 | . | 1.27 | . | 0.00 | . |
| **Aniseed drinks (pastis,...)** | 41,885 | 0.00 | . | 0.00 | . | 0.00 | . | 0.00 | . | 0.05 | . | 0.00 | . |
| **Liqueurs** | 48,356 | 0.00 | . | 0.00 | . | 0.00 | . | 0.00 | . | 0.07 | . | 0.00 | . |
| **Cocktails, punches** | 22,545 | 0.00 | . | 0.00 | . | 0.00 | . | 0.00 | . | 0.08 | . | 0.00 | . |
| **Condiments and sauces** | 463,782 | 0.21 | . | 9.37 | **3** | 0.90 | . | 1.09 | . | 3.79 | . | 3.88 | . |
| **Sauces** | 461,139 | 0.21 | . | 9.35 | 4 | 0.90 | 11 | 1.09 | 9 | 3.69 | 8 | 3.79 | 8 |
| **Yeast** | 3,878 | 0.00 | . | 0.01 | . | 0.00 | . | 0.00 | . | 0.00 | . | 0.00 | . |
| **Spices, herbs and flavourings** | 213,480 | 0.00 | . | 0.00 | . | 0.00 | . | 0.00 | . | 0.01 | . | 0.01 | . |
| **Condiments** | 246,786 | 0.00 | . | 0.01 | . | 0.00 | . | 0.00 | . | 0.09 | . | 0.09 | . |
| **Soups, bouillons** | 382,311 | 1.79 | . | 0.52 | . | 0.38 | . | 0.46 | . | 0.79 | . | 0.81 | . |
| **Soups** | 337,576 | 1.78 | 14 | 0.52 | 10 | 0.33 | . | 0.40 | . | 0.76 | . | 0.78 | . |
| **Bouillons** | 151,165 | 0.01 | . | 0.00 | . | 0.06 | . | 0.07 | . | 0.04 | . | 0.04 | . |
| **Miscellaneous** | 209,789 | 0.35 | . | 0.11 | . | 0.08 | . | 0.09 | . | 1.35 | . | 1.38 | . |
| **Vegetarian products and dishes** | 97,218 | 0.00 | . | 0.00 | . | 0.00 | . | 0.00 | . | 0.21 | . | 0.21 | . |
| **Soya products** | 144,054 | 0.17 | . | 0.00 | . | 0.00 | . | 0.00 | . | 0.26 | . | 0.26 | . |
| **Dietetic products** | 35,151 | 0.00 | . | 0.00 | . | 0.00 | . | 0.00 | . | 0.21 | . | 0.21 | . |
| **Snacks** | 108,785 | 0.17 | . | 0.11 | 12 | 0.08 | . | 0.09 | . | 0.54 | . | 0.55 | . |
| **Non-dairy creams, creamers** | 105,400 | 0.00 | . | 0.00 | . | 0.00 | . | 0.00 | . | 0.14 | . | 0.15 | . |

n.s. = non-specified

**Supplementary table 7b:** The contributions of the different EPIC food groups to the four NOVA categories expressed in g/day for the middle bound (MB) scenario.

|  | **N** | **NOVA G1  (g)** | | **NOVA G2  (g)** | | **NOVA G3  (g)** | | **NOVA G3 excl. ALC (g)** | | **NOVA G4  (g)** | | **NOVA G4 excl. ALC (g)** | |
| --- | --- | --- | --- | --- | --- | --- | --- | --- | --- | --- | --- | --- | --- |
| **Epic Food Classification - MB** |  | **%** | **Order** | **%** | **Order** | **%** | **Order** | **%** | **Order** | **%** | **Order** | **%** | **Order** |
| **Potatoes and other tubers** | 472,088 | 4.27 | . | 0.01 | . | 0.63 | . | 1.16 | . | 2.10 | . | 2.15 | . |
| **Potatoes** | 472,086 | 4.27 | 5 | 0.01 | . | 0.63 | . | 1.16 | 14 | 2.10 | 12 | 2.15 | 12 |
| **Other tubers** | 42 | 0.00 | . | 0.00 | . | 0.00 | . | 0.00 | . | 0.00 | . | 0.00 | . |
| **Vegetables** | 476,679 | 9.38 | . | 0.01 | . | 2.40 | . | 4.45 | . | 1.17 | . | 1.20 | . |
| **Vegetables n.s.** | 87,359 | 0.55 | . | 0.00 | . | 0.31 | . | 0.57 | . | 0.00 | . | 0.00 | . |
| **Leafy vegetables** | 422,682 | 1.36 | 12 | 0.00 | . | 0.00 | . | 0.00 | . | 0.00 | . | 0.00 | . |
| **Fruiting vegetables** | 472,318 | 2.72 | 7 | 0.00 | . | 1.06 | 12 | 1.95 | 10 | 0.49 | . | 0.50 | . |
| **Root vegetables** | 446,546 | 1.30 | 14 | 0.00 | . | 0.51 | . | 0.95 | 15 | 0.02 | . | 0.02 | . |
| **Cabbages** | 420,040 | 1.36 | 13 | 0.00 | . | 0.12 | . | 0.23 | . | 0.00 | . | 0.00 | . |
| **Mushrooms** | 416,387 | 0.27 | . | 0.00 | . | 0.04 | . | 0.07 | . | 0.11 | . | 0.11 | . |
| **Grain and pod vegetables** | 383,627 | 0.45 | . | 0.00 | . | 0.16 | . | 0.30 | . | 0.00 | . | 0.00 | . |
| **Onion, garlic** | 459,500 | 0.49 | . | 0.00 | . | 0.07 | . | 0.12 | . | 0.31 | . | 0.32 | . |
| **Stalk vegetables, sprouts** | 409,620 | 0.43 | . | 0.00 | . | 0.03 | . | 0.05 | . | 0.23 | . | 0.24 | . |
| **Mixed salad, mixed vegetables** | 208,468 | 0.45 | . | 0.01 | . | 0.11 | . | 0.21 | . | 0.00 | . | 0.00 | . |
| **Legumes** | 367,815 | 0.48 | . | 0.00 | . | 0.63 | . | 1.17 | . | 0.63 | . | 0.64 | . |
| **Legumes** | 367,815 | 0.48 | . | 0.00 | . | 0.63 | 15 | 1.17 | 13 | 0.63 | . | 0.64 | . |
| **Fruits, nuts and seeds** | 474,836 | 11.20 | **3** | 0.10 | . | 3.06 | . | 5.67 | **3** | 0.15 | . | 0.16 | . |
| **Fruit peel** | 7 | 0.00 | . | 0.00 | . | 0.00 | . | 0.00 | . | 0.00 | . | 0.00 | . |
| **Fruit** | 472,958 | 11.01 | **3** | 0.02 | . | 2.28 | 7 | 4.22 | 5 | 0.08 | . | 0.08 | . |
| **Nuts (-spread) and seeds** | 363,751 | 0.11 | . | 0.00 | . | 0.48 | . | 0.89 | . | 0.02 | . | 0.02 | . |
| **Mixed fruits** | 102,935 | 0.08 | . | 0.08 | . | 0.17 | . | 0.31 | . | 0.03 | . | 0.03 | . |
| **Olives** | 86,723 | 0.00 | . | 0.00 | . | 0.13 | . | 0.25 | . | 0.02 | . | 0.02 | . |
| **Dairy products** | 476,067 | 12.21 | **2** | 7.38 | . | 11.87 | **3** | 21.97 | **2** | 13.51 | **3** | 13.84 | **3** |
| **Dairy product n.s. or combined** | 107,002 | 0.65 | . | 0.00 | . | 0.00 | . | 0.00 | . | 1.28 | . | 1.32 | . |
| **Milk** | 424,317 | 9.34 | 4 | 0.00 | . | 0.24 | . | 0.45 | . | 0.24 | . | 0.25 | . |
| **Milk beverages** | 117,061 | 0.01 | . | 0.00 | . | 0.23 | . | 0.42 | . | 1.80 | 14 | 1.85 | 14 |
| **Yoghurt, thick fermented milk** | 386,718 | 1.81 | 10 | 0.00 | . | 0.14 | . | 0.26 | . | 6.06 | 5 | 6.21 | 5 |
| **Curd** | 250,834 | 0.31 | . | 0.00 | . | 1.00 | 13 | 1.85 | 11 | 0.31 | . | 0.32 | . |
| **Cheese** | 464,655 | 0.00 | . | 0.00 | . | 9.30 | 4 | 17.22 | **2** | 1.15 | . | 1.17 | . |
| **Cream desserts, puddings (milk based)** | 289,668 | 0.02 | . | 0.19 | 13 | 0.83 | 14 | 1.53 | 12 | 2.50 | 10 | 2.56 | 10 |
| **Dairy creams** | 372,196 | 0.00 | . | 7.19 | 5 | 0.07 | . | 0.13 | . | 0.14 | . | 0.15 | . |
| **Milk for coffee and creamers (dairy)** | 41,916 | 0.06 | . | 0.00 | . | 0.06 | . | 0.11 | . | 0.02 | . | 0.02 | . |
| **Cereal and cereal products** | 476,652 | 3.80 | . | 0.25 | . | 22.88 | **2** | 42.35 | **1** | 17.26 | **2** | 17.67 | **2** |
| **Flour, flakes, starches, semolina** | 201,868 | 0.04 | . | 0.09 | . | 0.01 | . | 0.01 | . | 0.09 | . | 0.09 | . |
| **Pasta, rice, other grains** | 461,031 | 3.07 | 6 | 0.11 | 14 | 0.00 | . | 0.01 | . | 0.56 | . | 0.58 | . |
| **Bread, crispbread, rusks** | 475,509 | 0.00 | . | 0.00 | . | 22.50 | **2** | 41.66 | **1** | 13.60 | **2** | 13.93 | **2** |
| **Breakfast cereals** | 226,520 | 0.69 | . | 0.02 | . | 0.00 | . | 0.00 | . | 1.39 | . | 1.42 | . |
| **Salty biscuits, aperitif biscuits, crackers** | 340,308 | 0.00 | . | 0.00 | . | 0.00 | . | 0.00 | . | 0.92 | . | 0.94 | . |
| **Dough and pastry (puff, short-crust, pizza)** | 311,065 | 0.00 | . | 0.03 | . | 0.36 | . | 0.67 | . | 0.70 | . | 0.71 | . |
| **Meat and meat products** | 475,705 | 3.23 | . | 0.02 | . | 1.41 | . | 2.61 | . | 8.37 | . | 8.57 | . |
| **Meat and meat product n.s. or combined** | 58,477 | 0.10 | . | 0.01 | . | 0.02 | . | 0.04 | . | 0.01 | . | 0.01 | . |
| **Red meat** | 466,661 | 2.03 | 9 | 0.01 | . | 0.02 | . | 0.03 | . | 0.64 | . | 0.66 | . |
| **Poultry** | 412,329 | 0.95 | . | 0.00 | . | 0.00 | . | 0.00 | . | 0.02 | . | 0.02 | . |
| **Game** | 25,595 | 0.01 | . | 0.00 | . | 0.00 | . | 0.00 | . | 0.00 | . | 0.00 | . |
| **Processed meat** | 469,869 | 0.02 | . | 0.00 | . | 1.37 | 10 | 2.54 | 8 | 7.69 | **3** | 7.87 | **3** |
| **Offals** | 252,743 | 0.13 | . | 0.00 | . | 0.00 | . | 0.00 | . | 0.02 | . | 0.02 | . |
| **Fish and shellfish** | 451,668 | 1.29 | . | 0.02 | . | 1.95 | . | 3.61 | . | 1.52 | . | 1.56 | . |
| **Fish and shellfish n.s. or combined** | 7,620 | 0.03 | . | 0.02 | . | 0.00 | . | 0.00 | . | 0.00 | . | 0.00 | . |
| **Fish** | 445,215 | 1.09 | 15 | 0.00 | . | 1.61 | 9 | 2.98 | 7 | 0.73 | . | 0.74 | . |
| **Crustaceans, molluscs** | 326,942 | 0.15 | . | 0.00 | . | 0.02 | . | 0.04 | . | 0.03 | . | 0.03 | . |
| **Fish products, fish in crumbs** | 287,627 | 0.03 | . | 0.00 | . | 0.32 | . | 0.60 | . | 0.77 | . | 0.79 | . |
| **Egg and egg products** | 468,194 | 0.88 | . | 0.00 | . | 0.00 | . | 0.00 | . | 0.15 | . | 0.16 | . |
| **Eggs** | 468,194 | 0.88 | . | 0.00 | . | 0.00 | . | 0.00 | . | 0.15 | . | 0.16 | . |
| **Fat** | 476,714 | 0.00 | . | 44.23 | **1** | 0.01 | . | 0.02 | . | 3.83 | . | 3.93 | . |
| **Fat n.s. or combined** | 203,568 | 0.00 | . | 1.77 | 7 | 0.00 | . | 0.00 | . | 0.21 | . | 0.22 | . |
| **Vegetable oils** | 435,880 | 0.00 | . | 26.02 | **2** | 0.00 | . | 0.01 | . | 0.06 | . | 0.06 | . |
| **Butter** | 333,189 | 0.00 | . | 15.05 | **3** | 0.00 | . | 0.00 | . | 0.00 | . | 0.00 | . |
| **Margarine** | 393,553 | 0.00 | . | 0.00 | . | 0.00 | . | 0.01 | . | 3.46 | 6 | 3.54 | 6 |
| **Deep frying fats** | 84,134 | 0.00 | . | 0.10 | 15 | 0.00 | . | 0.00 | . | 0.09 | . | 0.09 | . |
| **Marine oils** | 10,906 | 0.00 | . | 0.44 | 11 | 0.00 | . | 0.00 | . | 0.00 | . | 0.00 | . |
| **Other animal fat** | 95,619 | 0.00 | . | 0.85 | 10 | 0.00 | . | 0.00 | . | 0.01 | . | 0.01 | . |
| **Sugar and confectionary** | 465,456 | 0.00 | . | 31.21 | **2** | 2.51 | . | 4.64 | . | 5.63 | . | 5.76 | . |
| **Sugar, honey, jam and syrup** | 435,145 | 0.00 | . | 31.20 | **1** | 2.49 | 5 | 4.62 | **3** | 0.11 | . | 0.11 | . |
| **Chocolate, candy bars, paste, confetti** | 381,589 | 0.00 | . | 0.00 | . | 0.00 | . | 0.00 | . | 2.25 | 11 | 2.30 | 11 |
| **Confectionery non-chocolate, candied fruits** | 287,066 | 0.00 | . | 0.00 | . | 0.01 | . | 0.03 | . | 1.20 | . | 1.23 | . |
| **Ice cream, water ice** | 397,842 | 0.00 | . | 0.02 | . | 0.00 | . | 0.00 | . | 2.07 | 13 | 2.12 | 13 |
| **Cakes and biscuits** | 453,549 | 0.18 | . | 2.74 | . | 2.43 | . | 4.50 | . | 8.05 | . | 8.24 | . |
| **Cakes, sweet pies, pastries, puddings** | 440,595 | 0.18 | . | 2.73 | 6 | 2.43 | 6 | 4.49 | 4 | 6.26 | 4 | 6.40 | 4 |
| **Dry cakes, biscuits** | 335,297 | 0.00 | . | 0.01 | . | 0.00 | . | 0.01 | . | 1.79 | 15 | 1.83 | 15 |
| **Non alcoholic beverages** | 474,961 | 51.45 | **1** | 0.01 | . | 0.34 | . | 0.57 | . | 26.85 | **1** | 27.49 | **1** |
| **Non alcoholic beverages n.s.** | 145,175 | 0.00 | . | 0.01 | . | 0.04 | . | 0.02 | . | 0.78 | . | 0.79 | . |
| **Fruit and vegetable juices** | 375,487 | 2.66 | 8 | 0.00 | . | 0.24 | . | 0.44 | . | 3.41 | 7 | 3.49 | 7 |
| **Carbonated/soft/isotonic drinks, diluted syrups** | 298,340 | 0.00 | . | 0.00 | . | 0.00 | . | 0.00 | . | 22.33 | **1** | 22.86 | **1** |
| **Coffee, tea, herbal teas** | 466,677 | 32.17 | **1** | 0.00 | . | 0.00 | . | 0.00 | . | 0.10 | . | 0.11 | . |
| **Waters** | 303,997 | 16.61 | **2** | 0.00 | . | 0.06 | . | 0.12 | . | 0.24 | . | 0.25 | . |
| **Alcoholic beverages** | 422,090 | 0.00 | . | 0.00 | . | 45.95 | **1** | 0.00 | . | 2.51 | . | 0.18 | . |
| **Alcoholic beverages n.s. or combined** | 13,067 | 0.00 | . | 0.00 | . | 0.00 | . | 0.00 | . | 0.02 | . | 0.00 | . |
| **Wine** | 381,570 | 0.00 | . | 0.00 | . | 20.31 | **3** | 0.00 | . | 0.00 | . | 0.00 | . |
| **Fortified wines** | 238,590 | 0.00 | . | 0.00 | . | 0.00 | . | 0.00 | . | 1.08 | . | 0.00 | . |
| **Beer, cider** | 311,346 | 0.00 | . | 0.00 | . | 25.64 | **1** | 0.00 | . | 0.18 | . | 0.18 | . |
| **Spirits, brandy** | 247,318 | 0.00 | . | 0.00 | . | 0.00 | . | 0.00 | . | 0.94 | . | 0.00 | . |
| **Aniseed drinks (pastis,...)** | 41,885 | 0.00 | . | 0.00 | . | 0.00 | . | 0.00 | . | 0.04 | . | 0.00 | . |
| **Liqueurs** | 48,356 | 0.00 | . | 0.00 | . | 0.00 | . | 0.00 | . | 0.07 | . | 0.00 | . |
| **Cocktails, punches** | 22,545 | 0.00 | . | 0.00 | . | 0.00 | . | 0.00 | . | 0.18 | . | 0.00 | . |
| **Condiments and sauces** | 463,782 | 0.09 | . | 12.12 | **3** | 1.29 | . | 2.39 | . | 3.18 | . | 3.26 | . |
| **Sauces** | 461,139 | 0.09 | . | 10.88 | 4 | 1.28 | 11 | 2.37 | 9 | 3.05 | 8 | 3.12 | 8 |
| **Yeast** | 3,878 | 0.00 | . | 0.02 | . | 0.00 | . | 0.00 | . | 0.00 | . | 0.00 | . |
| **Spices, herbs and flavourings** | 213,480 | 0.00 | . | 0.00 | . | 0.00 | . | 0.00 | . | 0.01 | . | 0.01 | . |
| **Condiments** | 246,786 | 0.00 | . | 1.22 | 9 | 0.01 | . | 0.02 | . | 0.12 | . | 0.12 | . |
| **Soups, bouillons** | 382,311 | 1.45 | . | 1.64 | . | 2.60 | . | 4.82 | . | 3.44 | . | 3.53 | . |
| **Soups** | 337,576 | 1.39 | 11 | 1.64 | 8 | 2.23 | 8 | 4.12 | 6 | 2.71 | 9 | 2.77 | 9 |
| **Bouillons** | 151,165 | 0.06 | . | 0.00 | . | 0.38 | . | 0.70 | . | 0.74 | . | 0.75 | . |
| **Miscellaneous** | 209,789 | 0.09 | . | 0.24 | . | 0.04 | . | 0.07 | . | 1.63 | . | 1.67 | . |
| **Vegetarian products and dishes** | 97,218 | 0.00 | . | 0.00 | . | 0.00 | . | 0.00 | . | 0.20 | . | 0.20 | . |
| **Soya products** | 144,054 | 0.06 | . | 0.01 | . | 0.00 | . | 0.00 | . | 0.81 | . | 0.83 | . |
| **Dietetic products** | 35,151 | 0.00 | . | 0.00 | . | 0.00 | . | 0.00 | . | 0.11 | . | 0.11 | . |
| **Snacks** | 108,785 | 0.03 | . | 0.24 | 12 | 0.04 | . | 0.07 | . | 0.35 | . | 0.36 | . |
| **Non-dairy creams, creamers** | 105,400 | 0.00 | . | 0.00 | . | 0.00 | . | 0.00 | . | 0.16 | . | 0.16 | . |

n.s.: non-specified

**Supplementary table 8:** Correlations for the NOVA group 4 data (expressed in absolute and in % kcal/day) coded by the Spanish team versus those obtained via the coding performed by the international team (IARC and USP) for the Spanish food list using the three different scenarios.

|  | | **Absolute incl. alcohol (kcal/day)** | | | **Percentage incl. alcohol  (% kcal/day)** | | | **Absolute excl. alcohol (kcal/day)** | | | **Percentage excl. alcohol  (% kcal/day)** | | |
| --- | --- | --- | --- | --- | --- | --- | --- | --- | --- | --- | --- | --- | --- |
|  | **N** | **Lower bound** | **Middle bound** | **Upper bound** | **Lower bound** | **Middle bound** | **Upper bound** | **Lower bound** | **Middle bound** | **Upper bound** | **Lower bound** | **Middle bound** | **Upper bound** |
| **Pearson correlation for NOVA group 4 (the p-values are all <.0001)** | | | | | | | | | | | | | |
| **Total EPIC Spain** | 41,437 | **0.85** | **0.85** | **0.75** | **0.78** | **0.78** | **0.57** | **0.83** | **0.83** | **0.74** | **0.77** | **0.77** | **0.54** |
| **Male** | 15,629 | 0.83 | 0.83 | 0.70 | 0.74 | 0.74 | 0.48 | 0.81 | 0.81 | 0.68 | 0.72 | 0.72 | 0.44 |
| **Female** | 25,808 | 0.87 | 0.87 | 0.78 | 0.80 | 0.80 | 0.61 | 0.86 | 0.87 | 0.78 | 0.80 | 0.80 | 0.60 |
| **Asturias** | 8,542 | **0.82** | **0.82** | **0.76** | **0.76** | **0.76** | **0.63** | **0.81** | **0.81** | **0.75** | **0.75** | **0.75** | **0.60** |
| **Male** | 3,083 | 0.81 | 0.81 | 0.71 | 0.73 | 0.73 | 0.55 | 0.79 | 0.80 | 0.69 | 0.71 | 0.71 | 0.50 |
| **Female** | 5,459 | 0.84 | 0.84 | 0.79 | 0.77 | 0.77 | 0.66 | 0.84 | 0.84 | 0.78 | 0.77 | 0.77 | 0.65 |
| **Granada** | 7,879 | **0.94** | **0.94** | **0.85** | **0.84** | **0.84** | **0.60** | **0.94** | **0.94** | **0.85** | **0.83** | **0.84** | **0.59** |
| **Male** | 1,796 | 0.96 | 0.96 | 0.88 | 0.82 | 0.83 | 0.56 | 0.96 | 0.96 | 0.87 | 0.81 | 0.81 | 0.54 |
| **Female** | 6,083 | 0.92 | 0.92 | 0.82 | 0.84 | 0.85 | 0.61 | 0.92 | 0.92 | 0.82 | 0.84 | 0.84 | 0.61 |
| **Murcia** | 8,515 | **0.86** | **0.86** | **0.76** | **0.82** | **0.82** | **0.62** | **0.85** | **0.85** | **0.75** | **0.81** | **0.81** | **0.59** |
| **Male** | 2,684 | 0.82 | 0.83 | 0.72 | 0.77 | 0.77 | 0.54 | 0.79 | 0.80 | 0.70 | 0.75 | 0.75 | 0.51 |
| **Female** | 5,831 | 0.88 | 0.88 | 0.79 | 0.84 | 0.84 | 0.65 | 0.88 | 0.88 | 0.79 | 0.83 | 0.84 | 0.64 |
| **Navarra** | 8,084 | **0.82** | **0.82** | **0.70** | **0.76** | **0.76** | **0.55** | **0.78** | **0.79** | **0.69** | **0.74** | **0.75** | **0.50** |
| **Male** | 3,908 | 0.79 | 0.79 | 0.65 | 0.72 | 0.72 | 0.46 | 0.74 | 0.75 | 0.62 | 0.68 | 0.68 | 0.40 |
| **Female** | 4,176 | 0.84 | 0.85 | 0.78 | 0.77 | 0.77 | 0.61 | 0.84 | 0.84 | 0.78 | 0.76 | 0.77 | 0.61 |
| **San Sebastian** | 8,417 | **0.77** | **0.77** | **0.65** | **0.72** | **0.72** | **0.48** | **0.74** | **0.75** | **0.63** | **0.71** | **0.71** | **0.44** |
| **Male** | 4,158 | 0.75 | 0.75 | 0.57 | 0.69 | 0.69 | 0.37 | 0.73 | 0.73 | 0.55 | 0.66 | 0.67 | 0.33 |
| **Female** | 4,259 | 0.80 | 0.81 | 0.72 | 0.75 | 0.75 | 0.56 | 0.80 | 0.80 | 0.71 | 0.74 | 0.75 | 0.55 |

|  | | **Absolute incl. alcohol (kcal/day)** | | | **Percentage incl. alcohol  (% kcal/day)** | | | **Absolute excl. alcohol (kcal/day)** | | | **Percentage excl. alcohol  (% kcal/day)** | | |
| --- | --- | --- | --- | --- | --- | --- | --- | --- | --- | --- | --- | --- | --- |
|  | **N** | **Lower bound** | **Middle bound** | **Upper bound** | **Lower bound** | **Middle bound** | **Upper bound** | **Lower bound** | **Middle bound** | **Upper bound** | **Lower bound** | **Middle bound** | **Upper bound** |
| **Spearman correlation for NOVA group 4 (the p-values are all <.0001)** | | | | | | | | | | | | | |
| **Total EPIC Spain** | 41,437 | **0.82** | **0.82** | **0.71** | **0.77** | **0.78** | **0.54** | **0.80** | **0.80** | **0.71** | **0.76** | **0.77** | **0.51** |
| **Male** | 15,629 | 0.78 | 0.78 | 0.62 | 0.72 | 0.73 | 0.45 | 0.75 | 0.75 | 0.60 | 0.70 | 0.71 | 0.42 |
| **Female** | 25,808 | 0.85 | 0.85 | 0.75 | 0.80 | 0.80 | 0.59 | 0.85 | 0.85 | 0.74 | 0.80 | 0.80 | 0.58 |
| **Asturias** | 8,542 | **0.80** | **0.80** | **0.76** | **0.75** | **0.75** | **0.60** | **0.79** | **0.79** | **0.75** | **0.74** | **0.74** | **0.57** |
| **Male** | 3,083 | 0.78 | 0.78 | 0.70 | 0.71 | 0.72 | 0.53 | 0.76 | 0.76 | 0.68 | 0.69 | 0.69 | 0.48 |
| **Female** | 5,459 | 0.82 | 0.82 | 0.78 | 0.76 | 0.77 | 0.64 | 0.82 | 0.82 | 0.77 | 0.76 | 0.76 | 0.63 |
| **Granada** | 7,879 | **0.88** | **0.88** | **0.73** | **0.84** | **0.84** | **0.57** | **0.87** | **0.88** | **0.73** | **0.83** | **0.84** | **0.56** |
| **Male** | 1,796 | 0.86 | 0.86 | 0.70 | 0.81 | 0.82 | 0.51 | 0.85 | 0.86 | 0.69 | 0.80 | 0.80 | 0.49 |
| **Female** | 6,083 | 0.88 | 0.89 | 0.73 | 0.84 | 0.85 | 0.58 | 0.88 | 0.88 | 0.73 | 0.84 | 0.85 | 0.58 |
| **Murcia** | 8,515 | **0.87** | **0.88** | **0.75** | **0.82** | **0.83** | **0.60** | **0.86** | **0.86** | **0.75** | **0.82** | **0.82** | **0.58** |
| **Male** | 2,684 | 0.84 | 0.84 | 0.69 | 0.78 | 0.79 | 0.53 | 0.81 | 0.81 | 0.67 | 0.77 | 0.77 | 0.49 |
| **Female** | 5,831 | 0.89 | 0.89 | 0.78 | 0.84 | 0.84 | 0.63 | 0.89 | 0.89 | 0.78 | 0.84 | 0.84 | 0.62 |
| **Navarra** | 8,084 | **0.81** | **0.81** | **0.69** | **0.76** | **0.76** | **0.53** | **0.77** | **0.77** | **0.68** | **0.74** | **0.74** | **0.48** |
| **Male** | 3,908 | 0.78 | 0.78 | 0.62 | 0.72 | 0.72 | 0.45 | 0.73 | 0.73 | 0.59 | 0.68 | 0.68 | 0.39 |
| **Female** | 4,176 | 0.84 | 0.84 | 0.77 | 0.77 | 0.77 | 0.60 | 0.83 | 0.84 | 0.77 | 0.77 | 0.77 | 0.59 |
| **San Sebastian** | 8,417 | **0.74** | **0.75** | **0.64** | **0.70** | **0.71** | **0.45** | **0.71** | **0.71** | **0.62** | **0.69** | **0.70** | **0.42** |
| **Male** | 4,158 | 0.72 | 0.72 | 0.53 | 0.66 | 0.67 | 0.35 | 0.69 | 0.69 | 0.51 | 0.64 | 0.65 | 0.32 |
| **Female** | 4,259 | 0.78 | 0.78 | 0.70 | 0.74 | 0.74 | 0.55 | 0.77 | 0.78 | 0.69 | 0.74 | 0.74 | 0.54 |

**Supplementary table 9:** Partial correlations of elaidic acid with intakes of the 4 different NOVA groups and for the lower, middle and upper bound scenarios expressed as g/day, kcal/day, %g/day and %kcal/day including or excluding alcohol. Partial correlations are adjusted for sex, age, BMI and country (N=9,460).

|  | **Partial Pearson correlation Coefficient (adjusted for sex, age, BMI and country)** | | | | | | **Partial Spearman correlation Coefficient (adjusted for sex, age, BMI and country)** | | | | | | **Partial Pearson correlation coefficient (adjusted for sex, age and BMI)** | | **Partial Spearman correlation coefficient (adjusted for sex, age and BMI)** | |
| --- | --- | --- | --- | --- | --- | --- | --- | --- | --- | --- | --- | --- | --- | --- | --- | --- |
|  | **Lower bound** | | **Middle bound** | | **Upper bound** | | **Lower bound** | | **Middle bound** | | **Upper bound** | | **Middle bound** | | **Middle bound** | |
|  | **R** | **p-value** | **R** | **p-value** | **R** | **p-value** | **R** | **p-value** | **R** | **p-value** | **R** | **p-value** | **R** | **p-value** | **R** | **p-value** |
| **Expressed in g/day** |  |  |  |  |  |  |  |  |  |  |  |  |  |  |  |  |
| Unprocessed or minimally processed foods -G1 | -0.04 | 0.0002 | **-0.04** | 0.0001 | -0.04 | <.0001 | -0.03 | 0.002 | **-0.03** | 0.001 | -0.03 | 0.0009 | **0.17** | <.0001 | **0.22** | <.0001 |
| Processed culinary ingredients -G2 | -0.03 | 0.002 | **-0.05** | <.0001 | -0.05 | <.0001 | -0.04 | 0.0002 | **-0.07** | <.0001 | -0.07 | <.0001 | **-0.36** | <.0001 | **-0.42** | <.0001 |
| Processed foods -G3 | -0.16 | <.0001 | **-0.15** | <.0001 | -0.17 | <.0001 | -0.15 | <.0001 | **-0.15** | <.0001 | -0.18 | <.0001 | **-0.31** | <.0001 | **-0.35** | <.0001 |
| Processed foods -G3 excl. alcohol intake | 0.005 | 0.64 | **0.009** | 0.41 | -0.005 | 0.65 | 0.003 | 0.77 | **0.02** | 0.069 | -0.008 | 0.45 | **-0.27** | 0.002 | **-0.29** | <.0001 |
| Ultra-processed foods -G4 | 0.08 | <.0001 | **0.09** | <.0001 | 0.08 | <.0001 | 0.10 | <.0001 | **0.10** | <.0001 | 0.09 | <.0001 | **0.37** | <.0001 | **0.43** | <.0001 |
| Ultra-processed foods -G4 excl. alcohol intake | 0.19 | <.0001 | **0.10** | <.0001 | 0.09 | <.0001 | 0.12 | <.0001 | **0.12** | <.0001 | 0.10 | <.0001 | **0.37** | <.0001 | **0.44** | <.0001 |
| **Expressed in kcal/day** |  |  |  |  |  |  |  |  |  |  |  |  |  |  |  |  |
| Unprocessed or minimally processed foods -G1 | -0.05 | <.0001 | **-0.06** | 0.0001 | -0.07 | <.0001 | -0.05 | <.0001 | **-0.06** | 0.001 | -0.06 | <.0001 | **-0.10** | <.0001 | **-0.09** | <.0001 |
| Processed culinary ingredients -G2 | -0.07 | <.0001 | **-0.08** | <.0001 | -0.09 | <.0001 | -0.08 | <.0001 | **-0.10** | <.0001 | -0.10 | <.0001 | **-0.41** | <.0001 | **-0.48** | <.0001 |
| Processed foods -G3 | -0.08 | <.0001 | **-0.07** | <.0001 | -0.12 | <.0001 | -0.08 | <.0001 | **-0.07** | <.0001 | -0.14 | <.0001 | **-0.29** | <.0001 | **-0.29** | <.0001 |
| Processed foods -G3 excl. alcohol intake | 0.005 | 0.64 | **0.008** | 0.42 | -0.009 | 0.40 | 0.005 | 0.64 | **0.01** | 0.23 | -0.01 | 0.19 | **-0.23** | <.0001 | **-0.24** | <.0001 |
| Ultra-processed foods -G4 | 0.17 | <.0001 | **0.17** | <.0001 | 0.14 | <.0001 | 0.17 | <.0001 | **0.17** | <.0001 | 0.14 | <.0001 | **0.45** | <.0001 | **0.47** | <.0001 |
| Ultra-processed foods -G4 excl. alcohol intake | 0.19 | <.0001 | **0.19** | <.0001 | 0.15 | <.0001 | 0.19 | <.0001 | **0.19** | <.0001 | 0.15 | <.0001 | **0.45** | <.0001 | **0.47** | <.0001 |
| **Expressed in % of g/day incl. alcohol intake** |  |  |  |  |  |  |  |  |  |  |  |  |  |  |  |  |
| Unprocessed or minimally processed foods -G1 | 0.03 | 0.005 | **0.02** | 0.07 | 0.01 | 0.28 | 0.03 | 0.006 | **0.02** | 0.07 | 0.01 | 0.24 | **0.13** | <.0001 | **0.13** | <.0001 |
| Processed culinary ingredients -G2 | -0.006 | 0.56 | **-0.03** | 0.0046 | -0.03 | 0.003 | -0.01 | 0.33 | **-0.03** | 0.002 | -0.03 | 0.001 | **-0.37** | <.0001 | **-0.38** | <.0001 |
| Processed foods -G3 | -0.14 | <.0001 | **-0.14** | <.0001 | -0.17 | <.0001 | -0.15 | <.0001 | **-0.14** | <.0001 | -0.17 | <.0001 | **-0.37** | <.0001 | **-0.37** | <.0001 |
| Ultra-processed foods -G4 | 0.11 | <.0001 | **0.12** | <.0001 | 0.12 | <.0001 | 0.12 | <.0001 | **0.12** | <.0001 | 0.13 | <.0001 | **0.31** | <.0001 | **0.32** | <.0001 |

| **Expressed in % of kcal/day incl. alcohol intake** |  |  |  |  |  |  | |  | |  | |  | |  |  | |  | | |  | | |  | | |  | | |  | | |  |
| --- | --- | --- | --- | --- | --- | --- | --- | --- | --- | --- | --- | --- | --- | --- | --- | --- | --- | --- | --- | --- | --- | --- | --- | --- | --- | --- | --- | --- | --- | --- | --- | --- |
| Unprocessed or minimally processed foods -G1 | -0.07 | <.0001 | **-0.08** | <.0001 | -0.08 | | <.0001 | | -0.07 | | <.0001 | | **-0.08** | <.0001 | -0.08 | <.0001 | | | **-0.09** | | | <.0001 | | | **-0.09** | | | <.0001 | | |  |  |
| Processed culinary ingredients -G2 | -0.07 | <.0001 | **-0.09** | <.0001 | -0.09 | | <.0001 | | -0.08 | | <.0001 | | **-0.09** | <.0001 | -0.09 | | | <.0001 | | | **-0.44** | | | <.0001 | | | **-0.45** | | | <.0001 | | |
| Processed foods -G3 | -0.10 | <.0001 | **-0.10** | <.0001 | -0.16 | | <.0001 | | -0.11 | | <.0001 | | **-0.11** | <.0001 | -0.16 | | | <.0001 | | | **-0.32** | | | <.0001 | | | **-0.32** | | | <.0001 | | |
| Ultra-processed foods -G4 | 0.21 | <.0001 | **0.22** | <.0001 | 0.22 | | <.0001 | | 0.22 | | <.0001 | | **0.22** | <.0001 | 0.21 | | | <.0001 | | | **0.52** | | | <.0001 | | | **0.53** | | | <.0001 | | |
| **Expressed in % of g/day excl. alcohol intake** |  |  |  |  |  |  | |  | |  | |  | |  |  | |  | | |  | | |  | | |  | | |  | | |  |
| Unprocessed or minimally processed foods -G1 | -0.09 | <.0001 | **-0.09** | <.0001 | **-0.09** | | **<.0001** | | -0.09 | | <.0001 | | **-0.09** | 0.001 | -0.09 | | | <.0001 | | | **0.03** | | | 0.0006 | | | **0.04** | | | 0.0002 | | |
| Processed culinary ingredients -G2 | -0.03 | 0.0007 | **-0.06** | <.0001 | **-0.06** | | **<.0001** | | -0.04 | | 0.0001 | | **-0.06** | <.0001 | -0.06 | | | <.0001 | | | **-0.39** | | | <.0001 | | | **-0.39** | | | <.0001 | | |
| Processed foods -G3 | -0.008 | 0.47 | **-0.002** | 0.82 | **-0.01** | | **0.34** | | -0.01 | | 0.27 | | **-0.006** | 0.54 | -0.02 | | | 0.14 | | | **-0.32** | | | <.0001 | | | **-0.33** | | | <.0001 | | |
| Ultra-processed foods -G4 | 0.11 | <.0001 | **0.12** | <.0001 | **0.10** | | **<.0001** | | 0.11 | | <.0001 | | **0.12** | <.0001 | 0.10 | | | <.0001 | | | **0.30** | | | <.0001 | | | **0.30** | | | <.0001 | | |
| **Expressed in % of kcal/day excl. alcohol intake** |  |  |  |  |  |  | |  | |  | |  | |  |  | |  | | |  | | |  | | |  | | |  | | |  |
| Unprocessed or minimally processed foods -G1 | -0.13 | 0.001 | **-0.14** | 0.0001 | -0.14 | | <.0001 | | -0.12 | | 0.001 | | **-0.13** | 0.001 | -0.13 | | | <.0001 | | | **-0.13** | | | <.0001 | | | **-0.13** | | | <.0001 | | |
| Processed culinary ingredients -G2 | -0.10 | <.0001 | **-0.12** | <.0001 | -0.12 | | <.0001 | | -0.11 | | <.0001 | | **-0.12** | <.0001 | -0.12 | | | <.0001 | | | **-0.46** | | | <.0001 | | | **-0.46** | | | <.0001 | | |
| Processed foods -G3 | -0.03 | 0.002 | **-0.02** | 0.02 | -0.04 | | 0.0002 | | -0.04 | | 0.0002 | | **-0.03** | 0.003 | -0.04 | | | 0.0001 | | | **-0.28** | | | <.0001 | | | **-0.28** | | | <.0001 | | |
| Ultra-processed foods -G4 | 0.21 | <.0001 | **0.22** | <.0001 | 0.19 | | <.0001 | | 0.21 | | <.0001 | | **0.21** | <.0001 | 0.19 | | | <.0001 | | | **0.51** | | | <.0001 | | | **0.52** | | | <.0001 | | |

**Supplementary table 10:** Partial correlations of urinary methyl syringol sulfate with intakes of the 4 different NOVA groups and for the lower, middle and upper bound scenarios expressed as g/day, kcal/day, % of g/day and % of kcal/day including or excluding alcohol. Partial correlations are adjusted for sex, age, BMI and country (N=417).

|  | **Partial Pearson correlation Coefficient (adjusted for sex, age, BMI and country)** | | | | | | **Partial Spearman correlation Coefficient (adjusted for sex, age, BMI and country)** | | | | | | **Partial Pearson correlation coefficient (adjusted for sex, age and BMI)** | | **Partial Spearman correlation coefficient (adjusted for sex, age and BMI)** | |
| --- | --- | --- | --- | --- | --- | --- | --- | --- | --- | --- | --- | --- | --- | --- | --- | --- |
|  | **Lower bound** | | **Middle bound** | | **Upper bound** | | **Lower bound** | | **Middle bound** | | **Upper bound** | | **Middle bound** | | **Middle bound** | |
|  | **R** | **p-value** | **R** | **p-value** | **R** | **p-value** | **R** | **p-value** | **R** | **p-value** | **R** | **p-value** | **R** | **p-value** | **R** | **p-value** |
| **Expressed in g/day** |  |  |  |  |  |  |  |  |  |  |  |  |  |  |  |  |
| Unprocessed or minimally processed foods -G1 | 0.009 | 0.85 | **0.02** | 0.70 | 0.01 | 0.83 | 0.01 | 0.82 | 0.03 | 0.52 | 0.01 | 0.79 | **0.19** | <.0001 | **0.24** | <.0001 |
| Processed culinary ingredients -G2 | 0.03 | 0.55 | **0.03** | 0.50 | 0.03 | 0.48 | -0.01 | 0.76 | -0.01 | 0.79 | -0.01 | 0.84 | **-0.20** | <.0001 | **-0.30** | <.0001 |
| Processed foods -G3 | 0.07 | 0.15 | **0.05** | 0.30 | 0.06 | 0.20 | 0.11 | 0.03 | 0.08 | 0.13 | 0.10 | 0.04 | **0.07** | 0.14 | **0.04** | 0.40 |
| Processed foods -G3 excl. alcohol intake | 0.07 | 0.13 | **0.01** | 0.76 | 0.06 | 0.22 | 0.07 | 0.13 | 0.008 | 0.88 | 0.10 | 0.03 | **-0.12** | 0.01 | **-0.11** | 0.03 |
| Ultra-processed foods -G4 | 0.05 | 0.29 | **0.05** | 0.33 | 0.05 | 0.26 | 0.07 | 0.18 | 0.06 | 0.23 | 0.05 | 0.30 | **0.31** | <.0001 | **0.35** | <.0001 |
| Ultra-processed foods -G4 excl. alcohol intake | 0.05 | 0.24 | **0.01** | 0.38 | 0.05 | 0.31 | 0.05 | 0.28 | 0.05 | 0.34 | 0.04 | 0.41 | **0.32** | <.0001 | **0.34** | <.0001 |
| **Expressed in kcal/day** |  |  |  |  |  |  |  |  |  |  |  |  |  |  |  |  |
| Unprocessed or minimally processed foods -G1 | 0.08 | 0.10 | **0.10** | 0.04 | 0.09 | 0.06 | 0.08 | 0.10 | **0.10** | 0.04 | 0.09 | 0.06 | **-0.23** | <.0001 | **-0.26** | <.0001 |
| Processed culinary ingredients -G2 | 0.006 | 0.89 | **0.009** | 0.86 | 0.01 | 0.83 | -0.03 | 0.59 | **-0.02** | 0.65 | -0.02 | 0.67 | **-0.31** | <.0001 | **-0.36** | <.0001 |
| Processed foods -G3 | 0.07 | 0.17 | **0.06** | 0.22 | 0.06 | 0.22 | 0.10 | 0.05 | **0.09** | 0.07 | 0.11 | 0.02 | **0.002** | 0.97 | **0.04** | 0.38 |
| Processed foods -G3 excl. alcohol intake | 0.06 | 0.24 | **0.05** | 0.33 | 0.04 | 0.37 | 0.06 | 0.19 | **0.06** | 0.23 | 0.10 | 0.04 | **-0.003** | 0.95 | **0.04** | 0.40 |
| Ultra-processed foods -G4 | 0.07 | 0.14 | **0.07** | 0.17 | 0.07 | 0.13 | 0.08 | 0.09 | **0.08** | 0.12 | 0.08 | 0.10 | **0.35** | <.0001 | **0.38** | <.0001 |
| Ultra-processed foods -G4 excl. alcohol intake | 0.06 | 0.20 | **0.06** | 0.23 | 0.07 | 0.17 | 0.07 | 0.17 | **0.06** | 0.22 | 0.07 | 0.14 | **0.34** | <.0001 | **0.37** | <.0001 |
| **Expressed in % of g/day incl. alcohol intake** |  |  |  |  |  |  |  |  |  |  |  |  |  |  |  |  |
| Unprocessed or minimally processed foods -G1 | -0.07 | 0.15 | **-0.03** | 0.52 | -0.04 | 0.35 | -0.08 | 0.10 | **-0.03** | 0.54 | -0.05 | 0.27 | **0.02** | 0.61 | **0.02** | 0.65 |
| Processed culinary ingredients -G2 | -0.03 | 0.60 | **-0.02** | 0.72 | -0.01 | 0.79 | -0.05 | 0.32 | **-0.04** | 0.37 | -0.04 | 0.44 | **-0.37** | <.0001 | **-0.40** | <.0001 |
| Processed foods -G3 | 0.08 | 0.09 | **0.04** | 0.42 | 0.08 | 0.09 | 0.08 | 0.09 | **0.03** | 0.59 | 0.08 | 0.09 | **-0.15** | 0.003 | **-0.15** | 0.002 |
| Ultra-processed foods -G4 | -0.002 | 0.97 | **-0.006** | 0.91 | -0.03 | 0.56 | 0.03 | 0.48 | **0.03** | 0.57 | -0.01 | 0.82 | **0.22** | <.0001 | **0.25** | <.0001 |

| **Expressed in % of kcal/day incl. alcohol intake** |  |  |  |  |  |  | |  | |  | |  | |  |  |  | |  | |  | |  | |  | |  |
| --- | --- | --- | --- | --- | --- | --- | --- | --- | --- | --- | --- | --- | --- | --- | --- | --- | --- | --- | --- | --- | --- | --- | --- | --- | --- | --- |
| Unprocessed or minimally processed foods -G1 | 0.007 | 0.89 | **0.03** | 0.53 | 0.02 | | 0.69 | | -0.009 | | 0.86 | | **0.01** | 0.81 | 0.01 | | 0.81 | | **-0.30** | | <.0001 | | **-0.33** | | <.0001 | |
| Processed culinary ingredients -G2 | -0.06 | 0.19 | **-0.06** | 0.22 | -0.06 | | 0.25 | | -0.10 | | 0.04 | | **-0.10** | 0.05 | -0.09 | | 0.05 | | **-0.37** | | <.0001 | | **-0.40** | | <.0001 | |
| Processed foods -G3 | 0.02 | 0.74 | **0.003** | 0.95 | 0.02 | | 0.63 | | 0.03 | | 0.55 | | **0.01** | 0.82 | 0.06 | | 0.25 | | **0.02** | | 0.65 | | **0.05** | | 0.32 | |
| Ultra-processed foods -G4 | 0.007 | 0.88 | **0.0009** | 0.99 | -0.009 | | 0.86 | | 0.01 | | 0.79 | | **0.004** | 0.93 | -0.006 | | 0.91 | | **0.39** | | <.0001 | | **0.41** | | <.0001 | |
| **Expressed in % of g/day excl. alcohol intake** |  |  |  |  |  |  | |  | |  | |  | |  |  |  | |  | |  | |  | |  | |  |
| Unprocessed or minimally processed foods -G1 | -0.03 | 0.53 | **0.02** | 0.75 | -0.004 | | 0.94 | | -0.04 | | 0.43 | | **0.02** | 0.71 | -0.01 | | 0.78 | | **0.07** | | 0.16 | | **0.07** | | 0.16 | |
| Processed culinary ingredients -G2 | -0.0006 | 0.99 | **0.006** | 0.90 | 0.01 | | 0.82 | | -0.02 | | 0.63 | | **-0.02** | 0.67 | -0.01 | | 0.77 | | **-0.36** | | <.0001 | | **-0.39** | | <.0001 | |
| Processed foods -G3 | 0.04 | 0.36 | **-0.03** | 0.55 | 0.04 | | 0.41 | | 0.05 | | 0.36 | | **-0.02** | 0.72 | 0.09 | | 0.07 | | **-0.30** | | <.0001 | | **-0.30** | | <.0001 | |
| Ultra-processed foods -G4 | 0.007 | 0.89 | **0.004** | 0.94 | -0.01 | | 0.81 | | 0.04 | | 0.43 | | **0.03** | 0.50 | -0.001 | | 0.99 | | **0.24** | | <.0001 | | **0.27** | | <.0001 | |
| **Expressed in % of kcal/day excl. alcohol intake** |  |  |  |  |  |  | |  | |  | |  | |  |  |  | |  | |  | |  | |  | |  |
| Unprocessed or minimally processed foods -G1 | 0.02 | 0.62 | **0.05** | 0.34 | 0.04 | | 0.46 | | 0.008 | | 0.87 | | **0.03** | 0.59 | 0.02 | | 0.63 | | **-0.29** | | <.0001 | | **-0.32** | | <.0001 | |
| Processed culinary ingredients -G2 | -0.05 | 0.30 | **-0.05** | 0.34 | -0.04 | | 0.38 | | -0.09 | | 0.06 | | **-0.09** | 0.08 | -0.08 | | 0.10 | | **-0.37** | | <.0001 | | **-0.40** | | <.0001 | |
| Processed foods -G3 | 0.003 | 0.95 | **-0.01** | 0.83 | 0.005 | | 0.92 | | 0.006 | | 0.89 | | **-0.007** | 0.89 | 0.07 | | 0.13 | | **0.02** | | 0.76 | | **0.04** | | 0.40 | |
| Ultra-processed foods -G4 | 0.0002 | 0.99 | **-0.006** | 0.90 | -0.01 | | 0.82 | | -0.002 | | 0.97 | | **-0.008** | 0.87 | -0.02 | | 0.66 | | **0.40** | | <.0001 | | **0.41** | | <.0001 | |

# Supplementary Figures

# Supplementary Figure 1a: Distributions of the different NOVA categories by Country, group (G) 1 to G 4 using the three different scenarios (Lower, Middle and Upper bound) expressed in % g/day.

**Supplementary Figure 1b:** Distributions of the different NOVA categories by Country, group (G) 1 to G 4 using the three different scenarios (Lower, Middle and Upper bound) expressed in % kcal/day.

**References**

1. Riboli E, Hunt KJ, Slimani N, Ferrari P, Norat T, Fahey M, et al. European Prospective Investigation into Cancer and Nutrition (EPIC): study populations and data collection. Public health nutrition. 2002;5(6b):1113-24.

2. van Liere MJ, Lucas F, Clavel F, Slimani N, Villeminot S. Relative validity and reproducibility of a French dietary history questionnaire. International journal of epidemiology. 1997;26 Suppl 1:S128-36.

3. Kesse E, Clavel-Chapelon F, Slimani N, van Liere M. Do eating habits differ according to alcohol consumption? Results of a study of the French cohort of the European Prospective Investigation into Cancer and Nutrition (E3N-EPIC). The American journal of clinical nutrition. 2001;74(3):322-7.

4. Pala V, Sieri S, Palli D, Salvini S, Berrino F, Bellegotti M, et al. Diet in the Italian EPIC cohorts: presentation of data and methodological issues. Tumori. 2003;89(6):594-607.

5. Sieri S, Krogh V, Muti P, Micheli A, Pala V, Crosignani P, et al. Fat and protein intake and subsequent breast cancer risk in postmenopausal women. Nutrition and cancer. 2002;42(1):10-7.

6. Sieri S, Krogh V, Pala V, Muti P, Micheli A, Evangelista A, et al. Dietary patterns and risk of breast cancer in the ORDET cohort. Cancer Epidemiol Biomarkers Prev. 2004;13(4):567-72.

7. Pisani P, Faggiano F, Krogh V, Palli D, Vineis P, Berrino F. Relative validity and reproducibility of a food frequency dietary questionnaire for use in the Italian EPIC centres. International journal of epidemiology. 1997;26 Suppl 1:S152-60.

8. Relative validity and reproducibility of a diet history questionnaire in Spain. I. Foods. EPIC Group of Spain. European Prospective Investigation into Cancer and Nutrition. International journal of epidemiology. 1997;26 Suppl 1:S91-9.

9. Agudo A, Amiano P, Barcos A, Barricarte A, Beguiristain JM, Chirlaque MD, et al. Dietary intake of vegetables and fruits among adults in five regions of Spain. EPIC Group of Spain. European Prospective Investigation into Cancer and Nutrition. Eur J Clin Nutr. 1999;53(3):174-80.

10. Bingham SA, Welch AA, McTaggart A, Mulligan AA, Runswick SA, Luben R, et al. Nutritional methods in the European Prospective Investigation of Cancer in Norfolk. Public Health Nutr. 2001;4(3):847-58.

11. Davey GK, Spencer EA, Appleby PN, Allen NE, Knox KH, Key TJ. EPIC-Oxford: lifestyle characteristics and nutrient intakes in a cohort of 33 883 meat-eaters and 31 546 non meat-eaters in the UK. Public Health Nutr. 2003;6(3):259-69.

12. Ocké MC, Bueno-de-Mesquita HB, Goddijn HE, Jansen A, Pols MA, van Staveren WA, et al. The Dutch EPIC food frequency questionnaire. I. Description of the questionnaire, and relative validity and reproducibility for food groups. International journal of epidemiology. 1997;26 Suppl 1:S37-48.

13. Katsouyanni K, Rimm EB, Gnardellis C, Trichopoulos D, Polychronopoulos E, Trichopoulou A. Reproducibility and relative validity of an extensive semi-quantitative food frequency questionnaire using dietary records and biochemical markers among Greek schoolteachers. International journal of epidemiology. 1997;26 Suppl 1:S118-27.

14. Gnardellis C, Trichopoulou A, Katsouyanni K, Polychronopoulos E, Rimm EB, Trichopoulos D. Reproducibility and validity of an extensive semiquantitative food frequency questionnaire among Greek school teachers. Epidemiology. 1995;6(1):74-7.

15. Kroke A, Klipstein-Grobusch K, Voss S, Möseneder J, Thielecke F, Noack R, et al. Validation of a self-administered food-frequency questionnaire administered in the European Prospective Investigation into Cancer and Nutrition (EPIC) Study: comparison of energy, protein, and macronutrient intakes estimated with the doubly labeled water, urinary nitrogen, and repeated 24-h dietary recall methods. The American journal of clinical nutrition. 1999;70(4):439-47.

16. Johansson I, Hallmans G, Wikman A, Biessy C, Riboli E, Kaaks R. Validation and calibration of food-frequency questionnaire measurements in the Northern Sweden Health and Disease cohort. Public Health Nutr. 2002;5(3):487-96.

17. Wirfält E, Mattisson I, Gullberg B, Berglund G. Food patterns defined by cluster analysis and their utility as dietary exposure variables: a report from the Malmö Diet and Cancer Study. Public Health Nutr. 2000;3(2):159-73.

18. Tjønneland A, Olsen A, Boll K, Stripp C, Christensen J, Engholm G, et al. Study design, exposure variables, and socioeconomic determinants of participation in Diet, Cancer and Health: a population-based prospective cohort study of 57,053 men and women in Denmark. Scand J Public Health. 2007;35(4):432-41.

19. Brustad M, Skeie G, Braaten T, Slimani N, Lund E. Comparison of telephone vs face-to-face interviews in the assessment of dietary intake by the 24 h recall EPIC SOFT program--the Norwegian calibration study. Eur J Clin Nutr. 2003;57(1):107-13.

20. Hjartaker A, Andersen LF, Lund E. Comparison of diet measures from a food-frequency questionnaire with measures from repeated 24-hour dietary recalls. The Norwegian Women and Cancer Study. Public Health Nutrition. 2007;10(10):1094-103.

21. Romaguera D, Norat T, Vergnaud AC, Mouw T, May AM, Agudo A, et al. Mediterranean dietary patterns and prospective weight change in participants of the EPIC-PANACEA project. The American journal of clinical nutrition. 2010;92(4):912-21.

22. Deschasaux M, Huybrechts I, Murphy N, Julia C, Hercberg S, Srour B, et al. Nutritional quality of food as represented by the FSAm-NPS nutrient profiling system underlying the Nutri-Score label and cancer risk in Europe: Results from the EPIC prospective cohort study. PLoS medicine. 2018;15(9):e1002651.
